# The Transcriptome Profile of the Mosquito *Culex quinquefasciatus* following Permethrin Selection

**DOI:** 10.1371/journal.pone.0047163

**Published:** 2012-10-05

**Authors:** William R. Reid, Lee Zhang, Feng Liu, Nannan Liu

**Affiliations:** 1 Department of Entomology and Plant Pathology, Auburn University, Auburn, Alabama, United States of America; 2 Genomics and Sequencing Laboratory, Auburn University, Auburn, Alabama, United States of America; University of Kentucky, United States of America

## Abstract

To gain valuable insights into the gene interaction and the complex regulation system involved in the development of insecticide resistance in mosquitoes *Culex quinquefasciatus*, we conducted a whole transcriptome analysis of *Culex* mosquitoes following permethrin selection. Gene expression profiles for the lower resistant parental mosquito strain HAmCq^G0^ and their permethrin-selected high resistant offspring HAmCq^G8^ were compared and a total of 367 and 3982 genes were found to be up- and down-regulated, respectively, in HAmCq^G8^, indicating that multiple genes are involved in response to permethrin selection. However, a similar overall cumulative gene expression abundance was identified between up- and down-regulated genes in HAmCq^G8^ mosquitoes following permethrin selection, suggesting a homeostatic response to insecticides through a balancing of the up- and down-regulation of the genes. While structural and/or cuticular structural functions were the only two enriched GO terms for down-regulated genes, the enriched GO terms obtained for the up-regulated genes occurred primarily among the catalytic and metabolic functions where they represented three functional categories: electron carrier activity, binding, and catalytic activity. Interestingly, the functional GO terms in these three functional categories were overwhelmingly overrepresented in P450s and proteases/serine proteases. The important role played by P450s in the development of insecticide resistance has been extensively studied but the function of proteases/serine proteases in resistance is less well understood. Hence, the characterization of the functions of these proteins, including their digestive, catalytic and proteinase activities; regulation of signaling transduction and protein trafficking, immunity and storage; and their precise function in the development of insecticide resistance in mosquitoes will provide new insights into how genes are interconnected and regulated in resistance.

## Introduction

Mosquitoes are known vectors of parasites and pathogens of both human and animal diseases and their control is an important part of the global strategy to control mosquito-associated diseases [1]. Insecticides are the most important component of this vector-control effort, and pyrethroids such as permethrin are currently the most widely used insecticides for the indoor control of mosquitoes worldwide and the only chemical recommended for the treatment of mosquito nets, the main tool for preventing malaria in Africa [2]. However, the development of resistance to insecticides, especially to pyrethroids, in mosquito vectors has become a global problem [3]–[8]. An improved understanding of the mechanisms governing insecticide resistance is therefore necessary to provide a foundation for studies seeking to identify genetic markers that can be used to monitor and predict the development of resistance and characterize potential new targets for the development of novel insecticides. Resistance has been assumed to be a pre-adaptive phenomenon, in that prior to insecticide exposure rare individuals already exist who carry an altered genome that results in one or more possible mechanisms (factors) allowing survival from the selection pressure of insecticides [9], [10]. In addition, some studies propose that resistance can also be induced by insecticide exposure [11], and overall, the rate of development of resistance in field populations of insects depends upon the levels of genetic variability in a population [12], [13]. Efforts to characterize the genetic variation involved in insecticide resistance have therefore been fundamental in understanding the development of resistance and studying resistance mechanisms, as well as in practical applications such as designing novel strategies to prevent or minimize the spread and evolution of resistance development and the control of insect pests [14].

The mosquito *Culex quinquefasciatus* Say is a primary vector of West Nile virus, St. Louis encephalitis virus, Eastern Equine Encephalitis virus, Japanese Encephalitis virus, Chikungunja virus, *Wucheria bancroftii*, and pathogens that cause lymphatic filariasis [15], [16]. This mosquito species has a global distribution, especially throughout tropical and temperate climates of the world [17], [18]. In Alabama, *Cx. quinquefasciatus* is the predominant mosquito species in urban areas. Current approaches to controlling mosquitoes in the state rely primarily on source reduction and the application of insecticides, primarily pyrethroids and organophosphates, for both larval and adult mosquitoes [5]. One northern Alabama *Culex* strain, HAmCq^G0^ collected from Huntsville, has demonstrated the ability to develop resistance and/or cross-resistance to not only pyrethroids and organophosphates (OPs), but also relatively new insecticides such as fipronil and imidacloprid [5]. The HAmCq^G0^ mosquito strain has been further selected with permethrin for eight generations in the laboratory to produce the HAmCq^G8^ strain, which has a much higher level of resistance to permethrin than the parental strain, HAmCq^G0^ [19]–[21]. In an effort to better understand the genetic variation in resistant mosquitoes and gain valuable insights into the genes involved in the development of permethrin resistance in *Culex* mosquitoes, we chose the most resistant life stage (fourth instar larvae [21]) and conducted a whole transcriptome analysis of the mosquito *Culex quinquefasciatus* following permethrin selection and examined the gene expression profiles between the lower resistant parental strain HAmCq^G0^ and their permethrin-selected high resistant offspring HAmCq^G8^ using Illumina RNA Seq [22].

## Materials and Methods

### Mosquito strains

*Culex quinquefasciatus* strain HAmCq^G0^ is a low insecticide resistant strain with a 10-fold level of resistance to permethrin compared with the laboratory susceptible S-Lab strain [21]. It was originally collected from Huntsville, Alabama in 2002 and established in laboratory without further exposure to insecticides [5]. The HAmCq^G8^ strain is the 8^th^ generation of permethrin-selected HAmCq^G0^ offspring and has a 2,700-fold level of resistance [21]. All mosquitoes were reared at 25±2°C under a photoperiod of 12∶12 (L:D) h. The mosquito was reared strictly under identical rearing conditions for the two mosquito populations to enter into the fourth instar stage at the same time, which was achieved through the controlling of the egg raft collection, egg hatching, and subsequent larval development and sample collection.

### RNA extraction

A total of 200 fourth instar larvae of the HAmCq^G0^ and HAmCq^G8^ mosquito populations were pooled, flash frozen on dry ice and immediately processed for RNA extraction. The fourth instar larvae were selected for the study largely because the 4th instar life stage of these mosquitoes had highest levels of resistance compared with other developmental stages [21]. Thus, using the highly resistant 4^th^ instar larvae as comparison will provide us clearer picture for the differential gene expression and genes perhaps involved in resistance. Total RNA was extracted using the hot acid phenol extraction method [23], after which a total of 30 µg of RNA was treated with DNase I using the DNA-Free kit from Ambion (Austin, TX) to remove any contaminant DNA. Total RNA was re-extracted with two successive acid phenol: chloroform (1∶1) steps followed by a final chloroform extraction to remove any residual phenol. The RNA was then precipitated over ethanol and resuspended in sterile distilled water. After a 1 µg aliquot of RNA had been visually inspected for quality and for DNA contamination on a 1% agarose gel, total RNA was sent for RNA-Seq analysis (Hudson Alpha Institute of Biotechnology [HAIB]).

### RNA library preparation, RNA Seq sequencing, Data analysis, and gene expression processing

RNA quality was assessed using an Agilent 2100 Bioanalyzer (Agilent, Santa Clara, CA) and an Invitrogen Qubit (Invitrogen, Carlsbad, CA). Libraries were then prepared using the Illumina Tru-Seq RNA Sample Prep Kits (Illumina, San Diego, CA) for mRNA-Seq and a 3′ poly A tail selection method. Samples were barcoded and run as one of four samples on a single lane of an Illumina Hi Seq 2000 chip. Samples for the mRNA Seq were run using the PE-50 module (HAIB). Base calling, initial removal of low quality reads, and barcode parsing were conducted by the staff at HAIB. Data were sorted by coordinate using Picardtools (http://picard.sourceforge.net) [24] and checked for mate-pair matching. Paired end reads were then mapped to the *Cx quinquefasciatus* genome from Vectorbase [25] using Tophat [26] with mate pair interval of 200 bases and the gtf basefeatures file. The – no-novel-juncs flag was used in the alignment to suppress the discovery of novel spliceforms in order to estimate gene expression levels based on the Vectorbase annotation of the genes. Read counts were determined using Cufflinks, and the testing of differential expression was estimated using Cuffdiff [27]. Both Cufflinks and Cuffdiff were used because these programs provide a more accurate estimation of the gene expression value by adjusting for transcript fragment biases that occur at the ends of the transcripts and fragments during the library generation protocol [28]. To adjust for the unequal coverage across a gene, Cuffdiff uses a negative binomial distribution [29] and applies a likelihood function to estimate gene expression that reduces bias, increases reproducibility across libraries, and gives better correlated gene expression levels as estimated by qPCR and determines differentially-expressed genes at the α = 0.05 false discovery rate (FDR) [28]. After analysis, only genes with expression values ≥1, as measured in number of fragments mapped for every thousand bases of gene length for every million fragments sequenced (FPKM), were retained for expression comparisons [30].

**Table 1:** Number of paired end reads from the Illumina HiSeq sequencing and the percentage of reads mapped to the *Cx. quinquefasciatus* (strain: Johannesburg) predicted transcriptome.

Mosquito strain	HAmCq^G0^	HAmCq^G8^
Total reads	32540882†	37184673
Additional reads discarded	31509‡	16219
Reads mapped	23008772	30586459

† Total number of FASTQ (DNA sequence with quality scores) reads passing the Illumina quality filter.

‡ Number of reads discarded due to low quality of one or both of the paired end reads.

### Gene expression validation using quantitative real-time PCR (qRT-PCR)

The 4^th^ instar larvae of each mosquito population had their RNA extracted for each experiment using the acidic guanidine thiocyanate-phenol-chloroform method [23]. Total RNA (0.5 µg/sample) from each mosquito sample was reverse-transcribed using SuperScript II reverse transcriptase (Stratagene) in a total volume of 20 μl. The quantity of cDNAs was measured using a spectrophotometer prior to qRT-PCR, which was performed with the SYBR Green master mix Kit and ABI 7500 Real Time PCR system (Applied Biosystems). Each qRT-PCR reaction (15 μl final volume) contained 1x SYBR Green master mix, 1 μl of cDNA, and a specific primer pair designed according to gene sequences (Table S1) at a final concentration of 3–5 μM. All samples, including the no-template negative control, were performed in triplicate. The reaction cycle consisted of an initial UDG glycosylase step at 50°C for 2 min followed by a melting stage at 95°C for 10 min, followed by 40 cycles of 95°C for 15 sec and 60°C for 1 min. Specificity of the PCR reactions was assessed by a melting curve analysis for each PCR reaction using Dissociation Curves software. Relative expression levels for the genes were calculated by the 2^-ΔΔCT^ method using SDS RQ software [31]. The 18S ribosome RNA gene, an endogenous control, was used to normalize the expression of target genes [32], [33]. Preliminary qRT-PCR experiments with the primer pair (Table S1) for the 18S ribosome RNA gene designed according to the sequences of the 18S ribosome RNA gene had revealed that the 18S ribosome RNA gene expression remained constant in of HAmCq^G8^ and HAmCq^G8^ mosquito populations, so the 18S ribosome RNA gene was used for internal normalization in the qRT-PCR assays. Each experiment was repeated three to four times with different preparations of RNA samples. The statistical significance of the gene expressions was calculated using a Student's *t*-test for all 2-sample comparisons and a one-way analysis of variance (ANOVA) for multiple sample comparisons (SAS v9.1 software); a value of *P*≤0.05 was considered statistically significant.

### Annotation, gene grouping, and functional gene enrichment analysis

The genes were annotated for SCOP general and detailed functions using the predicted *Cx. quinquefasciatus* annotation information available at the Superfamily website (version 1.75) supfam.cs.bris.ac.uk/SUPERFAMILY/index.html [34]. Additional gene information for carboxylesterases was taken from the Vectorbase annotation for the Johannesburg strain version 1.1 (www.vectorbase.org) [25]. Gene Ontology is a method of gene annotation that was introduced in 1998 [35]. It is composed of three sets of structured gene ontology terms (GO terms) that have a carefully controlled vocabulary. These three sets represent 1) *Cellular Component*, which describe where the protein product is located at the sub-cellular and macromolecular complex level, 2) *Biological Process*, which denote gene products that are part of, or are themselves, biological processes, and 3) *Molecular Function*, which describe what the gene product does with regard to its function. Each gene may have multiple GO terms within each of the three sets of GO term ontology. Since the vocabulary of GO terms is carefully controlled, the occurrence of a given GO term can be compared between two distinct sets of genes. This allowed us to conduct an enrichment analysis of GO terms in the differentially-expressed gene sets against the entire expressed gene set using the Gene Ontology terms as annotated for the predicted genes in the *Cx. quinquefasciatus* genome using the online tool g: Profiler biit.cs.ut.ee/gprofiler/welcome.cgi [36], [37]. The g: Cocoa tool was used to test for GO term enrichment using a gSCS threshold for the significance threshold and a static background containing only genes with expression values of ≥1. This analysis took all of the GO terms associated with the differentially down- or up-regulated gene sets and determined if a given GO term was statistically over-represented using a hypergeometric distribution to quantify the sampling probability that a given GO term is statistically more abundant in the up- or down-regulated gene set when compared to the abundance of that same GO term among the entire expressed gene set.

## Results

### Illumina RNA Seq data analysis

The maximum numbers of 51 nt paired-end reads that passed Illumina quality filtering were 32,540,882 and 37,184,673 for HAmCq^G0^ and HAmCq^G8^, respectively (Table 1), which is consistent with the data typically obtained in an RNA Seq reaction that is based on an Illumina HiSeq 2000 single lane consisting of eight barcoded samples with a maximum number of reads passing filter of ∼46 million (Illumina, Inc. San Diego, CA). Reads were mapped to the *Cx. quinquefasciatus* genome (version: CpipJ1.2) from Vectorbase (www.vectorbase.org) [25]. Overall, the sequenced fragments mapped to a total of 14,440 genes, with 12,451 of these having a FPKM value of ≥1.0 in both HAmCq^G0^ and HAmCq^G8^, which was used as the minimum value to detect gene expression [30]. All sequence traces and expression values have been submitted to the Gene Expression Omnibus at NCBI, reference accessions GSE33736 http://www.ncbi.nlm.nih.gov/geo/query/acc.cgi?acc=GSE33736 and SRA048095 (http://www.ncbi.nlm.nih.gov/sra/?term=SRA048095).

### Transcriptome profile: SCOP general categories and detailed function categories

**Figure 1:**
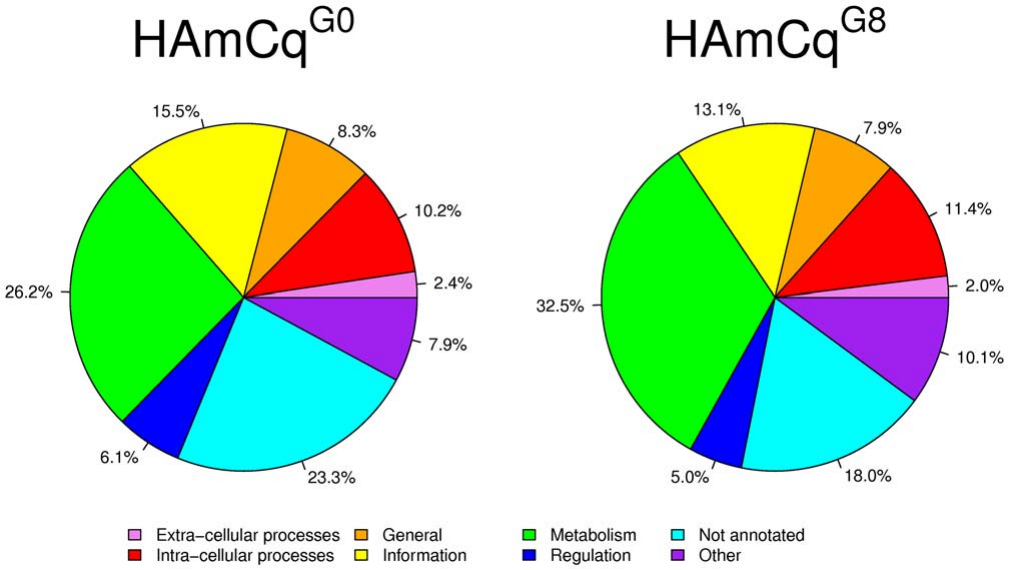
Total proportions of cumulative gene expression levels in HAmCq^G0^ and HAmCq^G8^ for the SCOP general and detailed functions using the predicted *Cx. quinquefasciatus* annotation information available at the Superfamily website (version 1.75) supfam.cs.bris.ac.uk/SUPERFAMILY/index.html.

All expressed genes from both HAmCq^G0^ and HAmCq^G8^ were annotated for protein superfamily using the Structural Classification of Proteins (SCOP) annotations version 1.73 supplied for *Cx. quinquefasciatus* (http://supfam.cs.bris.ac.uk/SUPERFAMILY) [38], classified in terms of eight SCOP general categories, extra−cellular processes, intra−cellular processes, general, information, metabolism, regulation, not annotated, and other/unknown, according to the general function of the proteins. The genes expressed in both HAmCq^G0^ and HAmCq^G8^ were sorted into each of the eight SCOP general categories [39] and then the expression values of each of these genes were summed within SCOP general category to obtain the proportion of total gene expression attributable to each of the SCOP categories (Fig. 1). Overall, the proportions of total gene expression were similar for HAmCq^G0^ and HAmCq^G8^, however there were notable differences between the two mosquito strains for the metabolism category, which accounted for 32% of the gene expression in the entire HAmCq^G8^ genome compared to 26% in HAmCq^G0^, suggesting an up-regulation of genes relating to metabolism in response to permethrin selection. Another difference in the total gene expression was in the not annotated category in HAmCq^G0^, where it accounted for 23% of the gene expression in the entire genome compared to only 18% in HAmCq^G8^, suggesting the down regulation of a set of genes without functional annotation in response to permethrin selection.

### Transcriptome profile: superfamily

**Figure 2:**
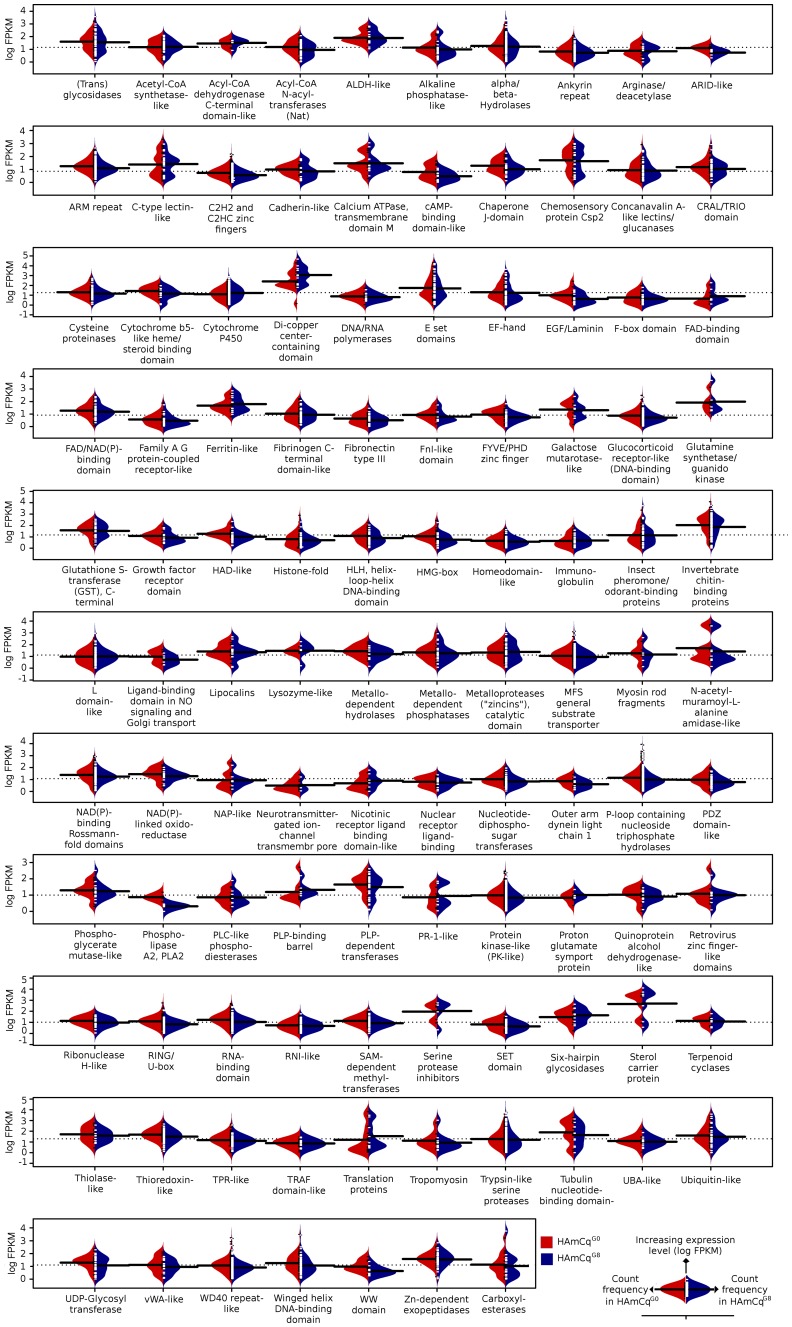
Log normal bean-plots for all expressed genes within SCOP superfamilies (SCOP version 1.75; supfam.cs.bris.ac.uk/SUPERFAMILY/index.html) in HAmCq^G0^ and HAmCq^G8^. The distribution along the Y axis indicates a higher level of gene expression, while the distribution along the X axis indicates the proportion of genes expressed at the given level of gene expression along the Y axis. Distributions are oriented along a common central baseline so that distributions in red (HAmCq^G0^) have more genes expressed at a given gene expression level (log FPKM) if the distribution is further to the left on the X axis, while distributions in blue (HAmCq^G8^) are higher if they are further to the right of the X axis. The central vertical baseline for each superfamily is a mirror point for the two distributions.

Genes were further categorized into protein superfamilies at a gene annotation level lower than detailed function to compare the distribution of the expression levels between HAmCq^G0^ and HAmCq^G8^. This allowed us to evaluate changes in the general gene expression within each of the superfamilies following permethrin selection. A log FPKM transformation was used to normalize the gene expression values and these were then plotted as beanplots (Fig. 2, Table S2). The distribution of each superfamily was broadly classified as unimodal, bimodal, or multimodal (Table S2) according to the similarities of gene expression within that superfamily. In addition, the values of skewness and kurtosis for the gene expression distribution were calculated, representing the symmetry of the gene distributions within the log normal distributions (a positive skewness represents a gene distribution where a majority of genes have low expression levels and a negative skewness one where a majority of the genes have high expression levels) and the degree of sharpness of the curve (in leptokurtic distributions, groups of genes are expressed at similar expression levels and in platykurtic distributions, genes are expressed across a range of expression levels). Overall, all the superfamilies were comparable for HAmCq^G0^ and HAmCq^G8^, both in terms of expression levels and in numbers of genes (as shown along the Y and the X axes, respectively, in Fig. 2, and in Table S2). This suggested that the permethrin selection may not have significantly influenced the overall expression levels of the genes in most superfamilies, However, in some cases the overall gene expression distribution in the two strains did differ slightly in a few superfamilies. For example, the Di-Copper containing center gene superfamily showed a multi modal distribution with three expression peaks in both HAmCq^G8^ and HAmCq^G0^. However, while the magnitudes of all three expression peaks were similar for a number of genes in HAmCq^G8^, the peak with the lower mode of expression was >2-fold higher than the intermediate peak, and more than 5-fold higher than the highest mode in HAmCq^G0^. Similar patterns were also found for the C-type lectin-like, NAP-like, and PLP binding barrel superfamilies. These slight changes in the gene expression distribution pattern may reflect the influence of up-regulated genes on the overall gene expression pattern in each of the superfamilies. The lysozyme-like superfamily, compared with HAmCq^G0^ which had a single mode, contained two modes in HAmCq^G8^ with one distribution positively and the other negatively skewed, suggesting that while some genes in this superfamily were up-regulated in HAmCq^G8^ compared with HAmCq^G0^, the others may be down regulated.

### Transcriptome profile: differential gene expression between HAmCq^G0^ and HAmCq^G8^

**Figure 3:**
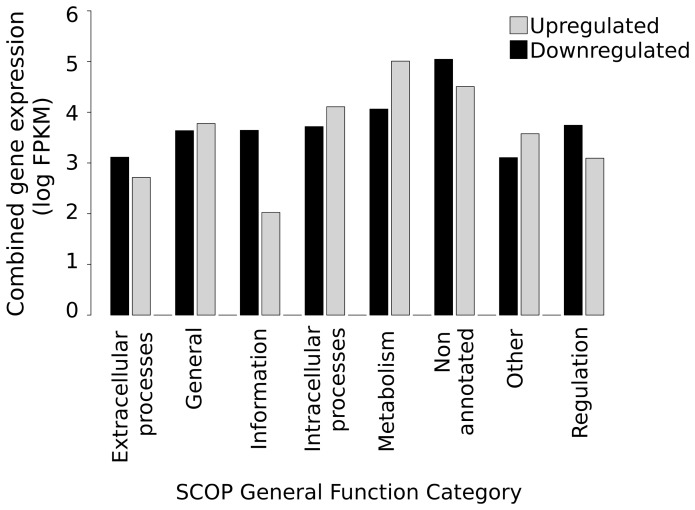
SCOP general function category (SCOP version 1.75; supfam.cs.bris.ac.uk/SUPERFAMILY/index.html) for the total combined gene expression levels (log summed value for all genes within category) for all up- and down-regulated genes within a general function category in HAmCq^G8^ compared to those expressed in HAmCq^G0^.

Looking at the above SCOP general categories, detailed function categories and superfamily categories there is an overall similarity in the pattern of gene expression over the whole transcriptome level between the lower resistance parental mosquito HAmCq^G0^ and their permethrin selected offspring HAmCq^G8^. We therefore went on to characterize the gene expression level between the two mosquito strains using the Cuffdiff algorithm and applying a >2-fold differential expression cut off threshold. A total of 3982 down-regulated and 367 up-regulated genes were identified in HAmCq^G8^ (Table 2, Table S3, Table S4) compared to HAmCq^G0^. Overall, although there were more than 10 times the number of genes down-regulated than up-regulated, the cumulative gene expression values (FPKM) between the down- and up-regulated genes (1.43×10^5^ and 1.53×10^5^, respectively) were similar (Table 3). Interestingly, the predominant SCOP general function category for the down-regulated genes was the non-annotated category (NONA, 2016 genes), which accounted for 50% of the down-regulated genes (Table 2, Fig. 3), and represented 77% of the total cumulative expression (FPKM) of all of the down-regulated genes. This result is consistent with the results for the SCOP general categories, where a decrease in the total gene expression was found in the NONA category for HAmCq^G8^ compared to HAmCq^G0^. In contrast, only 17% of the cumulative expression of the up-regulated genes in HAmCq^G8^ was in the NONA category. Nevertheless, the highest cumulative gene expression of up-regulated genes was in the metabolism general function category (Table 2, Fig. 3), which accounted for 67% (FPKM) of all of the up-regulated gene expression, while the cumulative expression of this category accounted for only 8% of the total cumulative expression of the down-regulated genes. Taken together, these results not only reveal equally dynamic changes in abundance for both the increases and decreases in the total gene expression for different categories in *Cx. quinquefasciatus* following permethrin selection, but also indicate an important feature of metabolic gene up-regulation in response to insecticide resistance and permethrin selection that is consistent with the data from the SCOP general category analysis, where the total gene expression in the metabolism SCOP general category was found to be higher in HAmCq^G8^ than in HAmCq^G0^.

**Table 2:** Numbers of differentially-expressed genes and their cumulative gene expression level in HAmCq^G8^ sorted by the Structural Classification Of Proteins general function category.

	Down-regulated†		Up-regulated	
SCOP‡ general function category	#genes	FPKM* range (cumulative)	#genes	FPKM range (cumulative)
Extra-cellular processes	101	0.1–126.4 (1.29×10^3^)	20	1.0–121.8 (5.42×10^2^)
General	355	0.1–192.1 (4.31×10^3^)	39	1.0–1750.5 (5.99×10^4^)
Information	171	0.1–344.9 (3.68×10^3^)	5	4.3–66.0 (1.05×10^2^)
Intra-cellular processes	359	0.1–255.4 (5.18×10^3^)	58	1.7–5763.6 (1.28×10^4^)
Metabolism	397	0.1–737.5 (1.15×10^4^)	91	1.1–47162.3 (1.02×10^5^)
NONA§	2016	0.0–33037.0 (1.10×10^5^)	125	1.0–5766.3 (2.62×10^4^)
Other	57	0.6–167.1 (1.26×10^3^)	5	7.5–1742.1 (3.76×10^3^)
Regulation	526	0.0–584.3 (5.56×10^3^)	24	1.5–865.9 (1.25×10_3_)
TOTAL	3982	(1.43×10^5^)	367	(1.53×10^5^)

† Down-regulated/Up-regulated genes represent those genes that differed in their expression level (FPKM) in HAmCq^G8^ by more than two fold when compared to the parental strain HAmCq^G0^.

‡ SCOP general function categories annotated using the predicted *Cx. quinquefasciatus* annotation information available at the Superfamily website (version 1.75) supfam.cs.bris.ac.uk/SUPERFAMILY/index.html.

* Fragments mapped Per Kilo bases of reference sequence for every Million fragments sequenced.

§ NONA: Not annotated.

**Table 3:** Gene Ontology (GO) term enrichment analysis results for differentially expressed genes in HAmCq^G8^.

GO level	GO term†	Term domain and name	# hits	p-value‡
**Down-regulated genes**				
**Molecular function**			-	-
structural molecule activity (GO:0005198)			163	1.08×10^−11^
structural constituent of cuticle (GO:0042302)			85	3.19×10^−18^
**Up-regulated genes**				
**Biological process (GO:0008150)**			**193**	**4.28×10^−10^**
metabolic process (GO:0008152)			139	1.02×10^−7^
oxidation-reduction process (GO:0055114)			38	5.39×10^−9^
proteolysis (GO:0006508)			55	2.35×10^−16^
**Molecular function (GO:0003674)**			**250**	**9.80×10^−6^**
Catalytic activity (GO:0003824)			162	2.09×10^−13^
oxidoreductase activity (GO:0016491)			47	1.05×10^−10^
monooxygenase activity (GO:0004497)			29	2.84×10^−15^
hydrolase activity (GO:0016787)			90	1.29×10^−12^
peptidase activity (GO:0008233)			54	2.16×10^−14^
peptidase activity, acting on L-amino acid peptides (GO:0070011)			52	1.99×10^−15^
exopeptidase activity (GO:0008238)			10	4.66×10^−5^
carboxypeptidase activity (GO:0004180)			7	1.03×10^−4^
endopeptidase activity (GO:0004175)			41	1.47×10^−12^
metallopeptidase activity (GO:0008237)			20	6.26×10^−10^
metalloendopeptidase activity (GO:0004222)			11	2.42×10^−6^
serine hydrolase activity (GO:0017171)			30	4.39×10^−9^
serine-type peptidase activity (GO:0008236)			30	4.39×10^−9^
serine-type endopeptidase activity (GO:0004252)			28	1.89×10^−8^
hydrolase activity, acting on glycosyl bonds (GO:0016798)			15	1.23×10^−7^
hydrolase activity, hydrolyzing O-glycosyl compounds (GO:0004553)			13	1.17×10^−6^
Electron carrier activity (GO:0009055)			28	6.65×10^−14^
Binding activity			-	-
tetrapyrrole binding (GO:0046906)			32	1.55×10^−17^
iron ion binding (GO:0005506)			33	3.19×10^−15^
heme binding (GO:0020037)			32	1.32×10^−17^

† Annotation from the Gene Ontology consortium (version 1.2084; release date: 12:07:2011).

‡ Cumulative hypergeometric p-values for GO terms of genes that were differentially up-regulated in when tested against all genes with expression levels of FPKM >1 using the g: SCS threshold.

GO Terms that do not have values for the number of hits or p-values were not statistically enriched in the functional enrichment analysis, but are included in the table to provide all parenthood connections.

### Functional enrichment analysis of GO terms for differentially expressed genes

To interpret the gene expression data and gain more insight into the biological mechanisms driving the up- and down-regulated genes, Gene Ontology (GO) term enrichment or functional enrichment analysis [36], [37], [40] was performed to identify significantly enriched GO terms among the up- and down regulated genes in HAmCq^G8^. GO terms are groups of genes sharing common biological function, regulation, or interaction (http://biit.cs.ut.ee/gprofiler/gconvert.cgi). A statistical analysis reveals which GO terms are over-represented and have hence been “enriched”, or are more prevalent, within the down- or up-regulated genes in HAmCq^G8^. Each gene can have multiple GO terms and these are part of a carefully-controlled vocabulary that allows for genes of various annotations to be grouped according to common attributes such as their cellular components, biological processes, or molecular functions [35]. Overall, the functional enrichment analysis showed that among the down-regulated gene set in HAmCq^G8^, the terms GO:000581 (structural molecule activity) and GO:0042302 (structural constituent of cuticle) were the only statistically over represented GO terms (*P* = 1.08×10^−11^ and 3.19×10^−18^, respectively) (Table 3). For the 3982 down-regulated genes in HAmCq^G8^, there were 85 hits for GO:000581 and 163 hits for GO:0042302, indicating that 85 of the 3982 down-regulated genes had the structural molecule activity function and 163 the structural constituent of cuticle GO terms. Since these were the only enriched molecular function GO terms among the down-regulated gene set, there are likely to be changes of gene expression in the structural component of the cuticle in the HAmCq^G8^ mosquitoes compared to the parental HAmCq^G0^ strain.

The functional enrichment analysis of the 367 up-regulated genes in HAmCq^G8^ identified 25 statistically enriched GO terms (Table 3), four of which were in the categories biological process (CO:0008150), metabolic process (CO:0008152), proteolysis (GO:0006508), and oxidation-reduction process (GO:0055114). Among these four enriched GO terms, biological process (CO:0008150) and metabolic process (CO;0008152) were the predominant GO terms, with 193 and 139 hits, respectively, suggesting that the major up-regulated genes were involved in biological and metabolic processes. The remaining 21 statistically enriched GO terms were in the molecular function category (Table 3) and the GO terms for catalytic activity (GO:0003824), hydrolase activity (GO:0016787), peptidase activity (GO:0008233), peptidase activity acting on L-amino acid peptides (GO:0070011), and oxidoreductase activity (GO:0016491) were the predominant GO terms, with hits that ranged from 162 to 47. Comparing the statistically enriched GO terms between the up- and down-regulated genes, these two sets of genes had obvious differences in their functions: the down-regulated genes primarily represented structural or cuticular structural activity functions, while the up-regulated genes were predominantly related to catalytic, metabolic, and proteolytic activity.

### The molecular functional parenthood relationships of the GO terms among up-regulated genes and their interconnection

**Figure 4:**
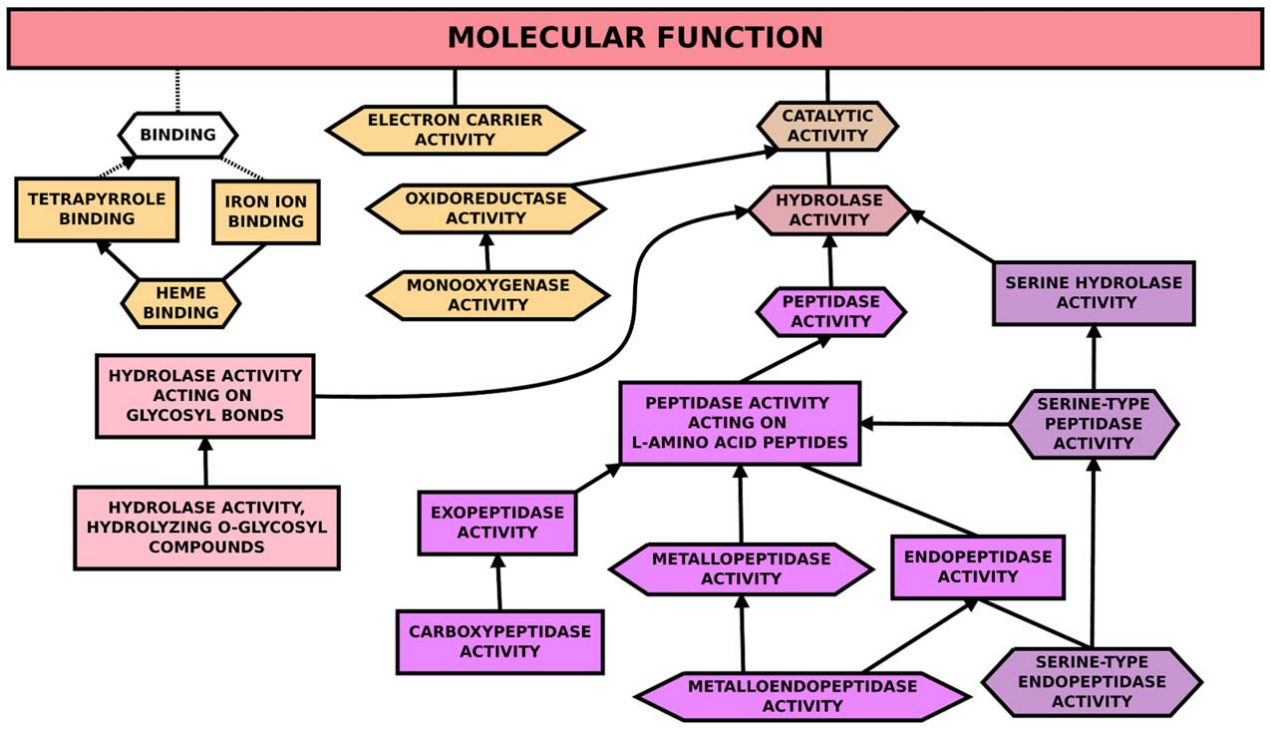
Parent-Child association for functionally enriched Gene Ontology (GO) terms among genes that were up-regulated in HAmCq^G8^. GO terms (GO consortium, version 1.2084; release date: 12:07:2011). GO terms associated with the up-regulated genes in HAmCq^G8^ were considered statistically enriched if the cumulative hypergeometric p-value was <0.001 using the g: SCS threshold in g: Cocoa (http://biit.cs.ut.ee/gprofiler/gcocoa.cgi). Colored boxes represent statistically functionally enriched GO terms, while the nonsignificantly-enriched GO term is marked in white and provided to display all of the parent-child relationships in the network. Lines and/or arrows represent connections between or among different GO terms. Solid lines represent relationships between two enriched GO terms. Dashed lines represent relationships between enriched and unenriched terms or between unenriched GO terms, and are provided to connect all of the nodes on the directed acyclic graph to the main molecular function category.

The relationships among the GO terms in the molecular function category were investigated in the up-regulated genes in HAmCq^G8^ by determining whether their connection was a part of the same process or whether a parenthood process was involved [35]. Overall, 3 functional sets of GO terms were found to be significantly overrepresented among the GO terms for molecular function (Fig. 4, Table 3), namely electron carrier activity, binding, and catalytic activity. The electron carrier activity set was mainly associated with GO terms in cytochrome P450 genes (Table S5). The category for binding had three child branch nodes, all of which were related to metal binding: tetrapyrrole binding, iron binding, and heme binding (Fig. 4). These child branch nodes were again associated with the GO terms that were mainly overrepresented among cytochrome P450 genes (Table S5). The next major category was catalytic activity, which had two main child branch nodes: oxidoreductase activity with an additional branch node for monooxygenase activity, both of which had their GO terms present in the genes annotated as cytochrome P450s (Table S5); and hydrolase activity, which contained three additional branch nodes. Of these additional hydrolase branch nodes, the first was for hydrolase activity of glycosyl bonds, with an additional sub-branch node for hydrolyzing O-glycosyl compounds. This was significantly overrepresented among the enzymes corresponding to the function of hydrolyzing glycosyl compounds such as alpha-L-fucosidases, alpha amylases and alpha glucosidases. The other two additional hydrolase branch nodes were peptidase/proteinase activity, which had an additional six sub-branch nodes relating to different peptidase/proteinase activities, and serine hydrolase activity, which had two additional sub-branch nodes for serine-type peptidase activity and serine-type endopeptidase activity (Fig. 4). The peptidase/proteinase and serine hydrolase activity nodes interconnected through the GO term nodes of endopeptidase activity and peptidase activity acting on L-amino acid peptides, suggesting that the GO terms associated with proteinase activity among the differentially up-regulated gene set in HAmCq^G8^ were interconnected. Therefore, investigating the relationships among these enriched GO term categories of up-regulated genes revealed that functional categories were mainly overrepresented among P450s and proteases/serine proteases.

Indeed, the up-regulation of gene expression in these two categories was further confirmed by validation study of gene expression using qRT-PCR. Overall, the qRT-PCR validation data was consistent with the RNAseq data, showing a general trend of differential expression of genes between HAmCq^G8^ and HAmCq^G0^. A total of 14 up-regulated P450 genes and 24 protease related genes, which showed ≥2-fold higher expression in HAmCq^G8^ compared with HAmCq^G8^ in the RNAseq data, were selected for the study (Table 4). All 14 cytochrome P450s were up-regulated by at least 2-fold in the HAmCq^G8^ strain compared with HAmCq^G0^, which was consistent with the data generated using the RNAseq. Among the 23 up-regulated proteinase genes that have been identified by RNAseq, 14 of them (60%) were up-regulated by at least 2-fold in the HAmCq^G8^ strain and nine were up-regulated with a range of 1.5- to 1.8-fold compared with HAmCq^G0^ (Table 4). However, one of the proteinase genes had an expression level of 1.1-fold in HAmCq^G8^ compared with HAmCq^G0^, which was significantly different from the RNAseq data.

**Table 4:** qRT-PCR validation of selected up-regulated genes in HAmCq^G8^ as identified by the RNASeq quantification.

			The ratio of gene overexpression in HAmCq^G8^ †	
Gene category	Vectorbase #	Annotation§	RNASeq	qPCR
Cytochrome P450	CPIJ002538	CYP6AG12‡	3.7	2.1††
	CPIJ005959	CYP6AA7‡	7.3	2.1
	CPIJ005957	CYP6AA9‡	6.6	2.8
	CPIJ010546	CYP9J34‡	13.4	2.9
	CPIJ009478	CYP4D42v1‡	2.4	3.2
	CPIJ005956	CYP6BZ2‡	3.3	3.7
	CPIJ010537	CYP9J45‡	4.8	3.8
	CPIJ012470	CYP9AL1‡	9.2	3.8
	CPIJ014218	CYP9M10‡	3.7	4.2
	CPIJ010225	CPY12F7‡	3.9	5.2
	CPIJ010227	CYP12F13‡	7.1	5.2
	CPIJ010543	CYP9J40‡	7.2	6.0
	CPIJ005955	CYP6P14‡	8.2	6.3
	CPIJ020229	CYP4D42v2‡	2.4	7.0
Protease	CPIJ002139	HzC4 chymotrypsinogen	4.3	1.1±0.11
	CPIJ002130	kallikrein-7	2.4	1.5±0.50
	CPIJ013319	metalloproteinase	3.5	1.5±0.10
	CPIJ009106	angiotensin-converting enzyme	2.7	1.5±0.84
	CPIJ001240	cathepsin B-like thiol protease	5.3	1.6±0.46
	CPIJ019428	trypsin 2	3.4	1.6±0.04
	CPIJ004086	angiotensin-converting enzyme	5.7	1.7±0.62
	CPIJ008873	prolylcarboxypeptidase	3.5	1.7±1.09
	CPIJ002135	trypsin alpha-4	5.9	1.8±0.84
	CPIJ016012	tryptase-2	2.2	1.8±0.10
	CPIJ002142	chymotrypsin BI	2.8	2.0±0.42
	CPIJ006803	zinc metalloproteinase nas-7	4.5	2.0±0.21
	CPIJ007383	endothelin-converting enzyme 1	2.5	2.1±1.08
	CPIJ010224	metalloproteinase	2.9	2.4±0.74
	CPIJ014523	elastase-3A	3.0	2.4±0.71
	CPIJ019029	metalloproteinase	2.6	3.6±0.70
	CPIJ002128	mast cell protease 2	16.1	3.6±0.04
	CPIJ006542	chymotrypsin-2	19.7	5.4±1.84
	CPIJ010805	carboxypeptidase A1	4.4	6.9±3.72
	CPIJ006076	hypodermin-B	17.0	11.6±4.96
	CPIJ001743	carboxypeptidase A2	5.4	16.3±5.48
	CPIJ003623	coagulation factor XII	7.1	54.2±19.79
	CPIJ001742	zinc carboxypeptidase	3.0	99.5±19.35
	CPIJ009594	nephrosin	21.7	144.3±13.8

§ *Culex quinquefasciatus* genome, Johannesburg strain CpipJ1.2, June 2008; http://cquinquefasciatus.vectorbase.org/.

† The ratio of gene overexpression in HAmCq^G8^ compared to HAmCq^G0^.

‡ Annotations for cytochrome P450 genes were taken from the most current annotation based on: Nelson (2009) The Cytochrome P450 Homepage. Human Genomics 4, 59-65: http://drnelson.uthsc.edu/CytochromeP450.html.

†† Data reprinted from Yang and Liu, 2011.

## Discussion

Based on the findings of our previous research, which has included synergism studies on the inhibition of metabolic enzymes [6], studies on the target site insensitivity of sodium channels in permethrin resistance [19], gene expression profiles of resistance from a resistant-susceptible mosquito subtractive library [41], research into the genetic inheritance of permethrin resistance [21], and, most recently, studies of the gene expression and characterization of P450 genes covering the entire genome sequence of resistant mosquitoes [32], [33], it seems clear that a multiple mechanism/gene-interaction phenomenon is responsible for the development of permethrin resistance in *Culex* mosquitoes. We consider it very likely that normal biological and physiological pathways and gene expression signatures are altered in the resistant mosquitoes through changes in multiple gene expression in the resistant mosquitoes following insecticide selection that allow them to adapt to environmental or insecticide stress. While a great deal of effort has been devoted to identifying and characterizing the mechanisms and genes involved in insecticide resistance, and significant progress has been made, our previous approaches to characterizing the individual genes associated with insecticide resistance have not yet resulted in a global understanding of the complex processes responsible for resistance. The recent genome sequencing of *Cx. quinquefasciatus* [16] has made direct comparisons of gene expression at the whole genome level between samples possible. The whole transcriptome analysis of the mosquito *Culex quinquefasciatus* following permethrin selection using Illumina RNA Seq reported here has allowed us to compare the cumulative gene expression in HAmCq^G0^ and HAmCq^G8^ mosquitoes in the SCOP general function categories and superfamilies, enabling us to evaluate major changes in the gene expression within each of the categories in the mosquitoes following permethrin selection using their median expression values. In general, similar levels of total cumulative gene expression were identified in the HAmCq^G0^ and HAmCq^G8^ mosquitoes in each of the general function categories, suggesting that the permethrin selection may not change the majority of the gene expression occurring in the mosquito genome, but that the changes that are found in only a select number of genes should be correlated to the permethrin selection process undergone by HAmCq^G8^.

Results from our previous studies [5], [6], [13], [19], [41] and from many others [42]–[47] suggest that the interaction of multiple insecticide resistance mechanisms or genes may be responsible for insecticide resistance. While it is unclear whether and how these up-regulated genes are associated with insecticide resistance, the findings reported in these papers suggest that insecticide resistance in mosquitoes involves both multiple gene up-regulation and multiple complex interaction mechanisms. Taken together, the above findings suggest that not only is insecticide resistance conferred via multi-resistance mechanisms or up-regulated genes, but it is mediated through the interaction of resistance genes. The current study identified a total of 367 and 3982 genes that were up- and down-regulated, respectively, in permethrin selected offspring HAmCq^G8^ compared with the parental HAmCq^G0^ strain. These results provide further evidence to confirm our hypothesis that multiple gene expression in resistant mosquitoes changes following insecticide selection, thus allowing them to adapt to environmental or insecticide stress. Further, when we validated our RNAseq data using qPCR, we were able to confirm that all of the cytochrome P450 genes identified as upregulated along with 60% of the proteases were indeed upregulated. Previous work using human colorectal cell lines showed that among 192 human exons , 88% of those identified as overexpressed using RNASeq could be validated as having either higher or lower expression using qPCR, although the fold expression between the two strains was variable [48]. This suggested that the RNAseq methodology was suitable for the identification of genes putatively involved in insecticide resistance based on gene expression level, although some genes of interest may be overlooked due to differences in gene sequence, or genes involved in cell signaling that do not need to be more than two-fold expressed in order to be of importance to insecticide resistance.

To interpret the gene expression data and gain fresh insights into the biological mechanisms affected by the up- and down-regulated genes/proteins, we characterized the GO term enrichment, or functional enrichment, by identifying the significantly enriched GO terms among the up- and down-regulated genes in the low resistance parental strain and the high resistance eighth generation offspring. As described earlier, three categories of GO terms are used to describe gene products: biological processes, molecular functions, and cellular components [35]. This approach facilitates efforts to understand the functional relevance of genes, allowing genes or family members that share functional and structural properties to be studied as a whole. Our comparison of the enriched GO terms in the up- and down-regulated genes in HAmCq^G8^ revealed that the two enriched GO terms for the down-regulated genes represented primarily structural or cuticular structural functions and 50% of all the down-regulated genes, representing 77% of the total cumulative expression of those genes, were non-annotated. In contrast, the enriched GO terms for the up-regulated genes represented mainly the catalytic, metabolic, and proteolytic functions, and only 17% of the cumulative expression of the up-regulated genes was in the NONA category. Nevertheless, from an overall cumulative gene expression point of view, we saw similar expression levels between the up- and down-regulated genes in permethrin selected HAmCq^G8^. Taken together, these results not only revealed different patterns in the enriched GO terms/functions for both the up- and down regulated genes, but also equally the dynamic changes in the abundance of both the total increased and the total decreased gene expression in *Culex* mosquitoes following permethrin selection. A number of mechanisms have been proposed for the balancing of up- and down-regulation, including: 1) an adaptive homeostatic response that protects the cell from the deleterious effects of oxidizing species, nitric oxide, or arachidonic acid metabolites from catalytic and/or metabolic enzymes [49], [50]; 2) a homeostatic or pathological response to inflammatory processes [51]; and/or 3) a need for the tissue to utilize its transcriptional machinery and energy for the synthesis of other components involved in the inflammatory response [52]. Whether the up- and down regulated genes identified in the resistant *Culex* mosquitoes by our study reflects a homeostatic response of mosquitoes to insecticides needs to be further studied.

The functional relationships among the enriched GO terms of up-regulated genes/proteins allowed us to identify the key components involved in insecticide resistance and gain an insight into the molecular mechanisms in resistant mosquitoes as a whole. Three molecular function categories, namely electron carrier activity, binding, and catalytic activity, were significantly overrepresented among the GO terms for the up-regulated genes. Investigating the relationships among these enriched GO term categories revealed that functional categories were mainly overrepresented among P450s and proteases/serine proteases. Among these two key components, the importance of P450s has been extensively studied and it has been demonstrated that basal and up-regulation of P450 gene expression can significantly affect the disposition of xenobiotics or endogenous compounds in the tissues of organisms, thus altering their pharmacological and/or toxicological effects [53]. In many cases, increased P450-mediated detoxification has been found to be associated with enhanced metabolic detoxification of insecticides, as evidenced by the increased levels of P450 proteins and P450 activity that result from constitutive overexpression of P450 genes in insecticide resistant insects [33], [54]–[62]. In addition, multiple P450 genes have been identified as being up-regulated in several individual resistant organisms, including house flies and mosquitoes [32], [33], [46], [58], [59], [63], thus increasing the overall expression levels of P450 genes. Our recent studies on the characterization of P450s, their expression profiles, and their important role in the response to insecticide treatment found that multiple P450 genes were up-regulated in resistant and permethrin selected *Cx quinquefasciatus* [32], [33]. These findings together strongly suggest that overexpression of multiple P450 genes is likely to be a key factor governing the increased levels of detoxification of insecticides and insecticide resistance.

In contrast to the well-known role of P450s in insecticide resistance, apart from a few examples, less is known about the function of proteases/serine proteases in resistance. Proteases are a potent class of enzymes that catalyze the hydrolysis of peptide bonds and are known to be involved in a wide range of physiological functions, including the digestion of dietary protein, blood coagulation, immune response, hormone activation, and development [64]. In addition to their digestive, catalytic, proteinase activities, proteases/serine proteases are involved in the regulation of signaling transduction [65]–[68] and cellular protein trafficking in eukaryotic cells [69]. Indeed, the up-regulation of protease genes have been identified in in DDT resistant *An. gambiae* [47], fenitrothion resistant house flies, *Musca domestica* [70], [71], as well as DDT resistant *D. melanogaster* [72]. It has been suggested that the up-regulation of proteases may enable insects to rapidly degrade proteins for their re-synthesis into detoxification enzymes as has been postulated for *M. domestica* when challenged with the insecticide fenitrothion [70]. In addition, two serine protease genes from *Cx. pipiens pallens* have been found to be up-regulated in a deltamethrin-resistant strain [73]. These reports, together with the findings reported here, suggest the importance of the up-regulation of proteases in insecticide resistance. Whether the up-regulated proteases identified in the resistant mosquitoes play a role in the degradation of proteins for biosynthesis of the up-regulated metabolic proteins, particularly P450s and the other proteins involved in the regulation of insecticide resistance, or whether there is some form of interaction with the up-regulated genes associated with signaling transduction and protein trafficking needs further investigation.

In conclusion, this study not only provides a catalog of genes that were co-up- and down-regulated and information about their potential functions, but may also ultimately lead to a deeper understanding of transcriptional regulation and the interconnection of co-regulated genes, including metabolic genes, genes with catalytic activities, genes with proteolytic activities, and genes with, perhaps, functions involved in the regulation, signaling transduction, and protection of cells and tissues in resistant mosquitoes. It has been suggested that co-overexpressed genes are frequently co-regulated [74], [75]. Therefore, characterizing these co-regulated genes as a whole will represent a good starting point for characterizing the transcriptional regulatory network and pathways in insecticide resistance, improving our understanding of the dynamic, interconnected network of genes and their products that are responsible for processing environmental input, for example the response to insecticide pressure, and the regulation of the phenotypic output, in this case, the insecticide resistance of insects [75]. The new information presented here will provide fundamental new insights into precisely how insecticide resistance is regulated and how the genes involved are interconnected and regulated in resistance.

## Supporting Information

Table S1 **List and sequences of the qRT-PCR primers used.**
(DOC)

Table S2 **Lognormal distributions for expressed genes in HAmCq^G0^ and HAmCq^G8^ by superfamily.**
(DOC)

Table S3 **Complete list of all differentially upregulated genes^†^ in HAmCq^G8^.**
(DOC)

Table S4 **List of genes downregulated by at least two-fold in HAmCq^G8^ when compared to HAmCq^G0^.**
(DOC)

Table S5 **List of differentially upregulated genes in HAmCq^G8^ which contained functionally-enriched Gene Ontology terms.**
(DOC)
