# Supplementary material for: The Transcriptome Profile of the Mosquito Culex quinquefasciatus following Permethrin Selection
Source: PLoS One. 2012 Oct 5;7(10):e47163. doi: 10.1371/journal.pone.0047163 (PMC3465273; doi:10.1371/journal.pone.0047163)
Supplement: Table S1 — List and sequences of the qRT-PCR primers used. (DOC) [file pone.0047163.s001.doc]

Table S1. List and sequences of the qRT-PCR primers used.

| Gene^*^ | Sense primer (5' to 3') | Antisense primer (5' to 3') |
| --- | --- | --- |
| 18S rRNA | CGCGGTAATTCCAGCTCCACTA | GCATCAAGCGCCACCATATAGG |
| CPIJ002802 | CTGCATGAAGCTCCGTTGT | AGGTGCTCTCGTGGGTAGC |
| CPIJ002795 | ACGCTCCAGCTCTGTCGTA | GTGTAGCTGGTGGCGTGAG |
| CPIJ011785 | AGCTGGTGTTCCAGGTGTTC | AGCTTTTGTTGGGGATTGTG |
| CPIJ002943 | ATGAGTAACGAGTTCCAGGAGTTG | TTCTCGAACAAAATATCGACAAAC |
| CPIJ001979 | CGAGTCTACCTACACTGGGAAGAT | TAATTCCAGCTTGATGGTTCACTA |
| CPIJ014110 | CAACGGTACTACATCTTTCACCAA | ATGTTTGTTTCCAAATGGGTACTT |
| CPIJ004594 | GGATTTCGGTAAATTTGAAGATGT | TTTTCGTACGTCATTTTAAACAGC |
| CPIJ014719 | TTCAAGGGAACCTGGAAGC | ACGCGTTGAGCTCTTCAAA |
| CPIJ004323 | AAAAGTTCCGACCGGTGAC | CGAGGTGTGTCCCATTGAC |
| CPIJ002139 | TAATCTGTCGTGTCAATTGTCGTA | GGAAGCTATGTATTCCGATGAGAT |
| CPIJ002942 | GACTATGGCAGTGTGATGCACTA | CAAGCACCCATACATCAAGTTTAG |
| CPIJ001111 | ACTGGTTATTCAGCGGTGTACTTT | TTTGATCCAAGGAAGATACGTTTT |
| CPIJ018037 | GGATGTGGTCAGACTGGGTAGTAT | AAACGCTACATCTCTCTCCAACTT |
| CPIJ006543 | AGGTTGATGAGGAGGAGAATACAG | GGATAGATATGCTCATCGTGGAAC |
| CPIJ002130 | GTGGAGGTTCTAAAATTTCCAGTG | TCAGGACAAACGTAGACGAAATTA |
| CPIJ013319 | CTACTACGGTAGCGTGATGCACTA | ACATGTAGTTGACTGCAAGGATGT |
| CPIJ009106 | CAAACCAGAAGAGTACAACTGTCG | GTAGGACACAAAGTAGCGCAGATA |
| CPIJ001240 | AGGACGTGAATATCGTTCTGAAAT | GTTCTGATAGATCTCGGCTTTCAT |
| CPIJ019428 | GGTTCAATAATATCCGAACGATG | GGATGCACGAAGATACTACGAAC |
| CPIJ004086 | TTGAGACGTACAATCAGCAATTTT | TTGTTGTAATTCTCCTGCACAAAT |
| CPIJ008873 | CAGTGAGCTGTTCAATACCTGTTC | GAGTTTGACAGAGCCTATCGGTAT |
| CPIJ002135 | TTCAACGACTATGTTCAACCAATC | CGTATACCTCACCATGTCAGATTC |
| CPIJ016012 | GGGAGTTATGTTGAGGACTTGAAA | GAAGGGTGGCACAGTTATTTATTC |
| CPIJ002142 | TGAAATCCTTAGTAGTGCTTGCAG | TGACCAGAGAGAAGGATGTTGATA |
| CPIJ006803 | GGTAATCTGGTGGAGAGTGACAT | AATGAAGTGACGTTCGGTTTTATT |
| CPIJ007383 | TTTGGTTGACATTGAAAACACTCT | AGCTCTCGTTTCATCTTCTTGATT |
| CPIJ010224 | GAACTATTCGAGACCGGTAGTGAT | GTGAAATTTGCTCCTCAAACACTT |
| CPIJ014523 | TTTTAACGATTACGTTCAACCTGT | AATCTCTGGCGCCATAATAGTAAC |
| CPIJ019029 | CTACACCAACAACAAGTGGAACA | GGTTCACGTAGATGTACTCGTCAC |
| CPIJ002128 | TTGTTATTCTTCTCACAGCAGCTC | CATAGTCATACCATTGGTCCAGTC |
| CPIJ006542 | TGTCGACGAATCAGTTCACTTTAT | CACTTCAAAGTGGGTAGCTGAAC |
| CPIJ010805 | ATTGGAACTACTCATGAGGGAAGA | AGATGCATAATGGTCATAATGGTG |
| CPIJ006076 | CTGAAAACAACACAACCTATGGTC | ATTCTCGGAAACCTCTCCACTAAC |
| CPIJ001743 | GCCTTTGGATGGACTGACTACTAC | CTTGTGGGATAGTTTTACCACCTT |
| CPIJ003623 | CAGTCGAGTAAACATCACCGATAG | GACCAAATGAAGTTATGCCGTACT |
| CPIJ001742 | TGATTTTGAGGAACTTACAACGAA | AGATTTCAGCCGTGGAGTAGTAGT |
| CPIJ009594 | GAAGTATCAGACAACCGCATTCTA | TTTCAAGTTGTTCATCACTGGTCT |
| CPIJ018233 | GTCTGCTTGGGTTCTTCAGC | CGTCACATTGTTCGGATCAC |
| CPIJ006166 | AAGGGAACGTCGGATGAAG | CCTTGTCCATCAGCCAGAA |
| CPIJ001820 | GTTGAATTCTACAAGCACGGTATG | CGTAGTAGAAAACGTGGAACAGAG |
| CPIJ000056 | GAGCTACCTGCCATACTACACCTT | GAAGAAGTCAAAGTACGTGAGCAG |
| CPIJ009033 | ATCGACTTCAGCTATTTCTTCACC | GTCGGTAGTGTTTAGTACGACGTG |
| CPIJ009032 | AGTTGAGATCAAGGAGTTTTCCAG | GGGAGTTCTTGTAGTTGAAGGGTA |
| CPIJ007783 | ACTACCAATTCAAGGATCACCTTC | AGTATGTGACCAACTTGTCAATGG |

^*^*Culex quinquefasciatus* genome, Johannesburg strain CpipJ1.2, June 2008; http://cquinquefasciatus.vectorbase.org/
