# Supplementary material for: The Transcriptome Profile of the Mosquito Culex quinquefasciatus following Permethrin Selection
Source: PLoS One. 2012 Oct 5;7(10):e47163. doi: 10.1371/journal.pone.0047163 (PMC3465273; doi:10.1371/journal.pone.0047163)
Supplement: Table S2 — Lognormal distributions for expressed genes in HAmCqG0 and HAmCqG8 by superfamily. (DOC) [file pone.0047163.s002.doc]

Table S2. Lognormal distributions for expressed genes in HAmCqG0 and HAmCqG8 by superfamily.

| **Superfamily^†^** | **Strain** | **N^‡^** | **median** | **mean** | **modality** | **kurtosis** | **skewness** |
| --- | --- | --- | --- | --- | --- | --- | --- |
| (Trans)glycosidases | HAmCq^G0^ | 46 | 1.64 | 1.60 | bimodal | -0.51 | 0.14 |
|  | HAmCq^G8^ | 45 | 1.53 | 1.55 | bimodal | -0.42 | 0.29 |
| Acetyl-CoA synthetase-like | HAmCq^G0^ | 38 | 1.17 | 1.17 | unimodal^*^ | -0.86 | -0.31 |
|  | HAmCq^G8^ | 34 | 1.16 | 1.19 | unimodal | -0.50 | -0.17 |
| Acyl-CoA dehydrogenase C- | HAmCq^G0^ | 5 | 1.54 | 1.46 | unimodal^*^ | -2.96 | -0.32 |
| terminal domain-like | HAmCq^G8^ | 5 | 1.67 | 1.50 | unimodal | 4.06 | -2.01 |
| Acyl-CoA N-acyltransferases | HAmCq^G0^ | 41 | 1.17 | 1.18 | unimodal^*^ | -0.70 | -0.05 |
|  | HAmCq^G8^ | 43 | 1.04 | 0.95 | unimodal^*^ | -1.04 | 0.02 |
| ALDH-like | HAmCq^G0^ | 12 | 1.81 | 1.90 | bimodal | -0.17 | 0.70 |
|  | HAmCq^G8^ | 14 | 1.82 | 1.85 | unimodal^*^ | -0.71 | 0.29 |
| Alkaline phosphatase-like | HAmCq^G0^ | 21 | 1.04 | 1.13 | bimodal | -0.07 | 0.60 |
|  | HAmCq^G8^ | 21 | 0.86 | 0.99 | bimodal | 0.30 | 1.07 |
| alpha/beta-Hydrolases | HAmCq^G0^ | 109 | 1.17 | 1.26 | unimodal | -0.07 | 0.54 |
|  | HAmCq^G8^ | 98 | 1.17 | 1.20 | unimodal^*^ | 0.41 | 0.70 |
| Ankyrin repeat | HAmCq^G0^ | 85 | 0.79 | 0.82 | unimodal | -0.53 | 0.29 |
|  | HAmCq^G8^ | 78 | 0.70 | 0.73 | unimodal | -0.38 | 0.44 |
| Arginase/deacetylase | HAmCq^G0^ | 9 | 0.69 | 0.88 | unimodal^*^ | -0.78 | 0.25 |
|  | HAmCq^G8^ | 7 | 1.02 | 0.85 | bimodal | -0.60 | -0.90 |
| ARID-like | HAmCq^G0^ | 6 | 1.12 | 1.09 | unimodal | 1.77 | -1.05 |
|  | HAmCq^G8^ | 6 | 0.82 | 0.74 | unimodal | 0.04 | -1.04 |
| ARM repeat | HAmCq^G0^ | 136 | 1.22 | 1.25 | unimodal | 0.07 | 0.14 |
|  | HAmCq^G8^ | 133 | 1.03 | 1.10 | unimodal | 0.57 | 0.71 |
| C-type lectin-like | HAmCq^G0^ | 33 | 1.17 | 1.39 | multimodal | -1.06 | 0.40 |
|  | HAmCq^G8^ | 28 | 1.50 | 1.41 | multimodal | -0.72 | 0.20 |
| C2H2 and C2HC zinc fingers | HAmCq^G0^ | 515 | 0.71 | 0.73 | unimodal | 0.06 | 0.46 |
|  | HAmCq^G8^ | 495 | 0.50 | 0.56 | unimodal | 0.55 | 0.71 |
| Cadherin-like | HAmCq^G0^ | 14 | 1.07 | 1.00 | unimodal^*^ | -1.05 | -0.10 |
|  | HAmCq^G8^ | 14 | 0.80 | 0.86 | bimodal | -0.91 | 0.49 |
| Calcium ATPase, | HAmCq^G0^ | 14 | 1.36 | 1.48 | bimodal | -0.81 | 0.33 |
| transmembrane domain M | HAmCq^G8^ | 12 | 1.20 | 1.47 | bimodal | 0.75 | 1.18 |
| cAMP-binding domain-like | HAmCq^G0^ | 15 | 0.69 | 0.81 | bimodal | -1.49 | 0.28 |
|  | HAmCq^G8^ | 14 | 0.49 | 0.48 | unimodal^*^ | -1.30 | 0.35 |
| Chaperone J-domain | HAmCq^G0^ | 20 | 1.20 | 1.29 | unimodal^*^ | -0.97 | 0.12 |
|  | HAmCq^G8^ | 21 | 1.07 | 1.02 | unimodal^*^ | 0.10 | 0.54 |
| Chemosensory protein Csp2 | HAmCq^G0^ | 28 | 1.81 | 1.71 | bimodal | -0.94 | -0.20 |
|  | HAmCq^G8^ | 23 | 1.66 | 1.64 | bimodal | -1.52 | -0.04 |
| Concanavalin A-like | HAmCq^G0^ | 45 | 0.85 | 0.95 | unimodal^*^ | 0.58 | 0.77 |
| lectins/glucanases | HAmCq^G8^ | 39 | 0.73 | 0.92 | unimodal | 0.04 | 0.89 |
| CRAL/TRIO domain | HAmCq^G0^ | 48 | 1.17 | 1.18 | unimodal | 0.56 | 0.54 |
|  | HAmCq^G8^ | 43 | 1.08 | 1.04 | unimodal^*^ | 1.11 | 0.73 |
| Cysteine proteinases | HAmCq^G0^ | 62 | 1.27 | 1.31 | unimodal | 0.37 | 0.65 |
|  | HAmCq^G8^ | 60 | 1.08 | 1.17 | unimodal | 0.57 | 0.76 |
| Cytochrome b5-like | HAmCq^G0^ | 11 | 1.50 | 1.42 | unimodal | -0.60 | -0.35 |
| heme/steroid binding domain | HAmCq^G8^ | 12 | 1.17 | 1.15 | bimodal | -0.18 | -0.51 |
| Cytochrome P450 | HAmCq^G0^ | 143 | 1.10 | 1.11 | unimodal | -0.68 | 0.08 |
|  | HAmCq^G8^ | 136 | 1.21 | 1.22 | unimodal | -0.70 | 0.20 |
| Di-copper center-containing | HAmCq^G0^ | 14 | 2.13 | 2.41 | multimodal | 1.25 | -0.22 |
| domain | HAmCq^G8^ | 13 | 3.01 | 3.06 | multimodal | -0.91 | 0.19 |
| DNA/RNA polymerases | HAmCq^G0^ | 11 | 0.81 | 0.89 | unimodal | 1.07 | -0.10 |
|  | HAmCq^G8^ | 10 | 0.69 | 0.80 | unimodal | -0.25 | 0.84 |
| E set domains | HAmCq^G0^ | 51 | 1.56 | 1.74 | unimodal | -0.26 | 0.62 |
|  | HAmCq^G8^ | 49 | 1.39 | 1.69 | unimodal | -0.40 | 0.65 |
| EF-hand | HAmCq^G0^ | 51 | 1.19 | 1.30 | unimodal | -0.40 | 0.60 |
|  | HAmCq^G8^ | 48 | 0.88 | 1.24 | unimodal | -0.14 | 0.94 |
| EGF/Laminin | HAmCq^G0^ | 15 | 0.96 | 1.00 | unimodal | 1.73 | 0.97 |
|  | HAmCq^G8^ | 14 | 0.49 | 0.62 | unimodal^*^ | -1.10 | 0.58 |
| F-box domain | HAmCq^G0^ | 19 | 0.72 | 0.76 | unimodal^*^ | -0.78 | 0.45 |
|  | HAmCq^G8^ | 18 | 0.57 | 0.66 | unimodal | 1.10 | 1.00 |
| FAD-binding domain | HAmCq^G0^ | 7 | 0.36 | 0.65 | bimodal | 0.93 | 1.32 |
|  | HAmCq^G8^ | 7 | 0.59 | 0.90 | bimodal | 0.63 | 1.24 |
| FAD/NAD(P)-binding domain | HAmCq^G0^ | 51 | 1.28 | 1.27 | unimodal | -0.20 | -0.03 |
|  | HAmCq^G8^ | 48 | 1.15 | 1.18 | unimodal^*^ | -0.13 | 0.53 |
| Family A G protein-coupled | HAmCq^G0^ | 31 | 0.59 | 0.56 | unimodal^*^ | 0.96 | 0.86 |
| receptor-like | HAmCq^G8^ | 21 | 0.27 | 0.47 | unimodal | 2.06 | 1.50 |
| Ferritin-like | HAmCq^G0^ | 14 | 1.69 | 1.67 | bimodal | -0.02 | 0.54 |
|  | HAmCq^G8^ | 14 | 1.69 | 1.79 | unimodal^*^ | -1.02 | 0.45 |
| Fibrinogen C-terminal domain- | HAmCq^G0^ | 42 | 0.99 | 1.03 | unimodal^*^ | -0.66 | 0.41 |
| like | HAmCq^G8^ | 38 | 0.94 | 0.94 | unimodal^*^ | -0.65 | 0.27 |
| Fibronectin type III | HAmCq^G0^ | 32 | 0.55 | 0.64 | unimodal^*^ | -0.30 | 0.54 |
|  | HAmCq^G8^ | 26 | 0.53 | 0.51 | unimodal^*^ | -0.51 | 0.46 |
| FnI-like domain | HAmCq^G0^ | 9 | 0.95 | 0.93 | unimodal | 0.70 | 0.41 |
|  | HAmCq^G8^ | 7 | 0.57 | 0.80 | unimodal^*^ | 1.47 | 1.46 |
| FYVE/PHD zinc finger | HAmCq^G0^ | 32 | 1.09 | 0.95 | unimodal^*^ | -0.55 | -0.64 |
|  | HAmCq^G8^ | 32 | 0.76 | 0.74 | unimodal | 1.24 | 0.44 |
| Galactose mutarotase-like | HAmCq^G0^ | 7 | 1.68 | 1.36 | multimodal | -0.57 | -0.71 |
|  | HAmCq^G8^ | 7 | 1.38 | 1.31 | multimodal | -0.04 | 0.72 |
| Glucocorticoid receptor-like | HAmCq^G0^ | 154 | 0.85 | 0.87 | unimodal | 0.99 | 0.63 |
| (DNA-binding domain) | HAmCq^G8^ | 147 | 0.66 | 0.72 | unimodal | 1.54 | 1.02 |
| Glutamine synthetase/guanido | HAmCq^G0^ | 5 | 1.75 | 1.92 | bimodal | 1.20 | 1.04 |
| kinase | HAmCq^G8^ | 5 | 1.56 | 1.99 | bimodal | 2.73 | 1.69 |
| Glutathione S-transferase | HAmCq^G0^ | 29 | 1.66 | 1.57 | unimodal | -0.20 | -0.59 |
| (GST), C-terminal domain | HAmCq^G8^ | 27 | 1.54 | 1.52 | unimodal^*^ | -0.96 | -0.07 |
| Growth factor receptor domain | HAmCq^G0^ | 11 | 1.18 | 1.09 | unimodal | 0.14 | 0.30 |
|  | HAmCq^G8^ | 11 | 0.77 | 0.92 | unimodal^*^ | -0.35 | 0.88 |
| HAD-like | HAmCq^G0^ | 21 | 1.23 | 1.26 | unimodal | 1.37 | 0.59 |
|  | HAmCq^G8^ | 22 | 0.97 | 1.01 | unimodal | 1.76 | 0.90 |
| Histone-fold | HAmCq^G0^ | 43 | 0.74 | 0.80 | unimodal^*^ | 1.28 | 1.01 |
|  | HAmCq^G8^ | 32 | 0.64 | 0.70 | unimodal | 1.86 | 1.16 |
| HLH, helix-loop-helix DNA- | HAmCq^G0^ | 32 | 1.19 | 1.08 | unimodal^*^ | -0.64 | 0.06 |
| binding domain | HAmCq^G8^ | 28 | 0.81 | 0.90 | unimodal^*^ | -0.88 | 0.33 |
| HMG-box | HAmCq^G0^ | 23 | 0.98 | 1.06 | unimodal | 1.80 | 0.63 |
|  | HAmCq^G8^ | 23 | 0.60 | 0.74 | unimodal^*^ | 1.52 | 0.99 |
| Homeodomain-like | HAmCq^G0^ | 85 | 0.66 | 0.66 | unimodal | -0.52 | 0.35 |
|  | HAmCq^G8^ | 57 | 0.54 | 0.57 | unimodal | 0.12 | 0.69 |
| Immunoglobulin | HAmCq^G0^ | 61 | 0.54 | 0.64 | unimodal^*^ | -0.53 | 0.69 |
|  | HAmCq^G8^ | 30 | 0.65 | 0.68 | unimodal | -0.90 | 0.32 |
| Insect pheromone/odorant- | HAmCq^G0^ | 47 | 0.97 | 1.14 | unimodal | 0.01 | 0.76 |
| binding proteins | HAmCq^G8^ | 39 | 1.00 | 1.14 | unimodal^*^ | 1.31 | 1.06 |
| Invertebrate chitin-binding | HAmCq^G0^ | 103 | 2.19 | 2.03 | unimodal^*^ | -0.44 | -0.40 |
|  | HAmCq^G8^ | 102 | 2.05 | 1.87 | unimodal^*^ | -0.63 | -0.38 |
| L domain-like | HAmCq^G0^ | 102 | 0.97 | 0.99 | unimodal | -0.68 | 0.27 |
|  | HAmCq^G8^ | 87 | 0.99 | 1.01 | unimodal | 0.55 | 0.62 |
| Ligand-binding domain in NO | HAmCq^G0^ | 6 | 1.12 | 0.99 | bimodal | -2.30 | -0.34 |
| signaling and Golgi transport | HAmCq^G8^ | 6 | 0.70 | 0.72 | unimodal^*^ | -1.82 | 0.24 |
| Lipocalins | HAmCq^G0^ | 15 | 1.30 | 1.42 | unimodal^*^ | -0.60 | 0.25 |
|  | HAmCq^G8^ | 15 | 1.36 | 1.36 | multimodal | 0.55 | -0.05 |
| Lysozyme-like | HAmCq^G0^ | 4 | 1.40 | 1.48 | unimodal | 3.22 | 1.70 |
|  | HAmCq^G8^ | 5 | 1.58 | 1.49 | bimodal | 2.67 | -1.43 |
| Metallo-dependent hydrolases | HAmCq^G0^ | 11 | 1.41 | 1.44 | unimodal | -0.59 | -0.09 |
|  | HAmCq^G8^ | 11 | 1.21 | 1.20 | unimodal^*^ | 0.02 | -0.60 |
| Metallo-dependent | HAmCq^G0^ | 23 | 1.28 | 1.34 | unimodal | 0.38 | 0.51 |
| phosphatases | HAmCq^G8^ | 22 | 1.16 | 1.26 | unimodal^*^ | 0.09 | 0.63 |
| Metalloproteases ("zincins") | HAmCq^G0^ | 49 | 1.40 | 1.33 | unimodal^*^ | -0.91 | 0.11 |
|  | HAmCq^G8^ | 47 | 1.35 | 1.38 | unimodal^*^ | -1.01 | 0.25 |
| MFS general transporter | HAmCq^G0^ | 148 | 1.08 | 1.05 | unimodal | 0.41 | 0.42 |
|  | HAmCq^G8^ | 140 | 0.87 | 0.96 | unimodal | 0.94 | 0.66 |
| Myosin rod fragments | HAmCq^G0^ | 5 | 1.31 | 1.26 | multimodal | 0.88 | -0.20 |
|  | HAmCq^G8^ | 5 | 1.12 | 1.16 | bimodal | 1.51 | 0.95 |
| N-acetylmuramoyl-L-alanine | HAmCq^G0^ | 9 | 1.56 | 1.70 | multimodal | -0.05 | 1.18 |
| amidase-like | HAmCq^G8^ | 10 | 0.90 | 1.42 | bimodal | 0.56 | 1.33 |
| NAD(P)-binding Rossmann | HAmCq^G0^ | 119 | 1.45 | 1.39 | unimodal^*^ | -0.16 | -0.11 |
|  | HAmCq^G8^ | 115 | 1.23 | 1.25 | unimodal | 0.51 | 0.22 |
| NAD(P)-linked oxidoreductase | HAmCq^G0^ | 15 | 1.47 | 1.45 | unimodal^*^ | -0.22 | -0.55 |
|  | HAmCq^G8^ | 14 | 1.28 | 1.29 | unimodal | 0.22 | -0.30 |
| NAP-like | HAmCq^G0^ | 8 | 0.73 | 0.95 | multimodal | 0.22 | 0.96 |
|  | HAmCq^G8^ | 8 | 0.85 | 0.94 | bimodal | 2.98 | 1.36 |
| Neurotransmitter-gated ion- | HAmCq^G0^ | 10 | 0.34 | 0.50 | unimodal | 0.17 | 0.99 |
| channel transmembrane pore | HAmCq^G8^ | 3 | 0.32 | 0.53 | bimodal | 0.00 | 1.38 |
| Nicotinic receptor ligand | HAmCq^G0^ | 11 | 0.62 | 0.69 | unimodal^*^ | -0.93 | 0.66 |
| binding domain-like | HAmCq^G8^ | 7 | 0.94 | 0.90 | unimodal^*^ | -0.53 | 0.09 |
| Nuclear receptor ligand-bind | HAmCq^G0^ | 18 | 0.94 | 0.81 | bimodal | -1.57 | -0.21 |
|  | HAmCq^G8^ | 12 | 0.83 | 0.74 | unimodal^*^ | -0.56 | -0.36 |
| Nucleotide-diPO4-sugar transf | HAmCq^G0^ | 38 | 0.96 | 1.03 | unimodal | -0.38 | 0.50 |
|  | HAmCq^G8^ | 39 | 0.81 | 0.84 | unimodal | -0.01 | 0.61 |
| Outer arm dynein light chain 1 | HAmCq^G0^ | 7 | 0.96 | 0.86 | unimodal^*^ | -0.83 | -0.18 |
|  | HAmCq^G8^ | 8 | 0.62 | 0.61 | unimodal | -1.21 | 0.20 |
| P-loop nucleotide hydrolases | HAmCq^G0^ | 427 | 1.09 | 1.14 | unimodal | 1.89 | 0.89 |
|  | HAmCq^G8^ | 407 | 0.90 | 0.99 | unimodal | 3.57 | 1.41 |
| PDZ domain-like | HAmCq^G0^ | 44 | 0.92 | 0.97 | unimodal | -0.50 | 0.07 |
|  | HAmCq^G8^ | 36 | 0.74 | 0.79 | unimodal | -0.41 | 0.48 |
| Phosphoglycerate mutase-like | HAmCq^G0^ | 15 | 1.32 | 1.29 | multimodal | 0.08 | -0.35 |
|  | HAmCq^G8^ | 14 | 1.14 | 1.24 | unimodal | 2.17 | 1.23 |
| Phospholipase A2, PLA2 | HAmCq^G0^ | 6 | 0.85 | 0.87 | unimodal | 1.33 | 0.29 |
|  | HAmCq^G8^ | 7 | 0.34 | 0.31 | unimodal | 4.53 | -1.96 |
| PLC-like phosphodiesterases | HAmCq^G0^ | 9 | 0.79 | 0.87 | unimodal^*^ | -0.94 | 0.10 |
|  | HAmCq^G8^ | 8 | 0.67 | 0.86 | bimodal | 1.14 | 1.18 |
| PLP-binding barrel | HAmCq^G0^ | 7 | 0.92 | 1.20 | bimodal | 4.16 | 2.04 |
|  | HAmCq^G8^ | 6 | 1.14 | 1.33 | bimodal | 3.56 | 1.86 |
| PLP-dependent transferases | HAmCq^G0^ | 36 | 1.74 | 1.65 | bimodal | -0.52 | -0.40 |
|  | HAmCq^G8^ | 38 | 1.61 | 1.49 | bimodal | -0.76 | -0.39 |
| PR-1-like | HAmCq^G0^ | 7 | 0.80 | 0.86 | multimodal | -1.39 | 0.29 |
|  | HAmCq^G8^ | 8 | 0.70 | 0.95 | bimodal | -1.91 | 0.32 |
| Protein kinase-like (PK-like) | HAmCq^G0^ | 254 | 0.97 | 0.98 | bimodal | 0.52 | 0.53 |
|  | HAmCq^G8^ | 247 | 0.79 | 0.84 | bimodal | 0.27 | 0.58 |
| Proton glutamate symporter | HAmCq^G0^ | 5 | 0.95 | 0.84 | unimodal | 0.51 | -0.85 |
|  | HAmCq^G8^ | 4 | 0.92 | 1.00 | unimodal | 2.51 | 1.59 |
| Quinoprotein ADH-like | HAmCq^G0^ | 14 | 1.10 | 1.02 | bimodal | 0.20 | -0.93 |
|  | HAmCq^G8^ | 13 | 0.82 | 0.91 | unimodal^*^ | 0.12 | -0.53 |
| Retrovirus zinc finger-like | HAmCq^G0^ | 10 | 1.06 | 1.08 | bimodal | 4.49 | 1.83 |
|  | HAmCq^G8^ | 10 | 0.99 | 1.00 | bimodal | 3.18 | 0.86 |
| Ribonuclease H-like | HAmCq^G0^ | 27 | 1.12 | 1.13 | unimodal | -0.71 | -0.26 |
|  | HAmCq^G8^ | 27 | 0.89 | 0.98 | unimodal | -0.39 | -0.37 |
| RING/U-box | HAmCq^G0^ | 90 | 1.12 | 1.08 | unimodal | 1.17 | 0.41 |
|  | HAmCq^G8^ | 92 | 0.82 | 0.83 | unimodal | 0.97 | 0.52 |
| RNA-binding domain | HAmCq^G0^ | 131 | 1.25 | 1.23 | unimodal | -0.02 | -0.06 |
|  | HAmCq^G8^ | 120 | 1.01 | 1.04 | unimodal | 0.46 | 0.30 |
| RNI-like | HAmCq^G0^ | 83 | 0.71 | 0.73 | unimodal | -0.80 | 0.18 |
|  | HAmCq^G8^ | 80 | 0.63 | 0.68 | unimodal | -0.78 | 0.21 |
| SAM-methyltransferases | HAmCq^G0^ | 68 | 1.14 | 1.13 | unimodal | -0.16 | -0.18 |
|  | HAmCq^G8^ | 67 | 0.93 | 0.93 | unimodal | 0.26 | 0.26 |
| Serine protease inhibitors | HAmCq^G0^ | 5 | 2.39 | 1.97 | bimodal | -2.70 | -0.59 |
|  | HAmCq^G8^ | 5 | 2.55 | 2.03 | bimodal | 3.08 | -1.77 |
| SET domain | HAmCq^G0^ | 40 | 0.91 | 0.82 | unimodal^*^ | -1.05 | -0.13 |
|  | HAmCq^G8^ | 43 | 0.71 | 0.63 | unimodal | -0.92 | 0.16 |
| Six-hairpin glycosidases | HAmCq^G0^ | 11 | 1.46 | 1.48 | unimodal^*^ | -0.06 | -0.56 |
|  | HAmCq^G8^ | 10 | 1.71 | 1.64 | unimodal^*^ | -0.99 | -0.01 |
| Sterol carrier protein | HAmCq^G0^ | 7 | 3.32 | 2.65 | bimodal | -1.04 | -0.97 |
|  | HAmCq^G8^ | 7 | 3.40 | 2.70 | multimodal | -1.12 | -0.82 |
| Terpenoid cyclases | HAmCq^G0^ | 6 | 1.30 | 1.13 | unimodal^*^ | -1.69 | -0.86 |
|  | HAmCq^G8^ | 6 | 1.10 | 1.07 | unimodal^*^ | -0.53 | 0.27 |
| Thiolase-like | HAmCq^G0^ | 12 | 1.73 | 1.70 | unimodal | -1.03 | -0.14 |
|  | HAmCq^G8^ | 12 | 1.58 | 1.58 | unimodal | -1.11 | 0.22 |
| Thioredoxin-like | HAmCq^G0^ | 58 | 1.64 | 1.69 | unimodal | 0.62 | -0.40 |
|  | HAmCq^G8^ | 58 | 1.52 | 1.50 | unimodal | -0.50 | -0.20 |
| TPR-like | HAmCq^G0^ | 74 | 1.17 | 1.17 | unimodal | 0.17 | -0.14 |
|  | HAmCq^G8^ | 67 | 1.07 | 1.10 | unimodal | 0.75 | 0.37 |
| TRAF domain-like | HAmCq^G0^ | 16 | 1.00 | 0.88 | unimodal | -0.17 | -0.73 |
|  | HAmCq^G8^ | 15 | 0.87 | 0.86 | unimodal | -1.04 | 0.20 |
| Translation proteins | HAmCq^G0^ | 13 | 0.51 | 1.19 | bimodal | 0.03 | 1.15 |
|  | HAmCq^G8^ | 9 | 0.92 | 1.54 | bimodal | -1.02 | 0.87 |
| Tropomyosin | HAmCq^G0^ | 9 | 0.93 | 1.12 | bimodal | 2.29 | 1.40 |
|  | HAmCq^G8^ | 11 | 0.68 | 0.92 | bimodal | 5.73 | 2.21 |
| Trypsin-like serine proteases | HAmCq^G0^ | 217 | 1.22 | 1.27 | unimodal | 0.06 | 0.66 |
|  | HAmCq^G8^ | 205 | 1.07 | 1.20 | unimodal | 0.18 | 0.85 |
| Tubulin nucleotide-binding | HAmCq^G0^ | 10 | 1.97 | 1.90 | multimodal | -1.00 | -0.51 |
|  | HAmCq^G8^ | 11 | 1.80 | 1.63 | unimodal^*^ | -1.07 | -0.12 |
| UBA-like | HAmCq^G0^ | 11 | 1.03 | 1.09 | unimodal | 2.27 | 0.17 |
|  | HAmCq^G8^ | 10 | 1.05 | 1.01 | unimodal | -0.08 | 0.42 |
| Ubiquitin-like | HAmCq^G0^ | 33 | 1.42 | 1.60 | unimodal | -0.22 | 0.47 |
|  | HAmCq^G8^ | 32 | 1.36 | 1.48 | unimodal | 0.11 | 0.66 |
| UDP-Glycosyltransferase | HAmCq^G0^ | 29 | 1.38 | 1.29 | unimodal | 0.32 | -0.52 |
|  | HAmCq^G8^ | 33 | 1.08 | 1.08 | unimodal^*^ | -1.08 | -0.09 |
| vWA-like | HAmCq^G0^ | 13 | 1.02 | 1.11 | unimodal^*^ | -0.86 | 0.10 |
|  | HAmCq^G8^ | 14 | 0.92 | 0.96 | unimodal^*^ | -0.94 | -0.15 |
| WD40 repeat-like | HAmCq^G0^ | 183 | 1.06 | 1.06 | unimodal | 1.91 | 0.65 |
|  | HAmCq^G8^ | 173 | 0.89 | 0.91 | unimodal | 3.28 | 1.06 |
| Winged helix DNA-binding | HAmCq^G0^ | 57 | 1.20 | 1.26 | unimodal^*^ | 0.89 | 0.55 |
|  | HAmCq^G8^ | 56 | 0.92 | 1.07 | unimodal^*^ | 1.41 | 0.96 |
| WW domain | HAmCq^G0^ | 10 | 0.86 | 0.99 | unimodal^*^ | -0.28 | 0.62 |
|  | HAmCq^G8^ | 11 | 0.67 | 0.64 | unimodal | 1.01 | 1.11 |
| Zn-dependent exopeptidases | HAmCq^G0^ | 40 | 1.54 | 1.58 | unimodal | 0.86 | -0.01 |
|  | HAmCq^G8^ | 41 | 1.54 | 1.54 | unimodal | 0.15 | -0.38 |
| ^**^Carboxylesterases | HAmCq^G0^ | 17 | 1 | 1.12 | unimodal^*^ | 1.19 | 0.94 |
|  | HAmCq^G8^ | 16 | 0.71 | 1.03 | bimodal | 3.22 | 1.51 |

^†^Superfamilies from Structural Classification of Proteins (v1.73, <http://supfam.cs.bris.ac.uk/SUPERFAMILY/function.html>)

^‡^Total number of genes detected within the superfamily

^*^unimodal, but shouldered distribution

^**^Not a SCOP superfamily classification. Genes were grouped based on Vectorbase annotation as carboxylesterases.
