# Supplementary material for: The Transcriptome Profile of the Mosquito Culex quinquefasciatus following Permethrin Selection
Source: PLoS One. 2012 Oct 5;7(10):e47163. doi: 10.1371/journal.pone.0047163 (PMC3465273; doi:10.1371/journal.pone.0047163)
Supplement: Table S3 — Complete list of all differentially upregulated genes† in HAmCqG8. (DOC) [file pone.0047163.s003.doc]

Table S3: Complete list of all differentially upregulated genes^†^ in HAmCq^G8^.

| General function^*^ | Detailed function | Superfamily | Gene accession^**^ | CpipJ_1.2 annotation | HAmCq^G8^ FPKM | Relative FPKM^#^ |
| --- | --- | --- | --- | --- | --- | --- |
| Extra-cellular processes | Cell adhesion | C-type lectin-like | CPIJ000449 | galactose-specific C-type lectin | 52.0 | 38.6 |
|  |  |  | CPIJ015401 | galactose-specific C-type lectin | 54.1 | 42.6 |
|  |  |  | CPIJ017075 | galactose-specific C-type lectin | 2.2 | - |
|  |  |  | CPIJ019507 | salivary C-type lectin | 23.8 | 5.2 |
|  |  | Cadherin-like | CPIJ014101 | conserved hypothetical protein | 56.6 | 2.7 |
|  |  | EGF/Laminin | CPIJ014886 | conserved hypothetical protein | 5.2 | 4.4 |
|  | Blood clotting | Fibrinogen C-terminal domain-like | CPIJ010089 | microfibril-associated glycoprotein 4 | 28.8 | 2.9 |
|  |  |  | CPIJ012830 | fibrinogen and fibronectin | 121.8 | 6.7 |
|  |  |  | CPIJ013294 | fibrinogen and fibronectin | 11.1 | - |
|  |  |  | CPIJ015014 | conserved hypothetical protein | 9.4 | 8.3 |
|  |  |  | CPIJ018159 | fibrinogen and fibronectin | 15.1 | 3.8 |
|  | Cell adhesion | Fibronectin type III | CPIJ000838 | conserved hypothetical protein | 2.7 | 7.6 |
|  |  | FnI-like domain | CPIJ013195 | conserved hypothetical protein | 53.0 | 2.4 |
|  |  | RNI-like | CPIJ002173 | conserved hypothetical protein | 16.0 | 3.2 |
|  |  |  | CPIJ004947 | leucine-rich repeat-containing protein 1 | 58.7 | 3.5 |
|  |  |  | CPIJ011874 | predicted protein | 1.3 | - |
|  |  |  | CPIJ014115 | conserved hypothetical protein | 10.7 | 4.0 |
|  |  |  | CPIJ014953 | membrane glycoprotein LIG-1 | 10.5 | 4.0 |
|  |  |  | CPIJ016528 | conserved hypothetical protein | 1.0 | - |
|  |  |  | CPIJ019556 | predicted protein | 8.9 | - |
| General | Protein interaction | Ankyrin repeat | CPIJ003373 | predicted protein | 15.5 | 12.0 |
|  |  |  | CPIJ008490 | ankyrin repeat domain-containing protein 44 | 1.3 | - |
|  |  |  | CPIJ009398 | conserved hypothetical protein | 4.6 | 3.4 |
|  | General | ARM repeat | CPIJ005388 | conserved hypothetical protein | 4.0 | 2.8 |
|  |  | EF-hand | CPIJ001560 | calcium-binding protein | 1097.9 | 4.4 |
|  |  |  | CPIJ012250 | troponin C | 567.7 | 6.3 |
|  |  |  | CPIJ016821 | troponin C | 497.5 | 4.5 |
|  |  |  | CPIJ019636 | EF-hand calcium-binding domain-containing protein 1 | 1.5 | - |
|  | Protein interaction | F-box domain | CPIJ019555 | predicted protein | 11.4 | 34.5 |
|  | Small molecule binding | FAD-binding domain | CPIJ001318 | d-lactate dehydrognease 2 | 40.5 | 2.2 |
|  |  |  | CPIJ013647 | alkyldihydroxyacetonephosphate synthase | 4.3 | 3.3 |
|  |  |  | CPIJ016321 | alkyldihydroxyacetonephosphate synthase | 3.7 | 3.1 |
|  |  |  | CPIJ016322 | alkyldihydroxyacetonephosphate synthase | 3.9 | 3.3 |
|  |  | FAD/NAD(P)-binding domain | CPIJ007620 | choline dehydrogenase | 132.3 | 2.9 |
|  |  |  | CPIJ008048 | peroxisomal N1-acetyl-spermine/spermidine oxidase | 14.5 | 2.6 |
|  |  |  | CPIJ008445 | amine oxidase | 21.4 | 2.8 |
|  |  |  | CPIJ017813 | spermine oxidase | 8.8 | 2.7 |
|  |  | Glutathione S-transferase (GST), C-terminal domain | CPIJ002663 | glutathione S-transferase 1-1 | 354.4 | 2.2 |
|  |  |  | CPIJ006160 | glutathione s-transferase | 173.7 | 2.4 |
|  |  |  | CPIJ018631 | glutathione-s-transferase theta, gst | 8.3 | 3.7 |
|  | General | L domain-like | CPIJ000315 | conserved hypothetical protein | 1.0 | - |
|  |  |  | CPIJ003143 | conserved hypothetical protein | 14.0 | 2.9 |
|  |  |  | CPIJ004946 | leucine-rich repeat-containing protein 15 | 11.0 | 2.5 |
|  |  |  | CPIJ017510 | conserved hypothetical protein | 914.6 | 7.3 |
|  | Small molecule binding | NAD(P)-binding Rossmann-fold domains | CPIJ003802 | NADP-dependent leukotriene B4 12-hydroxydehydrogenase | 169.2 | 4.0 |
|  |  |  | CPIJ004379 | steroid dehydrogenase | 10.5 | 12.7 |
|  |  | P-loop containing nucleoside triphosphate hydrolases | CPIJ000853 | myosin heavy chain | 1750.5 | 3.8 |
|  |  |  | CPIJ001520 | multidrug resistance-associated protein 1 | 34.6 | 2.7 |
|  |  |  | CPIJ003262 | zinc finger protein | 1.2 | - |
|  |  |  | CPIJ004695 | dynein-1-beta heavy chain | 1.9 | 2.3 |
|  |  |  | CPIJ009034 | conserved hypothetical protein | 27.0 | 2.3 |
|  |  |  | CPIJ009593 | conserved hypothetical protein | 8.0 | 2.9 |
|  |  |  | CPIJ015649 | DNA-binding protein smubp-2 | 1.8 | 4.0 |
|  |  |  | CPIJ019948 | myosin vii | 5.9 | 2.5 |
|  | Protein interaction | TPR-like | CPIJ007346 | TTC27 protein | 37.5 | 2.7 |
|  |  | UBA-like | CPIJ011358 | conserved hypothetical protein | 27.9 | 2.8 |
|  | General | Ubiquitin-like | CPIJ014273 | conserved hypothetical protein | 1.1 | - |
|  |  | WD40 repeat-like | CPIJ006339 | receptor of activated protein kinase C 1 | 6.9 | 5.3 |
|  |  |  | CPIJ012294 | conserved hypothetical protein | 2.0 | 3.5 |
| Information | DNA replication/repair | FYVE/PHD zinc finger | CPIJ002070 | conserved hypothetical protein | 66.0 | 4.0 |
|  |  |  | CPIJ011117 | conserved hypothetical protein | 4.3 | 19.5 |
|  | Chromatin structure | NAP-like | CPIJ007782 | nucleosome assembly protein | 7.5 | 3.2 |
|  | DNA replication/repair | RING/U-box | CPIJ000388 | ubiquitin conjugating enzyme 7 interacting protein | 17.4 | 2.9 |
|  | Chromatin structure | Smc hinge domain | CPIJ018617 | structural maintenance of chromosomes protein 3 | 10.4 | 2.8 |
| Intra-cellular processes | Transport | Ammonium transporter | CPIJ013531 | ammonium transporter | 31.9 | 3.0 |
|  | Phospholipid m/tr | CRAL/TRIO domain | CPIJ001321 | conserved hypothetical protein | 1.7 | - |
|  |  |  | CPIJ003223 | conserved hypothetical protein | 43.4 | 2.2 |
|  |  |  | CPIJ009746 | conserved hypothetical protein | 54.3 | 2.6 |
|  |  |  | CPIJ014226 | cellular retinaldehyde-binding protein | 838.3 | 2.9 |
|  | Proteases | Cysteine proteinases | CPIJ001239 | cathepsin B | 190.2 | 9.7 |
|  |  |  | CPIJ001240 | cathepsin B-like thiol protease | 99.7 | 5.3 |
|  | Ion m/tr | Ferritin-like | CPIJ014287 | ferritin heavy chain | 202.2 | 4.0 |
|  | Transport | Glycolipid transfer protein, GLTP | CPIJ003328 | conserved hypothetical protein | 29.9 | 2.5 |
|  |  | Lipocalins | CPIJ013296 | conserved hypothetical protein | 29.2 | 2.7 |
|  |  |  | CPIJ015725 | apolipoprotein D | 457.6 | 4.6 |
|  | Proteases | Metallo-dependent phosphatases | CPIJ018314 | 5' nucleotidase | 43.6 | 2.8 |
|  |  | Metalloproteases ("zincins"), catalytic domain | CPIJ001050 | protease m1 zinc metalloprotease | 266.9 | 3.6 |
|  |  |  | CPIJ002941 | high choriolytic enzyme 1 | 273.8 | 3.5 |
|  |  |  | CPIJ002942 | zinc metalloproteinase nas-12 | 1025.0 | 2.9 |
|  |  |  | CPIJ002943 | conserved hypothetical protein | 202.6 | 4.3 |
|  |  |  | CPIJ002945 | zinc metalloproteinase dpy-31 | 101.1 | 3.8 |
|  |  |  | CPIJ004086 | angiotensin-converting enzyme | 292.0 | 5.7 |
|  |  |  | CPIJ006803 | zinc metalloproteinase nas-7 | 22.5 | 4.5 |
|  |  |  | CPIJ007383 | endothelin-converting enzyme 1 | 20.1 | 2.5 |
|  |  |  | CPIJ009106 | angiotensin-converting enzyme | 209.4 | 2.7 |
|  |  |  | CPIJ012036 | aminopeptidase N | 30.6 | 3.1 |
|  | Ion m/tr | MFS general substrate transporter | CPIJ001774 | synaptic vesicle protein | 4.9 | 3.4 |
|  |  |  | CPIJ001812 | sugar transporter | 3.3 | 4.8 |
|  |  |  | CPIJ005300 | sugar transporter | 2.9 | 9.3 |
|  |  |  | CPIJ005372 | endogenous retrovirus A receptor | 3.1 | 4.4 |
|  |  |  | CPIJ008813 | sodium-dependent phosphate transporter | 4.6 | 2.8 |
|  |  |  | CPIJ014925 | solute carrier family 2 | 20.7 | 2.3 |
|  | Phospholipid m/tr | PLC-like phosphodiesterases | CPIJ002103 | conserved hypothetical protein | 78.7 | 2.4 |
|  | Transport | Proton glutamate symport protein | CPIJ000673 | glutamate transporter | 22.5 | 2.4 |
|  | Proteases | Serine protease inhibitors | CPIJ012287 | hypothetical protein | 89.0 | 4.8 |
|  | Cell motility | Tropomyosin | CPIJ008188 | conserved hypothetical protein | 3.3 | 5.4 |
|  | Proteases | Trypsin-like serine proteases | CPIJ000616 | clip-domain serine protease | 23.8 | 3.4 |
|  |  |  | CPIJ000617 | clip-domain serine protease | 18.1 | 3.6 |
|  |  |  | CPIJ001979 | conserved hypothetical protein | 22.0 | 3.0 |
|  |  |  | CPIJ002128 | mast cell protease 2 | 94.1 | 16.1 |
|  |  |  | CPIJ002133 | trypsin epsilon | 47.8 | 7.9 |
|  |  |  | CPIJ002135 | trypsin alpha-4 | 61.8 | 5.9 |
|  |  |  | CPIJ002137 | serine protease1/2 | 122.4 | 3.4 |
|  |  |  | CPIJ002139 | HzC4 chymotrypsinogen | 559.8 | 4.3 |
|  |  |  | CPIJ002140 | chymotrypsin BI | 205.9 | 3.5 |
|  |  |  | CPIJ002142 | chymotrypsin BI | 5763.6 | 2.8 |
|  |  |  | CPIJ002156 | chymotrypsin BI | 194.8 | 2.8 |
|  |  |  | CPIJ003623 | coagulation factor XII | 15.0 | 7.1 |
|  |  |  | CPIJ004594 | conserved hypothetical protein | 11.0 | 5.9 |
|  |  |  | CPIJ005272 | trypsin 3A1 | 2.4 | - |
|  |  |  | CPIJ006543 | urokinase-type plasminogen activator | 84.5 | 8.2 |
|  |  |  | CPIJ014656 | coagulation factor XII | 5.8 | 3.3 |
|  |  |  | CPIJ016102 | transmembrane protease | 29.2 | 2.7 |
|  |  |  | CPIJ018037 | serine protease | 15.7 | 3.9 |
|  |  |  | CPIJ019428 | trypsin 2 | 36.9 | 3.4 |
|  |  | Zn-dependent exopeptidases | CPIJ001742 | zinc carboxypeptidase | 175.1 | 3.0 |
|  |  |  | CPIJ001743 | carboxypeptidase A2 | 91.2 | 5.4 |
|  |  |  | CPIJ001744 | zinc carboxypeptidase | 195.5 | 3.5 |
|  |  |  | CPIJ001745 | zinc carboxypeptidase | 80.8 | 7.4 |
|  |  |  | CPIJ009738 | conserved hypothetical protein | 73.2 | 2.3 |
|  |  |  | CPIJ010805 | carboxypeptidase A1 | 180.8 | 4.4 |
|  |  |  | CPIJ014110 | conserved hypothetical protein | 35.1 | 2.4 |
| Metabolism | Carbohydrate m/tr | (Trans)glycosidases | CPIJ002104 | plasma alpha-L-fucosidase | 25.6 | 3.4 |
|  |  |  | CPIJ005060 | alpha-amylase B | 3837.1 | 2.3 |
|  |  |  | CPIJ005725 | alpha-amylase A | 171.1 | 3.1 |
|  |  |  | CPIJ006166 | deltamethrin resistance-associated NYD-GBE | 98.8 | 2.5 |
|  |  |  | CPIJ008528 | glycoside hydrolase | 7.6 | 3.9 |
|  |  |  | CPIJ009306 | neutral alpha-glucosidase ab | 10.0 | 3.7 |
|  | Other enzymes | 3-carboxy-cis,cis-mucoante lactonizing enzyme | CPIJ013577 | selenium-binding protein 2 | 164.4 | 2.7 |
|  |  | Acetyl-CoA synthetase-like | CPIJ000791 | conserved hypothetical protein | 124.8 | 2.8 |
|  |  |  | CPIJ006459 | long-chain-fatty-acid-CoA ligase | 114.8 | 3.5 |
|  |  |  | CPIJ010716 | luciferin 4-monooxygenase | 31.1 | 2.2 |
|  |  |  | CPIJ015088 | 4-coumarate-CoA ligase 1 | 34.2 | 2.2 |
|  | E- transfer | Acyl-CoA dehydrogenase C-terminal domain-like | CPIJ003059 | acyl-CoA oxidase | 47.2 | 4.0 |
|  | Transferases | Acyl-CoA N-acyltransferases (Nat) | CPIJ011827 | conserved hypothetical protein | 1.8 | - |
|  | Redox | ALDH-like | CPIJ009438 | aldehyde dehydrogenase | 95.0 | 2.6 |
|  | Other enzymes | alpha/beta-Hydrolases | CPIJ002715 | lipase 3 | 10.4 | 3.2 |
|  |  |  | CPIJ004222 | pancreatic triacylglycerol lipase | 1790.9 | 4.7 |
|  |  |  | CPIJ007461 | epoxide hydrolase | 18.3 | 2.5 |
|  |  |  | CPIJ007824 | esterase B1 | 34.4 | 2.2 |
|  |  |  | CPIJ008876 | lysosomal pro-X carboxypeptidase | 901.2 | 3.4 |
|  |  |  | CPIJ016336 | esterase B1 | 32.3 | 2.2 |
|  |  |  | CPIJ019917 | triacylglycerol lipase | 23.0 | 2.8 |
|  | Secondary metabolism | Concanavalin A-like lectins/glucanases | CPIJ004320 | gram-negative bacteria-binding protein 1 | 221.8 | 5.6 |
|  |  |  | CPIJ004323 | gram-negative bacteria binding protein | 293.4 | 4.1 |
|  |  |  | CPIJ006421 | conserved hypothetical protein | 8.8 | 2.5 |
|  |  |  | CPIJ013048 | conserved hypothetical protein | 2.3 | - |
|  | E- transfer | Cytochrome b5-like heme/steroid binding domain | CPIJ004595 | cytochrome b5 | 91.7 | 2.3 |
|  |  |  | CPIJ005308 | conserved hypothetical protein | 1.4 | - |
|  | Redox | Cytochrome P450 | CPIJ002538 | *CYP6AG12 ^£^* | 575.0 | 3.7 |
|  |  |  | CPIJ005952 | *CYP6BB4 ^£^* | 4.0 | 2.8 |
|  |  |  | CPIJ005953 | *CYP6BB3 ^£^* | 74.9 | 2.6 |
|  |  |  | CPIJ005955 | *CYP6P14 ^£^* | 126.6 | 8.2 |
|  |  |  | CPIJ005956 | *CYP6BZ2 ^£^* | 460.1 | 3.3 |
|  |  |  | CPIJ005957 | *CYP6AA9 ^£^* | 84.9 | 6.6 |
|  |  |  | CPIJ005959 | *CYP6AA7 ^£^* | 98.3 | 7.3 |
|  |  |  | CPIJ006721 | *CYP4H37v 1^£^* | 56.7 | 2.3 |
|  |  |  | CPIJ007188 | *CYP4H30 ^£^* | 27.9 | 2.3 |
|  |  |  | CPIJ008566 | *CYP6Z15 ^£^* | 5.4 | 3.3 |
|  |  |  | CPIJ009085 | *CYP6AG13 ^£^* | 5.9 | 3.4 |
|  |  |  | CPIJ009478 | *CYP4D42v1 ^£^* | 59.5 | 2.4 |
|  |  |  | CPIJ010225 | *CYP12F14 ^£^* | 30.8 | 3.9 |
|  |  |  | CPIJ010227 | *CYP12F13 ^£^* | 67.8 | 7.1 |
|  |  |  | CPIJ010537 | *CYP9J45 ^£^* | 109.4 | 4.8 |
|  |  |  | CPIJ010538 | *CYP9J46 ^£^* | 35.3 | 7.5 |
|  |  |  | CPIJ010542 | *CYP9J38 ^£^* | 19.6 | 8.3 |
|  |  |  | CPIJ010543 | *CYP9J40 ^£^* | 297.6 | 7.2 |
|  |  |  | CPIJ010544 | *CYP9J33 ^£^* | 58.9 | 3.1 |
|  |  |  | CPIJ010546 | *CYP9J34 ^£^* | 50.9 | 13.4 |
|  |  |  | CPIJ011127 | *CYP4H34 ^£^* | 9.9 | 2.7 |
|  |  |  | CPIJ012470 | *CYP9AL1 ^£^* | 86.2 | 9.2 |
|  |  |  | CPIJ014218 | *CYP9M10 ^£^* | 771.2 | 3.7 |
|  |  |  | CPIJ015681 | *CYP4H37v2 ^£^* | 42.8 | 2.6 |
|  |  |  | CPIJ015958 | *CYP325BC1 ^£^* | 2.6 | 8.1 |
|  |  |  | CPIJ017243 | *CYP304B4 ^£^* | 73.4 | 3.8 |
|  |  |  | CPIJ017244 | *CYP304B5 ^£^* | 2.7 | 18.9 |
|  |  |  | CPIJ020229 | *CYP4D42v2 ^£^* | 38.1 | 2.4 |
|  |  | Di-copper centre-containing domain | CPIJ000056 | larval serum protein 1 beta chain | 5111.7 | 4.9 |
|  |  |  | CPIJ001820 | larval serum protein 2 | 9964.0 | 4.3 |
|  |  |  | CPIJ005187 | phenoloxidase subunit 1 | 147.6 | 2.6 |
|  |  |  | CPIJ006537 | larval serum protein 1 beta chain | 986.8 | 9.7 |
|  |  |  | CPIJ006538 | larval serum protein 1 beta chain | 1170.3 | 10.3 |
|  |  |  | CPIJ007783 | arylphorin subunit alpha | 3379.4 | 2.8 |
|  |  |  | CPIJ009032 | larval serum protein 2 | 574.1 | 3.3 |
|  |  |  | CPIJ009033 | arylphorin subunit C223 | 47162.3 | 2.4 |
|  |  |  | CPIJ018824 | larval serum protein 1 beta chain | 1024.6 | 7.6 |
|  | Coenzyme m/tr | Dihydropteroate synthetase-like | CPIJ003752 | ficolin-2 | 14.8 | 4.1 |
|  | Other enzymes | FAH | CPIJ017110 | fumarylacetoacetate hydrolase | 46.7 | 2.6 |
|  |  | Fumarate reductase respiratory complex transmembrane subunits | CPIJ004125 | succinate dehydrogenase | 10.6 | 13.5 |
|  |  | Galactose mutarotase-like | CPIJ004867 | conserved hypothetical protein | 4.7 | 3.2 |
|  | Amino acids m/tr | Glutamine synthetase/guanido kinase | CPIJ007538 | arginine kinase | 4762.7 | 3.5 |
|  | Other enzymes | HydA/Nqo6-like | CPIJ018869 | NADH dehydrogenase iron-sulfur protein 7, mitochondrial | 206.2 | 2.7 |
|  | Carbohydrate m/tr | Invertebrate chitin-binding proteins | CPIJ014999 | conserved hypothetical protein | 36.4 | 4.9 |
|  | Lipid m/tr | Lipovitellin-phosvitin complex, superhelical domain | CPIJ001746 | conserved hypothetical protein | 1918.0 | 4.6 |
|  | Other enzymes | Lysozyme-like | CPIJ018802 | endochitinase A | 177.0 | 2.6 |
|  |  |  | CPIJ019598 | basic endochitinase CHB4 | 77.3 | 4.0 |
|  |  | N-acetylmuramoyl-L-alanine amidase-like | CPIJ006560 | peptidoglycan recognition protein-lc | 4.6 | - |
|  | Transferases | Nucleotide-diphospho-sugar transferases | CPIJ001091 | lactosylceramide 4-alpha-galactosyltransferase | 1.1 | - |
|  | Other enzymes | Phosphoglycerate mutase-like | CPIJ014577 | phosphoglycerate mutase 2 | 330.2 | 2.7 |
|  | Amino acids m/tr | PLP-binding barrel | CPIJ009094 | ornithine decarboxylase 1 | 16.0 | 3.0 |
|  | Transferases | PLP-dependent transferases | CPIJ006619 | cystathionine gamma-lyase | 120.6 | 3.4 |
|  | Secondary metabolism | PR-1-like | CPIJ000211 | cysteine-rich secretory protein-2 | 2.5 | - |
|  |  |  | CPIJ004029 | venom allergen 5 | 72.0 | 6.1 |
|  | Other enzymes | Quinoprotein alcohol dehydrogenase-like | CPIJ002052 | WD repeat protein 61 | 28.2 | 2.3 |
|  | Carbohydrate m/tr | Six-hairpin glycosidases | CPIJ008853 | maltose phosphorylase | 53.8 | 2.2 |
|  | Coenzyme m/tr | Sterol carrier protein, SCP | CPIJ012490 | sterol carrier protein 2 | 12774.9 | 3.1 |
|  | Other enzymes | Thiolase-like | CPIJ003495 | fatty acid synthase S-acetyltransferase | 7.3 | 2.7 |
|  | Redox | Thioredoxin-like | CPIJ018667 | NADH dehydrogenase flavoprotein 2, mitochondrial | 116.0 | 2.8 |
|  | Polysaccharide m/tr | UDP-Glycosyltransferase/glycogen phosphorylase | CPIJ000226 | glucosyl/glucuronosyl transferase | 45.1 | 2.3 |
|  |  |  | CPIJ003692 | glucosyl/glucuronosyl transferase | 9.3 | 4.7 |
|  |  |  | CPIJ006508 | UDP-glucuronosyltransferase 2B4 | 58.0 | 3.5 |
|  |  |  | CPIJ015996 | ecdysteroid UDP-glucosyltransferase | 3.2 | 7.5 |
|  | Energy | Vacuolar ATP synthase subunit C | CPIJ002067 | vacuolar ATP synthase subunit C | 147.7 | 2.4 |
| NONA^§^ | not annotated | NONA | CPIJ000008 | chitotriosidase-1 | 64.4 | 3.7 |
|  |  |  | CPIJ000448 | conserved hypothetical protein | 5.4 | 6.9 |
|  |  |  | CPIJ000494 | conserved hypothetical protein | 1406.6 | 3.4 |
|  |  |  | CPIJ000665 | galectin | 223.6 | 3.6 |
|  |  |  | CPIJ000852 | myosin-Id | 810.6 | 3.9 |
|  |  |  | CPIJ000905 | tetraspanin | 58.7 | 2.4 |
|  |  |  | CPIJ001111 | proacrosin | 51.7 | 3.3 |
|  |  |  | CPIJ002056 | adenylate cyclase type 2 | 3.5 | 2.9 |
|  |  |  | CPIJ002117 | conserved hypothetical protein | 71.4 | 2.3 |
|  |  |  | CPIJ002130 | kallikrein-7 | 23.1 | 2.4 |
|  |  |  | CPIJ002138 | chymotrypsinogen | 242.3 | 824.5 |
|  |  |  | CPIJ002168 | conserved hypothetical protein | 43.2 | 2.7 |
|  |  |  | CPIJ002247 | elongation factor-1 alpha | 2.3 | - |
|  |  |  | CPIJ002359 | myomesin | 30.9 | 3.2 |
|  |  |  | CPIJ002361 | sodium/solute symporter | 26.5 | 5.3 |
|  |  |  | CPIJ002406 | conserved hypothetical protein | 2.0 | - |
|  |  |  | CPIJ002882 | conserved hypothetical protein | 7.8 | 2.5 |
|  |  |  | CPIJ003306 | conserved hypothetical protein | 1.8 | 12.6 |
|  |  |  | CPIJ003317 | conserved hypothetical protein | 15.2 | 3.7 |
|  |  |  | CPIJ003338 | beta-galactosidase | 101.7 | 3.8 |
|  |  |  | CPIJ003485 | cuticle protein | 158.6 | 2.6 |
|  |  |  | CPIJ004394 | hypothetical protein | 677.2 | 2.5 |
|  |  |  | CPIJ004558 | conserved hypothetical protein | 6.2 | 22.0 |
|  |  |  | CPIJ004600 | oxidoreductase | 1.5 | - |
|  |  |  | CPIJ004927 | potassium channel kcnq | 2.0 | - |
|  |  |  | CPIJ004976 | conserved hypothetical protein | 5.4 | 5.1 |
|  |  |  | CPIJ005090 | conserved hypothetical protein | 12.7 | 3.5 |
|  |  |  | CPIJ005451 | lysozyme | 141.8 | 2.2 |
|  |  |  | CPIJ005479 | hypothetical protein | 10.1 | 13.7 |
|  |  |  | CPIJ005495 | hypothetical protein | 806.7 | 2.5 |
|  |  |  | CPIJ005656 | oxidoreductase | 58.5 | 2.8 |
|  |  |  | CPIJ005841 | angiopoietin-1 | 118.2 | 8.2 |
|  |  |  | CPIJ006076 | hypodermin-B | 11.5 | 17.0 |
|  |  |  | CPIJ006150 | Toll9 | 30.5 | 3.0 |
|  |  |  | CPIJ006293 | conserved hypothetical protein | 16.5 | 2.3 |
|  |  |  | CPIJ006294 | conserved hypothetical protein | 7.7 | 2.8 |
|  |  |  | CPIJ006393 | conserved hypothetical protein | 6.4 | - |
|  |  |  | CPIJ006515 | Toll9 | 25.6 | 2.5 |
|  |  |  | CPIJ006516 | conserved hypothetical protein | 17.3 | 9.7 |
|  |  |  | CPIJ006542 | chymotrypsin-2 | 64.8 | 19.7 |
|  |  |  | CPIJ006585 | glycoprotein | 27.6 | 3.5 |
|  |  |  | CPIJ006588 | NADH dehydrogenase 1 alpha subcomplex subunit 6 | 6.5 | - |
|  |  |  | CPIJ007033 | lipase | 112.0 | 2.7 |
|  |  |  | CPIJ007035 | lipase | 574.2 | 3.9 |
|  |  |  | CPIJ007382 | hypothetical protein | 7.7 | - |
|  |  |  | CPIJ007432 | sialin | 6.2 | 2.6 |
|  |  |  | CPIJ007683 | adam | 2.5 | - |
|  |  |  | CPIJ007721 | hypothetical protein | 995.1 | 2.9 |
|  |  |  | CPIJ007785 | conserved hypothetical protein | 143.6 | 2.3 |
|  |  |  | CPIJ007966 | conserved hypothetical protein | 19.0 | 2.3 |
|  |  |  | CPIJ008031 | conserved hypothetical protein | 17.7 | 2.5 |
|  |  |  | CPIJ008110 | conserved hypothetical protein | 15.8 | 2.6 |
|  |  |  | CPIJ008379 | conserved hypothetical protein | 583.6 | 2.7 |
|  |  |  | CPIJ008651 | solute carrier family 41 | 23.4 | 2.7 |
|  |  |  | CPIJ008662 | conserved hypothetical protein | 7.0 | 8.6 |
|  |  |  | CPIJ008663 | conserved hypothetical protein | 243.3 | 2.8 |
|  |  |  | CPIJ008807 | ficolin-1 | 3.7 | - |
|  |  |  | CPIJ008858 | conserved hypothetical protein | 38.1 | 3.2 |
|  |  |  | CPIJ008873 | prolylcarboxypeptidase | 83.0 | 3.5 |
|  |  |  | CPIJ008904 | alpha-glucosidase | 24.3 | 2.1 |
|  |  |  | CPIJ009556 | serine threonine-protein kinase | 4.7 | - |
|  |  |  | CPIJ009594 | nephrosin | 26.2 | 21.7 |
|  |  |  | CPIJ009609 | conserved hypothetical protein | 20.7 | 2.6 |
|  |  |  | CPIJ009683 | translocator protein | 106.8 | 2.6 |
|  |  |  | CPIJ009726 | conserved hypothetical protein | 29.9 | 3.1 |
|  |  |  | CPIJ009744 | conserved hypothetical protein | 38.3 | 2.6 |
|  |  |  | CPIJ009902 | predicted protein | 14.3 | 4.8 |
|  |  |  | CPIJ009929 | conserved hypothetical protein | 14.9 | 2.4 |
|  |  |  | CPIJ010224 | metalloproteinase | 29.4 | 2.9 |
|  |  |  | CPIJ010247 | raw | 14.9 | 2.7 |
|  |  |  | CPIJ010305 | CHKov1 | 60.2 | 4.4 |
|  |  |  | CPIJ010426 | nucleoporin | 2.4 | 12.7 |
|  |  |  | CPIJ010563 | conserved hypothetical protein | 2.3 | - |
|  |  |  | CPIJ010641 | prostasin | 104.0 | 24.2 |
|  |  |  | CPIJ010699 | cecropin A | 374.4 | 2.7 |
|  |  |  | CPIJ010757 | conserved hypothetical protein | 325.2 | 3.0 |
|  |  |  | CPIJ010759 | conserved hypothetical protein | 1.4 | - |
|  |  |  | CPIJ010761 | conserved hypothetical protein | 738.5 | 2.8 |
|  |  |  | CPIJ010934 | conserved hypothetical protein | 252.5 | 2.4 |
|  |  |  | CPIJ010987 | conserved hypothetical protein | 2.5 | 3.5 |
|  |  |  | CPIJ011523 | conserved hypothetical protein | 16.0 | 2.8 |
|  |  |  | CPIJ012458 | chromatin assembly factor 1, p180-subunit | 8.8 | 2.6 |
|  |  |  | CPIJ012571 | actin | 1474.9 | 4.4 |
|  |  |  | CPIJ012573 | actin | 4309.3 | 4.8 |
|  |  |  | CPIJ012574 | actin | 97.7 | 2.1 |
|  |  |  | CPIJ012700 | CHKov1 | 5.8 | 5.8 |
|  |  |  | CPIJ012899 | secreted protein | 91.7 | 2.3 |
|  |  |  | CPIJ013085 | sarcalumenin | 167.6 | 2.5 |
|  |  |  | CPIJ013319 | metalloproteinase | 132.2 | 3.5 |
|  |  |  | CPIJ013351 | hypothetical protein | 2.0 | - |
|  |  |  | CPIJ013355 | conserved hypothetical protein | 55.0 | 2.4 |
|  |  |  | CPIJ013736 | hypothetical protein | 1306.7 | 3.1 |
|  |  |  | CPIJ014184 | conserved hypothetical protein | 270.6 | - |
|  |  |  | CPIJ014236 | conserved hypothetical protein | 1.1 | - |
|  |  |  | CPIJ014523 | elastase-3A | 18.3 | 3.0 |
|  |  |  | CPIJ014719 | alaserpin | 202.3 | 2.3 |
|  |  |  | CPIJ014892 | conserved hypothetical protein | 29.5 | 2.4 |
|  |  |  | CPIJ015171 | hypothetical protein | 1.5 | - |
|  |  |  | CPIJ015328 | nesprin | 30.5 | 3.0 |
|  |  |  | CPIJ015823 | conserved hypothetical protein | 1.3 | - |
|  |  |  | CPIJ015857 | NADH dehydrogenase | 489.7 | 2.4 |
|  |  |  | CPIJ016012 | tryptase-2 | 190.8 | 2.2 |
|  |  |  | CPIJ016374 | conserved hypothetical protein | 32.9 | 3.6 |
|  |  |  | CPIJ016375 | conserved hypothetical protein | 3.7 | - |
|  |  |  | CPIJ016440 | dihydroceramide delta (4)-desaturase | 18.9 | 4.1 |
|  |  |  | CPIJ016762 | conserved hypothetical protein | 1.0 | - |
|  |  |  | CPIJ016914 | hypothetical protein | 1.6 | - |
|  |  |  | CPIJ017076 | conserved hypothetical protein | 34.4 | 3.0 |
|  |  |  | CPIJ017149 | l(2) long form | 57.7 | 2.9 |
|  |  |  | CPIJ017150 | l(2) long form | 30.0 | 2.6 |
|  |  |  | CPIJ017621 | conserved hypothetical protein | 1.1 | - |
|  |  |  | CPIJ017717 | conserved hypothetical protein | 27.3 | 4.1 |
|  |  |  | CPIJ017730 | hypothetical protein | 39.8 | 9.2 |
|  |  |  | CPIJ018002 | conserved hypothetical protein | 33.5 | 2.1 |
|  |  |  | CPIJ018092 | ryanodine receptor 3, brain | 9.5 | 2.5 |
|  |  |  | CPIJ018231 | carboxylesterase | 81.7 | 3.2 |
|  |  |  | CPIJ018233 | carboxylesterase | 5766.3 | 3.5 |
|  |  |  | CPIJ018544 | conserved hypothetical protein | 5.8 | 20.3 |
|  |  |  | CPIJ018724 | conserved hypothetical protein | 70.8 | 2.5 |
|  |  |  | CPIJ018791 | conserved hypothetical protein | 60.3 | 2.9 |
|  |  |  | CPIJ018967 | conserved hypothetical protein | 6.6 | 3.5 |
|  |  |  | CPIJ018988 | phosphatidylinositol glycan, class c | 1.5 | - |
|  |  |  | CPIJ019007 | polyserase-2 | 10.4 | 2.9 |
|  |  |  | CPIJ019029 | metalloproteinase | 181.5 | 2.6 |
|  |  |  | CPIJ019577 | alpha-actinin | 361.7 | 2.9 |
| Other | Unknown function | Bactericidal permeability-increasing protein, BPI | CPIJ020308 | conserved hypothetical protein | 1742.1 | 3.1 |
|  |  | E set domains | CPIJ002744 | conserved hypothetical protein | 944.9 | 51.0 |
|  |  |  | CPIJ018825 | larval serum protein 1 beta chain | 1061.3 | 9.4 |
|  |  | Ligand-binding domain in the NO signalling and Golgi transport | CPIJ004088 | guanylyl cyclase receptor | 7.5 | 3.2 |
|  | Viral proteins | Retrovirus zinc finger-like domains | CPIJ006202 | conserved hypothetical protein | 10.0 | 3.4 |
| Regulation | DNA-binding | C2H2 and C2HC zinc fingers | CPIJ004716 | zinc finger protein 266 | 3.8 | 5.5 |
|  |  |  | CPIJ009633 | conserved hypothetical protein | 7.6 | 3.2 |
|  |  |  | CPIJ011598 | zinc finger protein | 2.1 | 3.5 |
|  |  |  | CPIJ015936 | hypothetical protein | 2.4 | - |
|  | Receptor activity | Chemosensory protein Csp2 | CPIJ002617 | chemosensory protein 1 | 865.9 | 2.3 |
|  | Signal transduction | Growth factor receptor domain | CPIJ005087 | cell wall cysteine-rich protein | 16.2 | 2.8 |
|  | DNA-binding | HLH, helix-loop-helix DNA-binding domain | CPIJ018167 | sterol regulatory element-binding protein 1 | 39.1 | 2.1 |
|  |  | Homeodomain-like | CPIJ002050 | homeobox protein | 21.7 | 2.9 |
|  | Signal transduction | Insect pheromone/odorant-binding proteins | CPIJ001872 | Odorant-binding protein 56a | 24.7 | 5.0 |
|  |  |  | CPIJ002108 | odorant-binding protein | 17.2 | 2.7 |
|  |  |  | CPIJ002111 | Odorant-binding protein 50d | 28.8 | 4.0 |
|  |  |  | CPIJ004145 | predicted protein | 1.9 | - |
|  |  |  | CPIJ009038 | odorant binding protein 1 | 1.8 | - |
|  |  | Nicotinic receptor ligand binding domain-like | CPIJ002436 | neuronal acetylcholine receptor subunit alpha-2 | 30.1 | 2.5 |
|  |  | Nuclear receptor ligand-binding domain | CPIJ010249 | retinoid X receptor alpha | 24.6 | 2.5 |
|  |  | PDZ domain-like | CPIJ015336 | Dlg5 protein | 4.0 | 2.6 |
|  | Kinases/phos-phatases | Protein kinase-like (PK-like) | CPIJ010307 | conserved hypothetical protein | 31.9 | 20.3 |
|  |  |  | CPIJ010319 | Juvenile hormone-inducible protein | 12.6 | 2.7 |
|  |  |  | CPIJ010324 | conserved hypothetical protein | 19.5 | 4.2 |
|  |  |  | CPIJ012702 | conserved hypothetical protein | 32.8 | 2.3 |
|  |  |  | CPIJ012763 | 3-phosphoinositide-dependent protein kinase 1 | 6.3 | 2.7 |
|  | RNA binding, m/tr | RNA-binding domain, RBD | CPIJ001827 | conserved hypothetical protein | 1.5 | 7.8 |
|  | Signal transduction | TRAF domain-like | CPIJ001427 | conserved hypothetical protein | 28.3 | 2.3 |
|  |  |  | CPIJ006152 | conserved hypothetical protein | 25.9 | 2.7 |

^†^Differentially expressed genes represent those genes that differed in their expression level (FPKM) in HAmCq^G8^ by more than two fold when compared to the parental strain HAmCq^G0^.

^*^SCOP general and detailed functions using the predicted *Cx. quinquefasciatus* annotation information available at the Superfamily website (version 1.75) supfam.cs.bris.ac.uk/SUPERFAMILY/index.html

^**^*Culex quinquefasciatus* genome, Johannesburg strain CpipJ1.2, June 2008; http://cquinquefasciatus.vectorbase.org/

^#^ Relative FPKM is the ratio of the FPKM value of HAmCq^G8^ divided by the FPKM value of HAmCq^G0^

^£^ Annotations for cytochrome P450 genes were taken from the most current annotation based on: Nelson, DR (2009) The Cytochrome P450 Homepage. Human Genomics 4, 59-65: http://drnelson.uthsc.edu/CytochromeP450.html

^§^NONA: Not annotated
