# Supplementary material for: The Transcriptome Profile of the Mosquito Culex quinquefasciatus following Permethrin Selection
Source: PLoS One. 2012 Oct 5;7(10):e47163. doi: 10.1371/journal.pone.0047163 (PMC3465273; doi:10.1371/journal.pone.0047163)
Supplement: Table S4 — List of genes downregulated by at least two-fold in HAmCqG8 when compared to HAmCqG0. (DOC) [file pone.0047163.s004.doc]

Table S4. List of genes downregulated by at least two-fold in HAmCq^G8^ when compared to HAmCq^G0^.

| **Level of SCOP^*^ classification** | | | **Downregulated genes** | |
| --- | --- | --- | --- | --- |
| **General function** | **Detailed function** | **Superfamily** | **Gene Accession^**^** | **Vectorbase annotation^‡^** |
| Extra-cellular processes | Blood clotting | Fibrinogen C-terminal domain-like | CPIJ000868 | conserved hypothetical protein |
|  |  |  | CPIJ001260 | fibrinogen and fibronectin |
|  |  |  | CPIJ001908 | fibrinogen and fibronectin |
|  |  |  | CPIJ006392 | fibrinogen and fibronectin |
|  |  |  | CPIJ012891 | scabrous protein |
|  |  |  | CPIJ013288 | fibrinogen and fibronectin |
|  |  |  | CPIJ014543 | conserved hypothetical protein |
|  |  |  | CPIJ014544 | conserved hypothetical protein |
|  |  |  | CPIJ017657 | salivary secreted angiopoietin |
|  |  |  | CPIJ017837 | angiopoietin 2 |
|  |  |  | CPIJ017877 | fibrinogen and fibronectin |
|  |  |  | CPIJ018745 | zinc finger protein |
|  |  |  | CPIJ018858 | fibrinogen and fibronectin |
|  | Cell adhesion | alpha-catenin/vinculin-like | CPIJ005773 | actin binding protein |
|  |  | C-type lectin-like | CPIJ000443 | galactose-specific C-type lectin |
|  |  |  | CPIJ001323 | galactose-specific C-type lectin |
|  |  |  | CPIJ004607 | conserved hypothetical protein |
|  |  |  | CPIJ005619 | collagen alpha 1 |
|  |  |  | CPIJ005984 | conserved hypothetical protein |
|  |  |  | CPIJ005987 | conserved hypothetical protein |
|  |  |  | CPIJ006092 | conserved hypothetical protein |
|  |  |  | CPIJ012139 | conserved hypothetical protein |
|  |  |  | CPIJ015742 | conserved hypothetical protein |
|  |  |  | CPIJ018154 | conserved hypothetical protein |
|  |  | Cadherin-like | CPIJ017350 | conserved hypothetical protein |
|  |  |  | CPIJ018739 | conserved hypothetical protein |
|  |  |  | CPIJ018999 | conserved hypothetical protein |
|  |  | EGF/Laminin | CPIJ017322 | conserved hypothetical protein |
|  |  |  | CPIJ004682 | laminin subunit beta-1 |
|  |  |  | CPIJ005569 | neurogenic locus notch |
|  |  |  | CPIJ005673 | conserved hypothetical protein |
|  |  |  | CPIJ009613 | serrate protein |
|  |  |  | CPIJ009614 | serrate protein |
|  |  |  | CPIJ009802 | conserved hypothetical protein |
|  |  |  | CPIJ011761 | conserved hypothetical protein |
|  |  |  | CPIJ015361 | conserved hypothetical protein |
|  |  |  | CPIJ019124 | conserved hypothetical protein |
|  |  | FAS1 domain | CPIJ003831 | conserved hypothetical protein |
|  |  |  | CPIJ004689 | conserved hypothetical protein |
|  |  | Fibronectin type III | CPIJ002017 | cell adhesion molecule |
|  |  |  | CPIJ003912 | conserved hypothetical protein |
|  |  |  | CPIJ004128 | conserved hypothetical protein |
|  |  |  | CPIJ005092 | conserved hypothetical protein |
|  |  |  | CPIJ006893 | myosin light chain kinase |
|  |  |  | CPIJ008112 | cell adhesion molecule |
|  |  |  | CPIJ009217 | roundabout 1 |
|  |  |  | CPIJ014383 | factor for adipocyte differentiation |
|  |  |  | CPIJ014908 | host cell factor C1 |
|  |  |  | CPIJ018836 | conserved hypothetical protein |
|  |  |  | CPIJ020251 | conserved hypothetical protein |
|  |  | FnI-like domain | CPIJ005093 | conserved hypothetical protein |
|  |  |  | CPIJ013285 | conserved hypothetical protein |
|  |  |  | CPIJ015976 | conserved hypothetical protein |
|  |  |  | CPIJ020078 | conserved hypothetical protein |
|  |  | Immunoglobulin | CPIJ003910 | conserved hypothetical protein |
|  |  |  | CPIJ004966 | conserved hypothetical protein |
|  |  |  | CPIJ007299 | beat protein |
|  |  |  | CPIJ009084 | conserved hypothetical protein |
|  |  |  | CPIJ009950 | conserved hypothetical protein |
|  |  |  | CPIJ012166 | conserved hypothetical protein |
|  |  |  | CPIJ012499 | conserved hypothetical protein |
|  |  |  | CPIJ014508 | conserved hypothetical protein |
|  |  |  | CPIJ015510 | conserved hypothetical protein |
|  |  |  | CPIJ017558 | defective proboscis extension response |
|  |  |  | CPIJ018083 | conserved hypothetical protein |
|  |  | Integrin alpha N-terminal domain | CPIJ005252 | T-cell immunomodulatory protein |
|  |  |  | CPIJ017320 | integrin alpha-PS2 |
|  |  | RNI-like | CPIJ002568 | f-box/leucine rich repeat protein |
|  |  |  | CPIJ003404 | conserved hypothetical protein |
|  |  |  | CPIJ004460 | tubulin-specific chaperone |
|  |  |  | CPIJ014282 | conserved hypothetical protein |
|  |  |  | CPIJ015037 | conserved hypothetical protein |
|  |  |  | CPIJ015883 | f-box/lrr protein, drome |
|  |  |  | CPIJ016145 | predicted protein |
|  |  |  | CPIJ016147 | predicted protein |
|  |  |  | CPIJ016518 | conserved hypothetical protein |
|  |  |  | CPIJ017031 | conserved hypothetical protein |
|  |  |  | CPIJ017284 | conserved hypothetical protein |
|  |  |  | CPIJ019360 | predicted protein |
|  |  | SEA domain | CPIJ011474 | conserved hypothetical protein |
|  |  | Somatomedin B domain | CPIJ000705 | conserved hypothetical protein |
|  |  | Spectrin repeat | CPIJ003238 | conserved hypothetical protein |
|  |  |  | CPIJ006907 | conserved hypothetical protein |
|  |  |  | CPIJ011432 | conserved hypothetical protein |
|  |  |  | CPIJ013591 | conserved hypothetical protein |
|  |  | TSP-1 type 1 repeat | CPIJ000706 | conserved hypothetical protein |
|  |  | vWA-like | CPIJ004522 | 26S proteasome non-ATPase regulatory subunit 4 |
|  |  |  | CPIJ005333 | transport protein sec23 |
|  |  |  | CPIJ006690 | integrin beta-PS |
|  | Immune response | Complement control module/SCR domain | CPIJ005492 | conserved hypothetical protein |
|  |  |  | CPIJ007796 | conserved hypothetical protein |
|  |  |  | CPIJ008865 | conserved hypothetical protein |
|  |  | Tetraspanin | CPIJ013906 | platelet endothelial tetraspan antigen 3 |
|  |  |  | CPIJ017253 | conserved hypothetical protein |
|  |  | TNF-like | CPIJ011491 | conserved hypothetical protein |
|  | Toxins/defense | AhpD-like | CPIJ010665 | P53 regulated pa26 nuclear protein sestrin |
|  |  | omega toxin-like | CPIJ000956 | conserved hypothetical protein |
|  |  | Scorpion toxin-like | CPIJ011918 | conserved hypothetical protein |
|  |  | Snake toxin-like | CPIJ011561 | 14.5 kDa salivary peptide |
|  |  |  | CPIJ011853 | conserved hypothetical protein |
|  |  |  | CPIJ017088 | activin receptor type I |
| General | General | ARM repeat | CPIJ006701 | 26S proteasome non-ATPase regulatory subunit 1 |
|  |  |  | CPIJ000004 | conserved hypothetical protein |
|  |  |  | CPIJ000739 | conserved hypothetical protein |
|  |  |  | CPIJ001288 | conserved hypothetical protein |
|  |  |  | CPIJ001478 | pre-mRNA-splicing factor cwc22 |
|  |  |  | CPIJ004189 | conserved hypothetical protein |
|  |  |  | CPIJ004193 | conserved hypothetical protein |
|  |  |  | CPIJ004613 | importin subunit beta |
|  |  |  | CPIJ004649 | cell differentiation protein rcd1 |
|  |  |  | CPIJ004747 | smaug protein |
|  |  |  | CPIJ004787 | FKBP12-rapamycin complex-associated protein |
|  |  |  | CPIJ004900 | armadillo repeat-containing protein 6 |
|  |  |  | CPIJ006132 | importin alpha |
|  |  |  | CPIJ006316 | conserved hypothetical protein |
|  |  |  | CPIJ006702 | 26S proteasome non-ATPase regulatory subunit 1 |
|  |  |  | CPIJ007322 | conserved hypothetical protein |
|  |  |  | CPIJ009961 | stromal antigen |
|  |  |  | CPIJ010395 | conserved hypothetical protein |
|  |  |  | CPIJ012523 | coatomer subunit gamma |
|  |  |  | CPIJ012812 | thyroid hormone receptor interactor 12 |
|  |  |  | CPIJ013105 | sorting nexin |
|  |  |  | CPIJ013982 | conserved hypothetical protein |
|  |  |  | CPIJ014675 | conserved hypothetical protein |
|  |  |  | CPIJ018110 | conserved hypothetical protein |
|  |  |  | CPIJ018379 | adaptin, alpha/gamma/epsilon |
|  |  |  | CPIJ018684 | conserved hypothetical protein |
|  |  |  | CPIJ019706 | 26S proteasome non-ATPase regulatory subunit 2 |
|  |  | BRCT domain | CPIJ011710 | conserved hypothetical protein |
|  |  | Calponin-homology domain, CH-domain | CPIJ003234 | conserved hypothetical protein |
|  |  |  | CPIJ004533 | microtubule binding protein |
|  |  |  | CPIJ008540 | muscle-specific protein 20 |
|  |  | Cryptochrome/photolyase FAD-binding domain | CPIJ003975 | deoxyribodipyrimidine photo-lyase |
|  |  |  | CPIJ009455 | DNA photolyase |
|  |  |  | CPIJ018859 | cryptochrome 2 |
|  |  | EF-hand | CPIJ001307 | predicted protein |
|  |  |  | CPIJ001896 | conserved hypothetical protein |
|  |  |  | CPIJ002594 | nadph oxidase |
|  |  |  | CPIJ004307 | supercoiling factor |
|  |  |  | CPIJ005099 | voltage-dependent p/q type calcium channel |
|  |  |  | CPIJ008225 | conserved hypothetical protein |
|  |  |  | CPIJ009356 | dynamin-associated protein |
|  |  |  | CPIJ010178 | conserved hypothetical protein |
|  |  |  | CPIJ012251 | troponin C |
|  |  |  | CPIJ015810 | calcium-binding protein E63-1 |
|  |  |  | CPIJ015812 | calcium-binding protein E63-1 |
|  |  | Kelch motif | CPIJ000983 | actin binding protein |
|  |  |  | CPIJ011586 | actin binding protein |
|  |  |  | CPIJ011613 | conserved hypothetical protein |
|  |  |  | CPIJ014909 | host cell factor |
|  |  | L domain-like | CPIJ001272 | leucine-rich transmembrane protein |
|  |  |  | CPIJ001895 | conserved hypothetical protein |
|  |  |  | CPIJ003386 | predicted protein |
|  |  |  | CPIJ003844 | leucine-rich transmembrane protein |
|  |  |  | CPIJ004868 | leucine-rich transmembrane protein |
|  |  |  | CPIJ005354 | conserved hypothetical protein |
|  |  |  | CPIJ006822 | leucine-rich transmembrane protein |
|  |  |  | CPIJ011783 | conserved hypothetical protein |
|  |  |  | CPIJ012832 | conserved hypothetical protein |
|  |  |  | CPIJ013310 | adenylate cyclase |
|  |  |  | CPIJ013854 | conserved hypothetical protein |
|  |  |  | CPIJ015804 | reticulon/nogo receptor |
|  |  |  | CPIJ016693 | ras suppressor protein 1 |
|  |  |  | CPIJ016806 | leucine rich repeat protein |
|  |  |  | CPIJ017602 | conserved hypothetical protein |
|  |  |  | CPIJ018453 | conserved hypothetical protein |
|  |  |  | CPIJ018876 | leucine-rich repeat-containing protein 24 |
|  |  |  | CPIJ019625 | conserved hypothetical protein |
|  |  |  | CPIJ019816 | conserved hypothetical protein |
|  |  | Spermadhesin, CUB domain | CPIJ003741 | conserved hypothetical protein |
|  |  |  | CPIJ015617 | conserved hypothetical protein |
|  |  |  | CPIJ018283 | conserved hypothetical protein |
|  |  | Transthyretin (synonym: prealbumin) | CPIJ014057 | conserved hypothetical protein |
|  |  | Ubiquitin-like | CPIJ002389 | transcription elongation factor B polypeptide 2 |
|  |  |  | CPIJ003604 | conserved hypothetical protein |
|  |  |  | CPIJ007098 | ubiquitin-fold modifier 1 |
|  |  |  | CPIJ011014 | peptidylglycine alpha-amidating monooxygenase COOH-terminal interactor protein-1 |
|  |  |  | CPIJ011765 | conserved hypothetical protein |
|  |  |  | CPIJ014686 | conserved hypothetical protein |
|  |  |  | CPIJ019167 | peptidylglycine alpha-amidating monooxygenase COOH-terminal interactor protein-1 |
|  |  | WD40 repeat-like | CPIJ000633 | conserved hypothetical protein |
|  |  |  | CPIJ001314 | WD repeat protein 46 |
|  |  |  | CPIJ001915 | WD repeat domain 50 |
|  |  |  | CPIJ002199 | vesicle associated protein |
|  |  |  | CPIJ002451 | WD repeat protein 57 |
|  |  |  | CPIJ003096 | WD repeat protein 7 |
|  |  |  | CPIJ003211 | WD repeat protein 51B |
|  |  |  | CPIJ003605 | vacuolar membrane protein pep11 |
|  |  |  | CPIJ004561 | pleiotropic regulator 1 |
|  |  |  | CPIJ005067 | G protein beta subunit |
|  |  |  | CPIJ005486 | autophagy-specific gene 18 |
|  |  |  | CPIJ006095 | conserved hypothetical protein |
|  |  |  | CPIJ006523 | conserved hypothetical protein |
|  |  |  | CPIJ007014 | splicing factor 3B subunit 3 |
|  |  |  | CPIJ007402 | guanine nucleotide-binding protein subunit beta 1 |
|  |  |  | CPIJ009226 | groucho protein |
|  |  |  | CPIJ009392 | nucleoporin Nup43 |
|  |  |  | CPIJ009985 | conserved hypothetical protein |
|  |  |  | CPIJ010477 | conserved hypothetical protein |
|  |  |  | CPIJ010919 | will die slowly |
|  |  |  | CPIJ011032 | conserved hypothetical protein |
|  |  |  | CPIJ011061 | WD repeat protein 51A |
|  |  |  | CPIJ011261 | cell cycle control protein cwf8 |
|  |  |  | CPIJ011322 | mediator complex, 95kD-subunit |
|  |  |  | CPIJ011395 | conserved hypothetical protein |
|  |  |  | CPIJ013687 | conserved hypothetical protein |
|  |  |  | CPIJ013831 | WD repeat-containing protein srw1 |
|  |  |  | CPIJ014207 | elongator complex protein 2 |
|  |  |  | CPIJ014657 | vesicle associated protein |
|  |  |  | CPIJ015003 | predicted protein |
|  |  |  | CPIJ015347 | receptor for activated protein kinase C |
|  |  |  | CPIJ015352 | conserved hypothetical protein |
|  |  |  | CPIJ015911 | conserved hypothetical protein |
|  |  |  | CPIJ017007 | vesicle associated protein |
|  |  |  | CPIJ017534 | serine-threonine kinase receptor-associated protein |
|  |  |  | CPIJ017825 | wd-repeat protein |
|  |  |  | CPIJ018264 | will die slowly |
|  |  |  | CPIJ018766 | WD repeat domain phosphoinositide-interacting protein 2 |
|  |  |  | CPIJ019808 | receptor for activated protein kinase C |
|  |  |  | CPIJ020009 | wd-repeat protein |
|  | Ion binding | Amyloid beta a4 protein copper binding domain (domain 2) | CPIJ008559 | conserved hypothetical protein |
|  |  | ArfGap/RecO-like zinc finger | CPIJ002305 | arf GTPase-activating protein |
|  |  |  | CPIJ008955 | arf GTPase-activating protein |
|  |  |  | CPIJ010923 | conserved hypothetical protein |
|  |  | B-box zinc-binding domain | CPIJ015175 | predicted protein |
|  | Ligand binding | GYF domain | CPIJ001658 | conserved hypothetical protein |
|  |  |  | CPIJ010290 | CD2 antigen cytoplasmic tail-binding protein 2 |
|  | Protein interaction | Ankyrin repeat | CPIJ000121 | conserved hypothetical protein |
|  |  |  | CPIJ000884 | DNA-binding protein rfxank |
|  |  |  | CPIJ001854 | conserved hypothetical protein |
|  |  |  | CPIJ002709 | conserved hypothetical protein |
|  |  |  | CPIJ004774 | developmental protein cactus |
|  |  |  | CPIJ004983 | ga binding protein beta chain |
|  |  |  | CPIJ006925 | conserved hypothetical protein |
|  |  |  | CPIJ006926 | phosphatase 1 regulatory subunit 12b |
|  |  |  | CPIJ009794 | conserved hypothetical protein |
|  |  |  | CPIJ010734 | sex-determining protein fem-1 |
|  |  |  | CPIJ013520 | conserved hypothetical protein |
|  |  |  | CPIJ014438 | sex-determining protein fem-1 |
|  |  |  | CPIJ015938 | conserved hypothetical protein |
|  |  |  | CPIJ017182 | forked protein |
|  |  |  | CPIJ018599 | transient receptor potential channel |
|  |  |  | CPIJ018744 | ankyrin 2,3/unc44 |
|  |  | BAG domain | CPIJ002809 | conserved hypothetical protein |
|  |  | BAR/IMD domain-like | CPIJ002500 | conserved hypothetical protein |
|  |  |  | CPIJ007107 | insulin receptor tyrosine kinase substrate |
|  |  |  | CPIJ011282 | islet cell autoantigen 1 |
|  |  |  | CPIJ017697 | endophilin b |
|  |  | Dimerization-anchoring domain of cAMP-dependent PK regulatory subunit | CPIJ004553 | predicted protein |
|  |  |  | CPIJ015945 | conserved hypothetical protein |
|  |  | F-box domain | CPIJ000909 | conserved hypothetical protein |
|  |  |  | CPIJ006380 | conserved hypothetical protein |
|  |  |  | CPIJ017453 | transmembrane protein 183 |
|  |  | Hemopexin-like domain | CPIJ001428 | matrix metalloproteinase |
|  |  |  | CPIJ010856 | matrix metalloproteinase |
|  |  | HIV integrase-binding domain | CPIJ003093 | hepatoma-derived GF |
|  |  | IP3 receptor type 1 binding core, domain 2 | CPIJ012217 | inositol 1,4,5-trisphosphate receptor |
|  |  | POZ domain | CPIJ001236 | conserved hypothetical protein |
|  |  |  | CPIJ001455 | ankyrin repeat and BTB/POZ domain-containing protein 2 |
|  |  |  | CPIJ001669 | BTB/POZ domain-containing protein 7 |
|  |  |  | CPIJ003990 | conserved hypothetical protein |
|  |  |  | CPIJ005014 | BTB/POZ and Kelch domain-containing protein |
|  |  |  | CPIJ005696 | serine-enriched protein |
|  |  |  | CPIJ007217 | speckle-type poz protein |
|  |  |  | CPIJ007547 | conserved hypothetical protein |
|  |  |  | CPIJ008271 | conserved hypothetical protein |
|  |  |  | CPIJ009395 | speckle-type poz protein |
|  |  |  | CPIJ009648 | conserved hypothetical protein |
|  |  |  | CPIJ012486 | conserved hypothetical protein |
|  |  |  | CPIJ012629 | conserved hypothetical protein |
|  |  |  | CPIJ013200 | conserved hypothetical protein |
|  |  |  | CPIJ013368 | microtubule binding protein |
|  |  |  | CPIJ013627 | conserved hypothetical protein |
|  |  |  | CPIJ016082 | conserved hypothetical protein |
|  |  |  | CPIJ017663 | leucine-zipper-like transcriptional regulator 1 |
|  |  |  | CPIJ018109 | conserved hypothetical protein |
|  |  |  | CPIJ018129 | conserved hypothetical protein |
|  |  | SNARE-like | CPIJ009504 | clathrin coat assembly protein AP17 |
|  |  |  | CPIJ013281 | conserved hypothetical protein |
|  |  |  | CPIJ017540 | coatomer subunit delta |
|  |  | SWIB/MDM2 domain | CPIJ019141 | brg-1 associated factor |
|  |  |  | CPIJ019147 | brg-1 associated factor |
|  |  | TPR-like | CPIJ001441 | eukaryotic translation initiation factor 3 subunit |
|  |  |  | CPIJ002036 | transmembrane and TPR repeat-containing protein |
|  |  |  | CPIJ003799 | suppressor of forked |
|  |  |  | CPIJ004405 | conserved hypothetical protein |
|  |  |  | CPIJ005156 | conserved hypothetical protein |
|  |  |  | CPIJ008245 | conserved hypothetical protein |
|  |  |  | CPIJ008355 | transmembrane protein 1/tmem1b |
|  |  |  | CPIJ010925 | heat shock protein 70 |
|  |  |  | CPIJ011544 | tetratricopeptide repeat domain 21B |
|  |  |  | CPIJ017131 | prolyl 4-hydroxylase subunit alpha-1 |
|  |  |  | CPIJ019076 | tetratricopeptide repeat protein 15 |
|  |  | UBA-like | CPIJ006055 | conserved hypothetical protein |
|  |  |  | CPIJ011493 | conserved hypothetical protein |
|  |  |  | CPIJ012192 | conserved hypothetical protein |
|  |  | Vasodilator-stimulated phosphoprotein, VASP, tetramerisation domain | CPIJ004707 | vasodilator-stimulated phosphoprotein |
|  |  | WW domain | CPIJ000289 | conserved hypothetical protein |
|  |  |  | CPIJ000291 | conserved hypothetical protein |
|  |  |  | CPIJ004712 | conserved hypothetical protein |
|  |  |  | CPIJ013704 | conserved hypothetical protein |
|  |  |  | CPIJ014839 | conserved hypothetical protein |
|  |  |  | CPIJ019077 | conserved hypothetical protein |
|  |  | FAD/NAD(P)-binding domain | CPIJ001215 | alcohol dehydrogenase |
|  |  |  | CPIJ001367 | glucose dehydrogenase |
|  |  |  | CPIJ002196 | lysine-specific histone demethylase |
|  |  |  | CPIJ002643 | CDNA sequence |
|  |  |  | CPIJ005552 | thioredoxin reductase 1, mitochondrial |
|  |  |  | CPIJ007619 | glucose dehydrogenase |
|  |  |  | CPIJ007625 | alcohol dehydrogenase |
|  |  |  | CPIJ009583 | glucose dehydrogenase |
|  |  |  | CPIJ010620 | conserved hypothetical protein |
|  |  |  | CPIJ010669 | rab protein geranylgeranyltransferase component A 1 |
|  |  |  | CPIJ013724 | dimethylaniline monooxygenase |
|  |  |  | CPIJ013725 | dimethylaniline monooxygenase |
|  |  |  | CPIJ017482 | choline dehydrogenase |
|  |  |  | CPIJ017488 | glucose dehydrogenase |
|  |  |  | CPIJ017490 | glucose dehydrogenase |
|  |  |  | CPIJ017491 | glucose dehydrogenase |
|  |  | Glutathione S-transferase (GST), C-terminal domain | CPIJ002660 | glutathione-s-transferase theta, gst |
|  |  |  | CPIJ002680 | glutathione S-transferase |
|  |  |  | CPIJ003988 | prostaglandin E synthase 2 |
|  |  |  | CPIJ014051 | glutathione-s-transferase theta, gst |
|  |  |  | CPIJ014053 | glutathione-s-transferase theta, gst |
|  |  |  | CPIJ018524 | prostaglandin E synthase 2 |
|  |  |  | CPIJ018633 | glutathione-s-transferase theta |
|  |  | NAD(P)-binding Rossmann-fold domains | CPIJ016763 | short-chain dehydrogenase |
|  |  |  | CPIJ000400 | 3-hydroxyisobutyrate dehydrogenase |
|  |  |  | CPIJ000841 | dimeric dihydrodiol dehydrogenase |
|  |  |  | CPIJ003056 | hydroxysteroid dehydrogenase |
|  |  |  | CPIJ003801 | NADP-dependent leukotriene B4 12-hydroxydehydrogenase |
|  |  |  | CPIJ003837 | short-chain dehydrogenase |
|  |  |  | CPIJ004391 | fatty acyl-CoA reductase 1 |
|  |  |  | CPIJ004392 | fatty acyl-CoA reductase 2 |
|  |  |  | CPIJ005892 | conserved hypothetical protein |
|  |  |  | CPIJ006479 | 3-hydroxyacyl-coa dehyrogenase |
|  |  |  | CPIJ007225 | 3-ketodihydrosphingosine reductase |
|  |  |  | CPIJ007244 | fatty acyl-CoA reductase 1 |
|  |  |  | CPIJ007245 | fatty acyl-CoA reductase 1 |
|  |  |  | CPIJ011767 | short-chain dehydrogenase |
|  |  |  | CPIJ013219 | 3-hydroxybutyrate dehydrogenase type 2 |
|  |  |  | CPIJ014059 | NADP-dependent leukotriene B4 12-hydroxydehydrogenase |
|  |  |  | CPIJ014121 | short-chain dehydrogenase |
|  |  |  | CPIJ014122 | dehydrogenase/reductase SDR family member 8 |
|  |  |  | CPIJ014580 | dimeric dihydrodiol dehydrogenase |
|  |  |  | CPIJ015671 | glyoxylate reductase/hydroxypyruvate reductase |
|  |  |  | CPIJ015685 | 3-oxoacyl-[acyl-carrier-protein] reductase |
|  |  |  | CPIJ016656 | short-chain dehydrogenase |
|  |  |  | CPIJ016657 | short-chain dehydrogenase |
|  |  |  | CPIJ016719 | alcohol dehydrogenase 1 |
|  |  |  | CPIJ016777 | hydroxyacyl-coenzyme A dehydrogenase, mitochondrial |
|  |  |  | CPIJ017297 | quinone oxidoreductase |
|  |  |  | CPIJ017713 | short-chain dehydrogenase |
|  |  |  | CPIJ018318 | short-chain dehydrogenase |
|  |  |  | CPIJ019137 | dihydropteridine reductase |
|  |  |  | CPIJ019281 | conserved hypothetical protein |
|  |  |  | CPIJ019362 | dehydrogenase/reductase SDR family member 8 |
|  |  |  | CPIJ019941 | conserved hypothetical protein |
|  |  |  | CPIJ019942 | conserved hypothetical protein |
|  |  |  | CPIJ020005 | UDP-glucuronic acid decarboxylase 1 |
|  |  | Nucleotide-binding domain | CPIJ002817 | d-amino acid oxidase |
|  |  |  | CPIJ007272 | d-amino acid oxidase |
|  |  |  | CPIJ007273 | d-amino acid oxidase |
|  |  | Obg GTP-binding protein N-terminal domain | CPIJ005917 | Spo0B-associated GTP-binding protein |
|  |  | P-loop containing nucleoside triphosphate hydrolases | CPIJ014210 | conserved hypothetical protein |
|  |  |  | CPIJ000310 | heparan sulfate 2-o-sulfotransferase |
|  |  |  | CPIJ000320 | conserved hypothetical protein |
|  |  |  | CPIJ000874 | carbohydrate sulfotransferase |
|  |  |  | CPIJ000964 | chromosome-associated kinesin KIF4A |
|  |  |  | CPIJ001058 | ADP-ribosylation factor |
|  |  |  | CPIJ001311 | multidrug resistance-associated protein 2 |
|  |  |  | CPIJ001383 | kinesin-like protein KIF3A |
|  |  |  | CPIJ001540 | abc transporter |
|  |  |  | CPIJ001695 | conserved hypothetical protein |
|  |  |  | CPIJ001702 | conserved hypothetical protein |
|  |  |  | CPIJ001756 | conserved hypothetical protein |
|  |  |  | CPIJ001842 | translation initiation factor IF-2, mitochondrial |
|  |  |  | CPIJ001988 | ATP-dependent RNA helicase A |
|  |  |  | CPIJ002335 | ATP-dependent RNA helicase DDX51 |
|  |  |  | CPIJ003150 | vesicular-fusion protein Nsf1 |
|  |  |  | CPIJ003814 | cell cycle checkpoint protein rad17 |
|  |  |  | CPIJ003934 | RNA helicase |
|  |  |  | CPIJ003935 | ATP-dependent RNA helicase p62 |
|  |  |  | CPIJ004665 | chromodomain helicase-DNA-binding protein 3 |
|  |  |  | CPIJ004980 | ATP-binding cassette sub-family A member 3 |
|  |  |  | CPIJ005169 | ras-related protein Rab-10 |
|  |  |  | CPIJ005172 | GTP-binding protein |
|  |  |  | CPIJ005340 | ATP-binding cassette transporter |
|  |  |  | CPIJ005341 | abc transporter |
|  |  |  | CPIJ005366 | GTP-binding protein yptV1 |
|  |  |  | CPIJ005545 | conserved hypothetical protein |
|  |  |  | CPIJ007064 | conserved hypothetical protein |
|  |  |  | CPIJ007231 | translation elongation factor |
|  |  |  | CPIJ007588 | conserved hypothetical protein |
|  |  |  | CPIJ007795 | guanylate kinase |
|  |  |  | CPIJ007814 | myosin iii |
|  |  |  | CPIJ007889 | abc transporter |
|  |  |  | CPIJ008104 | ras-related protein Rab-39B |
|  |  |  | CPIJ008284 | canalicular multispecific organic anion transporter 1 |
|  |  |  | CPIJ008677 | conserved hypothetical protein |
|  |  |  | CPIJ008800 | ATP-dependent protease La |
|  |  |  | CPIJ008893 | conserved hypothetical protein |
|  |  |  | CPIJ008983 | ATP-dependent RNA helicase DBP8 |
|  |  |  | CPIJ009005 | ATP-dependent DNA helicase MER3 |
|  |  |  | CPIJ009065 | ras-related protein Rab-9 |
|  |  |  | CPIJ009089 | ras-related protein Rab-7 |
|  |  |  | CPIJ009531 | conserved hypothetical protein |
|  |  |  | CPIJ009998 | transcriptional regulator ATRX |
|  |  |  | CPIJ010194 | ras-related protein |
|  |  |  | CPIJ010818 | GTP-binding protein alpha subunit, gna |
|  |  |  | CPIJ010888 | origin recognition complex subunit 1 |
|  |  |  | CPIJ010998 | werner helicase interacting protein |
|  |  |  | CPIJ011002 | bile salt sulfotransferase 1 |
|  |  |  | CPIJ011328 | GTP:AMP phosphotransferase mitochondrial |
|  |  |  | CPIJ011521 | mitochondrial 28S ribosomal protein S29 |
|  |  |  | CPIJ011567 | conserved hypothetical protein |
|  |  |  | CPIJ011830 | serine protease |
|  |  |  | CPIJ012284 | abc transporter |
|  |  |  | CPIJ012364 | abc transporter |
|  |  |  | CPIJ012365 | ATP-binding cassette sub-family G member 4 |
|  |  |  | CPIJ012510 | ATP-dependent RNA helicase DDX24 |
|  |  |  | CPIJ012512 | ATP-dependent RNA helicase p62 |
|  |  |  | CPIJ012614 | sulfotransferase 1A1 |
|  |  |  | CPIJ012621 | ATP-dependent RNA helicase Ddx1 |
|  |  |  | CPIJ013250 | conserved hypothetical protein |
|  |  |  | CPIJ013393 | mitochondrial chaperone BCS1 |
|  |  |  | CPIJ013525 | conserved hypothetical protein |
|  |  |  | CPIJ013876 | DNA polymerase theta |
|  |  |  | CPIJ014038 | DEAD-box ATP-dependent RNA helicase 57 |
|  |  |  | CPIJ014142 | nucleotide-binding protein 1 |
|  |  |  | CPIJ014150 | ATP-binding cassette sub-family F member 3 |
|  |  |  | CPIJ014305 | conserved hypothetical protein |
|  |  |  | CPIJ014361 | conserved hypothetical protein |
|  |  |  | CPIJ014443 | ATP-binding cassette sub-family G member 4 |
|  |  |  | CPIJ014693 | transcriptional regulator ATRX |
|  |  |  | CPIJ014902 | CDC42 |
|  |  |  | CPIJ015682 | translation initiation factor if-2 |
|  |  |  | CPIJ015769 | myosin vi |
|  |  |  | CPIJ015845 | elongation factor tu |
|  |  |  | CPIJ015898 | conserved hypothetical protein |
|  |  |  | CPIJ016097 | peroxisomal membrane protein 70 abcd3 |
|  |  |  | CPIJ016664 | CTP synthase |
|  |  |  | CPIJ016808 | ribosome biogenesis protein |
|  |  |  | CPIJ017203 | conserved hypothetical protein |
|  |  |  | CPIJ017338 | DEAD box ATP-dependent RNA helicase |
|  |  |  | CPIJ017393 | conserved hypothetical protein |
|  |  |  | CPIJ017570 | myosin IB heavy chain |
|  |  |  | CPIJ017886 | ATP-dependent RNA helicase |
|  |  |  | CPIJ018454 | chromosome-associated kinesin KIF4A |
|  |  |  | CPIJ018540 | hypothetical protein |
|  |  |  | CPIJ019196 | DEAD box ATP-dependent RNA helicase |
|  |  |  | CPIJ019594 | chromosome transmission fidelity protein 18 |
|  |  |  | CPIJ019631 | kinesin heavy chain |
|  |  |  | CPIJ019640 | conserved hypothetical protein |
|  |  | PEBP-like | CPIJ003429 | brother of ft and tfl1 |
|  |  |  | CPIJ008654 | phosphatidylethanolamine-binding protein |
| Information | Chromatin structure | NAP-like | CPIJ015455 | nucleosome assembly protein |
|  |  |  | CPIJ015773 | nucleosome assembly protein |
|  | DNA replication/repair | Chromo domain-like | CPIJ007340 | conserved hypothetical protein |
|  |  |  | CPIJ014352 | conserved hypothetical protein |
|  |  |  | CPIJ019929 | conserved hypothetical protein |
|  |  | DNA polymerase III clamp loader subunits, C-terminal domain | CPIJ017857 | ATPase WRNIP1 |
|  |  | DNA/RNA polymerases | CPIJ007351 | terminal deoxycytidyl transferase rev1 |
|  |  |  | CPIJ012266 | DNA polymerase subunit gamma 1, mitochondrial |
|  |  |  | CPIJ015260 | DNA polymerase alpha catalytic subunit |
|  |  | DNase I-like | CPIJ006698 | type I inositol-1,4,5-trisphosphate 5-phosphatase |
|  |  |  | CPIJ008163 | skeletal muscle/kidney enriched inositol 5-phosphatase |
|  |  |  | CPIJ012006 | conserved hypothetical protein |
|  |  |  | CPIJ019984 | sphingomyelin phosphodiesterase 2 |
|  |  | FYVE/PHD zinc finger | CPIJ001881 | conserved hypothetical protein |
|  |  |  | CPIJ002025 | conserved hypothetical protein |
|  |  |  | CPIJ004116 | zinc finger FYVE domain-containing protein 28 |
|  |  |  | CPIJ006170 | CpG-binding protein |
|  |  |  | CPIJ009232 | conserved hypothetical protein |
|  |  |  | CPIJ009396 | inhibitor of growth protein 3 |
|  |  |  | CPIJ011285 | fetal alzheimer antigen, falz |
|  |  |  | CPIJ013376 | phd finger protein |
|  |  |  | CPIJ013847 | conserved hypothetical protein |
|  |  |  | CPIJ014289 | conserved hypothetical protein |
|  |  |  | CPIJ015635 | conserved hypothetical protein |
|  |  |  | CPIJ016701 | inhibitor of growth protein 1 |
|  |  | His-Me finger endonucleases | CPIJ002289 | deoxyribonuclease I |
|  |  |  | CPIJ006433 | caspase-activated nuclease |
|  |  | HRDC-like | CPIJ009943 | conserved hypothetical protein |
|  |  | Nucleic acid-binding proteins | CPIJ003130 | replication factor A, 14kD-subunit |
|  |  |  | CPIJ004582 | mitochondrial ribosomal protein S17 |
|  |  |  | CPIJ005206 | DNA-directed RNA polymeraseI |
|  |  |  | CPIJ005868 | multisynthetase complex auxiliary component p43 |
|  |  |  | CPIJ006691 | insect replication protein a |
|  |  |  | CPIJ008535 | conserved hypothetical protein |
|  |  |  | CPIJ019290 | DNA ligase 4 |
|  |  | Nudix | CPIJ014785 | mitochondrial ribosomal protein, L46 |
|  |  | Restriction endonuclease-like | CPIJ019123 | conserved hypothetical protein |
|  |  | RING/U-box | CPIJ015192 | conserved hypothetical protein |
|  |  |  | CPIJ001165 | ubiquitin conjugation factor E4 A |
|  |  |  | CPIJ001468 | RING-box protein 1a |
|  |  |  | CPIJ003711 | E3 ubiquitin-protein ligase MARCH6 |
|  |  |  | CPIJ004515 | conserved hypothetical protein |
|  |  |  | CPIJ004519 | conserved hypothetical protein |
|  |  |  | CPIJ005021 | vacuolar protein sorting-associated protein 18 |
|  |  |  | CPIJ005056 | conserved hypothetical protein |
|  |  |  | CPIJ005135 | peroxisome assembly factor 1 |
|  |  |  | CPIJ006036 | ring finger protein |
|  |  |  | CPIJ006232 | rolling pebbles |
|  |  |  | CPIJ007831 | conserved hypothetical protein |
|  |  |  | CPIJ010005 | RING finger protein 126-B |
|  |  |  | CPIJ010511 | hypothetical protein |
|  |  |  | CPIJ010577 | conserved hypothetical protein |
|  |  |  | CPIJ010613 | zinc finger protein |
|  |  |  | CPIJ011856 | E3 ubiquitin-protein ligase MARCH5 |
|  |  |  | CPIJ012808 | conserved hypothetical protein |
|  |  |  | CPIJ014265 | zinc and ring finger 2 |
|  |  |  | CPIJ015415 | conserved hypothetical protein |
|  |  |  | CPIJ016043 | autocrine motility factor receptor |
|  |  |  | CPIJ016186 | conserved hypothetical protein |
|  |  |  | CPIJ016790 | zinc finger protein |
|  |  |  | CPIJ017375 | conserved hypothetical protein |
|  |  |  | CPIJ017992 | conserved hypothetical protein |
|  |  | Tudor/PWWP/MBT | CPIJ003208 | predicted protein |
|  |  |  | CPIJ005604 | conserved hypothetical protein |
|  |  |  | CPIJ012664 | conserved hypothetical protein |
|  |  |  | CPIJ014035 | conserved hypothetical protein |
|  | RNA processing | EPT/RTPC-like | CPIJ009234 | RNA 3'-terminal phosphate cyclase |
|  |  | Eukaryotic type KH-domain (KH-domain type I) | CPIJ002909 | far upstream binding protein |
|  |  |  | CPIJ010419 | conserved hypothetical protein |
|  |  |  | CPIJ010634 | conserved hypothetical protein |
|  |  |  | CPIJ011349 | igf2 mRNA binding protein |
|  |  |  | CPIJ014324 | conserved hypothetical protein |
|  |  |  | CPIJ015571 | heterogeneous nuclear ribonucleoprotein |
|  |  |  | CPIJ018107 | zinc finger protein |
|  |  | PAP/OAS1 substrate-binding domain | CPIJ011744 | poly a polymerase |
|  |  |  | CPIJ015488 | sigma DNA polymerase |
|  |  | RNase III domain-like | CPIJ007416 | ribonuclease iii |
|  |  |  | CPIJ008368 | 39S ribosomal protein L44 |
|  |  |  | CPIJ013579 | ribonuclease iii |
|  |  | Translin | CPIJ011089 | translin associated factor x |
|  |  |  | CPIJ011091 | translin associated factor x |
|  | Transcription | beta and beta-prime subunits of DNA dependent RNA-polymerase | CPIJ002457 | DNA-directed RNA polymerase I 135 kDa polypeptide |
|  |  |  | CPIJ018338 | DNA-directed RNA polymerase I largest subunit |
|  |  | CYTH-like phosphatases | CPIJ009805 | conserved hypothetical protein |
|  |  | occludin/ELL-like | CPIJ004404 | conserved hypothetical protein |
|  |  | RBP11-like subunits of RNA polymerase | CPIJ005304 | DNA-directed RNA polymerase II subunit J |
|  |  | TATA-box binding protein-like | CPIJ007600 | TATA-box-binding protein |
|  | Translation | Anticodon-binding domain of a subclass of class I aminoacyl-tRNA synthetases | CPIJ019163 | conserved hypothetical protein |
|  |  | Class II aaRS ABD-related | CPIJ007439 | conserved hypothetical protein |
|  |  |  | CPIJ011030 | conserved hypothetical protein |
|  |  |  | CPIJ017958 | conserved hypothetical protein |
|  |  | EF-Tu/eEF-1alpha/eIF2-gamma C-terminal domain | CPIJ000412 | elongation factor-1 alpha |
|  |  |  | CPIJ005761 | elongation factor 1-alpha |
|  |  |  | CPIJ006444 | elongation factor 1-alpha |
|  |  |  | CPIJ009508 | elongation factor 1 alpha |
|  |  | eIF1-like | CPIJ009942 | density-regulated protein |
|  |  |  | CPIJ013497 | eukaryotic translation initiation factor 1b |
|  |  | eIF4e-like | CPIJ012031 | eukaryotic translation initiation factor 4e type |
|  |  | Elongation factor TFIIS domain 2 | CPIJ013846 | conserved hypothetical protein |
|  |  | Elongation factor Ts (EF-Ts), dimerisation domain | CPIJ004698 | elongation factor ts |
|  |  | L21p-like | CPIJ015639 | 39S ribosomal protein L21, mitochondrial |
|  |  | L27 domain | CPIJ009529 | membrane-associated guanylate kinase |
|  |  | L30e-like | CPIJ005818 | 13 kDa ribonucleoprotein-associated protein |
|  |  | L35p-like | CPIJ019136 | 39S ribosomal protein L35, mitochondrial |
|  |  | L9 N-domain-like | CPIJ013282 | 39S ribosomal protein L9, mitochondrial |
|  |  | Prokaryotic ribosomal protein L17 | CPIJ008935 | 39S ribosomal protein L17, mitochondrial |
|  |  | Prokaryotic type KH domain (KH-domain type II) | CPIJ013400 | ribosomal protein S3 |
|  |  |  | CPIJ016018 | ribosomal protein S3 |
|  |  | Release factor | CPIJ019188 | peptide chain release factor 1 |
|  |  | Ribosomal L11/L12e N-terminal domain | CPIJ013673 | 39S ribosomal protein L11, mitochondrial |
|  |  | Ribosomal protein L16p/L10e | CPIJ012999 | serrate protein |
|  |  |  | CPIJ009291 | 60S ribosomal protein L10 |
|  |  |  | CPIJ017073 | conserved hypothetical protein |
|  |  |  | CPIJ018417 | conserved hypothetical protein |
|  |  |  | CPIJ018847 | 60S ribosomal protein L10 |
|  |  | Ribosomal protein L20 | CPIJ010000 | 39S ribosomal protein L20, mitochondrial |
|  |  | Ribosomal protein L29 (L29p) | CPIJ010482 | hypothetical protein |
|  |  | Ribosomal protein L30p/L7e | CPIJ011578 | mitochondrial ribosomal protein L30 |
|  |  |  | CPIJ012743 | 60S ribosomal protein L7 |
|  |  |  | CPIJ017899 | tetratricopeptide repeat protein, tpr |
|  |  | Ribosomal protein L36 | CPIJ009257 | mitochondrial ribosomal protein L36 |
|  |  | Ribosomal protein S10 | CPIJ011818 | Ded1-like DEAD-box RNA helicase |
|  |  |  | CPIJ017617 | 40S ribosomal protein S20 |
|  |  |  | CPIJ018278 | 40S ribosomal protein S20 |
|  |  |  | CPIJ018511 | 40S ribosomal protein S20 |
|  |  | Ribosomal protein S16 | CPIJ009237 | 28S ribosomal protein S16 |
|  |  | Ribosomal protein S18 | CPIJ002644 | 28S ribosomal protein S18b, mitochondrial |
|  |  | Ribosomal protein S3 C-terminal domain | CPIJ015427 | 40S ribosomal protein S3 |
|  |  | Ribosomal protein S5 domain 2-like | CPIJ008237 | 40S ribosomal protein S2 |
|  |  |  | CPIJ013104 | 40S ribosomal protein S2 |
|  |  |  | CPIJ016812 | 40S ribosomal protein S2 |
|  |  |  | CPIJ018157 | exosome complex exonuclease RRP46 |
|  |  |  | CPIJ018280 | conserved hypothetical protein |
|  |  | Ribosomal protein S6 | CPIJ007632 | mitochondrial 28S ribosomal protein S6 |
|  |  | Ribosomal proteins L15p and L18e | CPIJ012021 | 60S ribosomal protein L18 |
|  |  | Ribosome inactivating proteins (RIP) | CPIJ009211 | conserved hypothetical protein |
|  |  | Second domain of FERM | CPIJ017573 | focal adhesion kinase |
|  |  | Sm-like ribonucleoproteins | CPIJ005588 | small nuclear ribonucleoprotein SM D3 |
|  |  |  | CPIJ006616 | small nuclear ribonucleoprotein-associated protein B |
|  |  |  | CPIJ017424 | small nuclear ribonucleoprotein E |
|  |  | ThrRS/AlaRS common domain | CPIJ015553 | alanyl-tRNA synthetase domain-containing protein 1 |
|  |  | Translation initiation factor 2 beta, aIF2beta, N-terminal domain | CPIJ013860 | eukaryotic translation initiation factor 2 subunit beta |
|  |  | Translation proteins | CPIJ007631 | elongation factor-1 alpha |
|  |  |  | CPIJ009557 | elongation factor 1 alpha |
|  |  |  | CPIJ015678 | conserved hypothetical protein |
|  |  | Translation proteins SH3-like domain | CPIJ008796 | 39S ribosomal protein L2, mitochondrial |
|  |  |  | CPIJ019257 | 39S ribosomal protein L19, mitochondrial |
|  |  | Translational machinery components | CPIJ000042 | 40S ribosomal protein S14 |
|  |  |  | CPIJ000487 | conserved hypothetical protein |
|  |  |  | CPIJ000875 | 40S ribosomal protein S14-A |
|  |  |  | CPIJ002488 | 40S ribosomal protein S14-B |
|  |  |  | CPIJ002871 | 40S ribosomal protein S14 |
|  |  |  | CPIJ003216 | 40S ribosomal protein S14-B |
|  |  |  | CPIJ003943 | 40S ribosomal protein S14 |
|  |  |  | CPIJ006101 | 40S ribosomal protein S14-A |
|  |  |  | CPIJ007174 | 40S ribosomal protein S14 |
|  |  |  | CPIJ008067 | 40S ribosomal protein S14-A |
|  |  |  | CPIJ009287 | conserved hypothetical protein |
|  |  |  | CPIJ010252 | 40S ribosomal protein S14 |
|  |  |  | CPIJ010640 | predicted protein |
|  |  |  | CPIJ011289 | 40S ribosomal protein S14 |
|  |  |  | CPIJ011697 | 40S ribosomal protein S141 |
|  |  |  | CPIJ012110 | 40S ribosomal protein S14-A |
|  |  |  | CPIJ013076 | 40S ribosomal protein S14 |
|  |  |  | CPIJ013802 | 40S ribosomal protein S14-2 |
|  |  |  | CPIJ014959 | 40S ribosomal protein S14-A |
|  |  |  | CPIJ015991 | conserved hypothetical protein |
|  |  |  | CPIJ016597 | 40S ribosomal protein S14 |
|  |  |  | CPIJ017293 | 40S ribosomal protein S14 |
|  |  |  | CPIJ018446 | 40S ribosomal protein S14-B |
|  |  | Zn-binding ribosomal proteins | CPIJ014045 | 39S ribosomal protein L32, mitochondrial |
| Intra-cellular processes | Cell cycle, Apoptosis | CAD & PB1 domains | CPIJ008787 | conserved hypothetical protein |
|  |  |  | CPIJ020175 | conserved hypothetical protein |
|  |  | Cell cycle regulatory proteins | CPIJ006105 | cyclin-dependent kinaseregulatory subunit 1 |
|  |  | Cullin homology domain | CPIJ003980 | anaphase-promoting complex subunit 2 |
|  |  | Cystine-knot cytokines | CPIJ000272 | Sptzle 2 |
|  |  |  | CPIJ000273 | sptzle 2 |
|  |  |  | CPIJ001752 | sptzle 3A |
|  |  |  | CPIJ002281 | sptzle 6 |
|  |  |  | CPIJ012748 | conserved hypothetical protein |
|  |  | DEATH domain | CPIJ010093 | netrin receptor unc5 |
|  |  |  | CPIJ010503 | ankyrin 2,3/unc44 |
|  |  | Inhibitor of apoptosis (IAP) repeat | CPIJ006918 | conserved hypothetical protein |
|  |  | RCC1/BLIP-II | CPIJ011645 | hyperplastic discs protein |
|  |  |  | CPIJ015298 | regulator of chromosome condensation |
|  |  | Rhodanese/Cell cycle control phosphatase | CPIJ001662 | M-phase inducer phosphatase 2 |
|  |  |  | CPIJ013880 | heat shock protein 67B2 |
|  | Cell motility | Actin depolymerizing proteins | CPIJ007823 | glial maturation factor |
|  |  |  | CPIJ015839 | conserved hypothetical protein |
|  |  |  | CPIJ019500 | conserved hypothetical protein |
|  |  | Actin-crosslinking proteins | CPIJ013211 | conserved hypothetical protein |
|  |  | DLC | CPIJ015622 | dynein light chain 1, cytoplasmic-like protein |
|  |  |  | CPIJ015623 | predicted protein |
|  |  | Formin homology 2 domain (FH2 domain) | CPIJ003134 | formin 1,2/cappuccino |
|  |  |  | CPIJ006609 | conserved hypothetical protein |
|  |  |  | CPIJ007323 | formin 3 |
|  |  | I/LWEQ domain | CPIJ004416 | huntingtin interacting protein |
|  |  | Myosin rod fragments | CPIJ014522 | lava lamp protein |
|  |  |  | CPIJ017450 | mushroom body defect protein |
|  |  | Outer arm dynein light chain 1 | CPIJ012834 | conserved hypothetical protein |
|  |  |  | CPIJ015679 | conserved hypothetical protein |
|  |  | Tropomyosin | CPIJ001763 | Ofd1 protein |
|  |  |  | CPIJ005452 | M-type 9 protein |
|  |  | Tubulin nucleotide-binding domain-like | CPIJ003263 | tubulin beta chain |
|  |  |  | CPIJ011550 | tubulin alpha-2 chain |
|  |  |  | CPIJ017383 | tubulin alpha-1 chain |
|  | Ion m/tr | Band 7/SPFH domain | CPIJ001131 | erythrocyte band 7 integral membrane protein |
|  |  | Calcium ATPase, transmembrane domain M | CPIJ001884 | cation-transporting ATPase 13a1 |
|  |  |  | CPIJ005964 | Na+/K+ ATPase alpha subunit |
|  |  |  | CPIJ005965 | conserved hypothetical protein |
|  |  |  | CPIJ013541 | conserved hypothetical protein |
|  |  | Clc chloride channel | CPIJ004937 | chloride channel protein 3 |
|  |  | Cupredoxins | CPIJ010466 | laccase-like multicopper oxidase 1 |
|  |  |  | CPIJ012244 | multicopper oxidase |
|  |  |  | CPIJ012357 | multicopper oxidase |
|  |  |  | CPIJ016802 | laccase-like multicopper oxidase 1 |
|  |  |  | CPIJ020002 | multicopper oxidase |
|  |  | Ferritin-like | CPIJ003762 | coenzyme q10 biosynthesis protein |
|  |  | HMA, heavy metal-associated domain | CPIJ015637 | antioxidant enzyme |
|  |  | MFS general substrate transporter | CPIJ000765 | monocarboxylate transporter |
|  |  |  | CPIJ000988 | sugar transporter |
|  |  |  | CPIJ001970 | organic anion transporter |
|  |  |  | CPIJ001971 | solute carrier organic anion transporter family member 3A1 |
|  |  |  | CPIJ001972 | organic anion transporter |
|  |  |  | CPIJ002124 | hippocampus abundant 1 protein |
|  |  |  | CPIJ002172 | oligopeptide transporter |
|  |  |  | CPIJ003413 | UNC93A protein |
|  |  |  | CPIJ003611 | sugar transporter |
|  |  |  | CPIJ004177 | sodium-dependent phosphate transporter |
|  |  |  | CPIJ005445 | glucose transporter |
|  |  |  | CPIJ006419 | monocarboxylate transporter |
|  |  |  | CPIJ007434 | sodium/phosphate cotransporter |
|  |  |  | CPIJ008117 | monocarboxylate transporter |
|  |  |  | CPIJ008119 | monocarboxylate transporter |
|  |  |  | CPIJ008274 | monocarboxylate transporter 3 |
|  |  |  | CPIJ008344 | sugar transporter |
|  |  |  | CPIJ008424 | integral membrane protein efflux protein efpA |
|  |  |  | CPIJ008947 | sugar transporter |
|  |  |  | CPIJ008948 | sugar transporter |
|  |  |  | CPIJ011542 | synaptic vesicle protein |
|  |  |  | CPIJ011543 | proton-associated sugar transporter A |
|  |  |  | CPIJ012022 | organic cation/carnitine transporter 1 |
|  |  |  | CPIJ014358 | organic cation transporter |
|  |  |  | CPIJ015155 | adenylate cyclase |
|  |  |  | CPIJ015621 | cis,cis-muconate transport protein MucK |
|  |  |  | CPIJ015630 | conserved hypothetical protein |
|  |  |  | CPIJ017354 | adenylate cyclase |
|  |  |  | CPIJ017478 | conserved hypothetical protein |
|  |  |  | CPIJ018460 | mfs transporter |
|  |  |  | CPIJ018461 | mfs transporter |
|  |  |  | CPIJ019487 | organic cation transporter |
|  |  |  | CPIJ019488 | organic cation transporter |
|  |  |  | CPIJ019562 | conserved hypothetical protein |
|  |  |  | CPIJ019820 | sugar transporter |
|  |  | Multidrug resistance efflux transporter EmrE | CPIJ009014 | UDP-N-acetylglucosamine transporter |
|  |  |  | CPIJ010400 | conserved hypothetical protein |
|  |  |  | CPIJ017962 | conserved hypothetical protein |
|  |  | Neurotransmitter-gated ion-channel transmembrane pore | CPIJ006949 | histamine-gated chloride channel subunit |
|  |  |  | CPIJ007636 | conserved hypothetical protein |
|  |  |  | CPIJ010616 | conserved hypothetical protein |
|  |  | Periplasmic binding protein-like II | CPIJ006667 | glutamate receptor |
|  |  |  | CPIJ007989 | porphobilinogen deaminase |
|  |  |  | CPIJ010822 | conserved hypothetical protein |
|  |  | SET domain | CPIJ008357 | conserved hypothetical protein |
|  |  |  | CPIJ010143 | conserved hypothetical protein |
|  |  |  | CPIJ013516 | conserved hypothetical protein |
|  |  |  | CPIJ013517 | conserved hypothetical protein |
|  |  |  | CPIJ013971 | Mll1 protein |
|  |  |  | CPIJ015254 | conserved hypothetical protein |
|  |  |  | CPIJ016652 | histone-lysine N-methyltransferase SETDB1 |
|  |  |  | CPIJ018501 | conserved hypothetical protein |
|  |  | Voltage-gated potassium channels | CPIJ000215 | sodium-and chloride-activated ATP-sensitive potassium channel |
|  |  |  | CPIJ000749 | conserved hypothetical protein |
|  |  |  | CPIJ001990 | conserved hypothetical protein |
|  |  |  | CPIJ005769 | calcium-activated potassium channel alpha chain |
|  |  |  | CPIJ010846 | potassium channel subfamily K member 9 |
|  |  |  | CPIJ017948 | voltage and ligand gated potassium channel |
|  | Phospholipid m/tr | CRAL/TRIO domain | CPIJ001389 | conserved hypothetical protein |
|  |  |  | CPIJ005816 | CRAL/TRIO domain-containing protein |
|  |  |  | CPIJ008515 | cellular retinaldehyde binding protein |
|  |  |  | CPIJ008920 | tyrosine phosphatase n9 |
|  |  |  | CPIJ009578 | CRAL/TRIO domain-containing protein |
|  |  |  | CPIJ013463 | conserved hypothetical protein |
|  |  |  | CPIJ013464 | conserved hypothetical protein |
|  |  |  | CPIJ013472 | conserved hypothetical protein |
|  |  |  | CPIJ013592 | conserved hypothetical protein |
|  |  |  | CPIJ013676 | cellular retinaldehyde binding protein |
|  |  |  | CPIJ014217 | CRAL/TRIO domain-containing protein |
|  |  |  | CPIJ014225 | CRAL/TRIO domain-containing protein |
|  |  |  | CPIJ016765 | ganglioside induced differentiation associated protein |
|  |  |  | CPIJ018183 | conserved hypothetical protein |
|  |  |  | CPIJ018213 | CRAL/TRIO domain-containing protein |
|  |  | CRAL/TRIO N-terminal domain | CPIJ013466 | conserved hypothetical protein |
|  |  |  | CPIJ014222 | CRAL/TRIO domain-containing protein |
|  |  |  | CPIJ018181 | conserved hypothetical protein |
|  |  |  | CPIJ018184 | conserved hypothetical protein |
|  |  | Phospholipase A2, PLA2 | CPIJ001437 | phospholipase A2 |
|  |  |  | CPIJ011154 | secretory Phospholipase A2 |
|  |  |  | CPIJ011155 | secretory Phospholipase A2 |
|  |  |  | CPIJ019557 | conserved hypothetical protein |
|  |  | PLC-like phosphodiesterases | CPIJ008722 | conserved hypothetical protein |
|  | Proteases | BPTI-like | CPIJ004215 | conserved hypothetical protein |
|  |  | ClpP/crotonase | CPIJ002685 | enoyl-CoA hydratase ECHA12 |
|  |  |  | CPIJ005435 | peroxisomal 3,2-trans-enoyl-CoA isomerase |
|  |  |  | CPIJ009999 | methylcrotonoyl-CoA carboxylase beta chain, mitochondrial |
|  |  |  | CPIJ013793 | cuticle protein 8 |
|  |  |  | CPIJ020263 | fatty acid oxidation complex subunit alpha |
|  |  | Creatinase/aminopeptidase | CPIJ011945 | methionine aminopeptidase 2 |
|  |  |  | CPIJ020069 | methionine aminopeptidase 2 |
|  |  |  | CPIJ006323 | xaa-Pro aminopeptidase 1 |
|  |  |  | CPIJ014907 | xaa-pro dipeptidase |
|  |  | Cystatin/monellin | CPIJ002770 | cystatin-like protein |
|  |  |  | CPIJ002771 | cystatin-like protein |
|  |  | Cysteine proteinases | CPIJ000133 | conserved hypothetical protein |
|  |  |  | CPIJ000575 | oryzain gamma chain |
|  |  |  | CPIJ003218 | ubiquitin carboxyl-terminal hydrolase 22 |
|  |  |  | CPIJ004347 | Autophagy-specific protein |
|  |  |  | CPIJ005164 | ubiquitin specific proteinase |
|  |  |  | CPIJ005165 | ubiquitin specific proteinase |
|  |  |  | CPIJ005440 | conserved hypothetical protein |
|  |  |  | CPIJ007009 | conserved hypothetical protein |
|  |  |  | CPIJ010867 | conserved hypothetical protein |
|  |  |  | CPIJ013438 | conserved hypothetical protein |
|  |  |  | CPIJ014293 | ubiquitin specific protease 2 |
|  |  |  | CPIJ014687 | ubiquitin carboxyl-terminal hydrolase 14 |
|  |  |  | CPIJ014690 | conserved hypothetical protein |
|  |  |  | CPIJ015776 | OTU domain-containing protein 6B |
|  |  |  | CPIJ016134 | ubiquitin carboxyl-terminal hydrolase 64E |
|  |  | DPP6 N-terminal domain-like | CPIJ008362 | DET1 protein |
|  |  | Elafin-like | CPIJ006214 | salivary cysteine-rich peptide |
|  |  |  | CPIJ019874 | salivary cysteine-rich peptide |
|  |  | HSP40/DnaJ peptide-binding domain | CPIJ006891 | tumorous imaginal discs, mitochondrial |
|  |  | Kazal-type serine protease inhibitors | CPIJ011189 | predicted protein |
|  |  | LuxS/MPP-like metallohydrolase | CPIJ001880 | mitochondrial-processing peptidase alpha subunit |
|  |  |  | CPIJ019576 | mitochondrial-processing peptidase subunit beta |
|  |  | Metallo-dependent phosphatases | CPIJ000169 | sphingomyelin phosphodiesterase |
|  |  |  | CPIJ001371 | serine/threonine-protein phosphatase 4 catalytic subunit |
|  |  |  | CPIJ004547 | lariat debranching enzyme |
|  |  |  | CPIJ009664 | purple acid phosphatase |
|  |  | Metalloproteases ("zincins"), catalytic domain | CPIJ001052 | aminopeptidase 2, mitochondrial |
|  |  |  | CPIJ001462 | conserved hypothetical protein |
|  |  |  | CPIJ001808 | conserved hypothetical protein |
|  |  |  | CPIJ005931 | ADAM 17 |
|  |  |  | CPIJ006295 | protease m1 zinc metalloprotease |
|  |  |  | CPIJ011458 | aminopeptidase N |
|  |  |  | CPIJ012680 | ADAM 12 |
|  |  |  | CPIJ013386 | zinc metalloproteinase nas-14 |
|  |  |  | CPIJ014660 | protease m1 zinc metalloprotease |
|  |  |  | CPIJ017830 | conserved hypothetical protein |
|  |  | PMP inhibitors | CPIJ010990 | pacifastin light chain |
|  |  | Protease propeptides/inhibitors | CPIJ016547 | proprotein convertase subtilisin/kexin type 4, furin |
|  |  | Rhomboid-like | CPIJ003969 | stem cell tumor |
|  |  |  | CPIJ014344 | conserved hypothetical protein |
|  |  |  | CPIJ014350 | transmembrane protein 115 |
|  |  |  | CPIJ015372 | rhomboid protein 1, mitochondrial |
|  |  | Serpins | CPIJ000915 | serpin B3 |
|  |  |  | CPIJ005227 | serine protease inhibitor |
|  |  |  | CPIJ010186 | conserved hypothetical protein |
|  |  |  | CPIJ012016 | serine protease inhibitor, serpin |
|  |  |  | CPIJ016299 | serine protease inhibitor, serpin |
|  |  |  | CPIJ017784 | serpin B8 |
|  |  | Subtilisin-like | CPIJ013852 | tripeptidyl-peptidase 2 |
|  |  |  | CPIJ015180 | proprotein convertase subtilisin/kexin type 4, furin |
|  |  |  | CPIJ015181 | proprotein convertase subtilisin/kexin type 4, furin |
|  |  | Thyroglobulin type-1 domain | CPIJ001687 | conserved hypothetical protein |
|  |  | Trypsin-like serine proteases | CPIJ000593 | coagulation factor XI |
|  |  |  | CPIJ001059 | serine protease |
|  |  |  | CPIJ001060 | coagulation factor XI |
|  |  |  | CPIJ001099 | serine protease |
|  |  |  | CPIJ001107 | serine protease 27 |
|  |  |  | CPIJ001109 | serine protease |
|  |  |  | CPIJ001983 | trypsin 5G1 |
|  |  |  | CPIJ002490 | conserved hypothetical protein |
|  |  |  | CPIJ002491 | conserved hypothetical protein |
|  |  |  | CPIJ002531 | serine protease |
|  |  |  | CPIJ004037 | serine protease |
|  |  |  | CPIJ004038 | clip-domain serine protease |
|  |  |  | CPIJ004091 | trypsin eta |
|  |  |  | CPIJ004093 | coagulation factor XI |
|  |  |  | CPIJ004094 | serine protease |
|  |  |  | CPIJ004095 | serine protease |
|  |  |  | CPIJ004304 | serine protease |
|  |  |  | CPIJ004990 | serine protease1/2 |
|  |  |  | CPIJ005480 | serine protease |
|  |  |  | CPIJ005904 | anionic trypsin-2 |
|  |  |  | CPIJ006226 | serine protease |
|  |  |  | CPIJ006544 | chymotrypsinogen 2 |
|  |  |  | CPIJ006568 | chymotrypsin 1 |
|  |  |  | CPIJ006869 | mast cell protease 3 |
|  |  |  | CPIJ008062 | conserved hypothetical protein |
|  |  |  | CPIJ008523 | serine-type enodpeptidase |
|  |  |  | CPIJ008567 | serine protease |
|  |  |  | CPIJ008568 | serine protease |
|  |  |  | CPIJ009113 | chymotrypsin BI |
|  |  |  | CPIJ009142 | coagulation factor VII |
|  |  |  | CPIJ009480 | tryptase gamma |
|  |  |  | CPIJ009592 | serine-type enodpeptidase |
|  |  |  | CPIJ009624 | serine protease |
|  |  |  | CPIJ009792 | conserved hypothetical protein |
|  |  |  | CPIJ009890 | serine proteinase stubble |
|  |  |  | CPIJ009891 | serine protease |
|  |  |  | CPIJ009893 | serine protease |
|  |  |  | CPIJ009894 | serine protease |
|  |  |  | CPIJ010297 | coagulation factor X |
|  |  |  | CPIJ010615 | proclotting enzyme |
|  |  |  | CPIJ011477 | conserved hypothetical protein |
|  |  |  | CPIJ012017 | serine protease |
|  |  |  | CPIJ013043 | chymotrypsin A |
|  |  |  | CPIJ013044 | anionic trypsin |
|  |  |  | CPIJ013362 | conserved hypothetical protein |
|  |  |  | CPIJ013396 | urokinase-type plasminogen activator |
|  |  |  | CPIJ013616 | trypsin 5 |
|  |  |  | CPIJ015405 | serine protease |
|  |  |  | CPIJ016103 | serine protease |
|  |  |  | CPIJ016220 | serine protease |
|  |  |  | CPIJ017794 | 220 kDa silk protein |
|  |  |  | CPIJ017797 | neurohypophysial hormones |
|  |  |  | CPIJ017798 | serine protease |
|  |  |  | CPIJ017990 | serine protease1/2 |
|  |  |  | CPIJ018529 | trypsin 1 |
|  |  |  | CPIJ019031 | chymotrypsin BII |
|  |  |  | CPIJ019291 | serine protease htra2 |
|  |  |  | CPIJ019781 | trypsin 1 |
|  |  |  | CPIJ019952 | serine protease |
|  |  |  | CPIJ020116 | conserved hypothetical protein |
|  |  | Zn-dependent exopeptidases | CPIJ001174 | plasma glutamate carboxypeptidase |
|  |  |  | CPIJ009394 | zinc carboxypeptidase |
|  |  |  | CPIJ009466 | conserved hypothetical protein |
|  |  |  | CPIJ010806 | conserved hypothetical protein |
|  |  |  | CPIJ012908 | glutaminyl-peptide cyclotransferase |
|  |  |  | CPIJ015253 | zinc carboxypeptidase A 1 |
|  |  |  | CPIJ019695 | plasma glutamate carboxypeptidase |
|  |  |  | CPIJ019890 | carboxypeptidase D |
|  | Protein modification | ATPase domain of HSP90 chaperone/DNA topoisomerase II/histidine kinase | CPIJ011247 | heat shock protein 82 |
|  |  | Chaperone J-domain | CPIJ007923 | conserved hypothetical protein |
|  |  |  | CPIJ008583 | DnaJ domain containing protein |
|  |  |  | CPIJ011412 | M-phase phosphoprotein 11 |
|  |  |  | CPIJ017724 | conserved hypothetical protein |
|  |  |  | CPIJ018848 | mitochondrial protein import protein MAS5 |
|  |  | Cyclophilin-like | CPIJ004991 | peptidyl-prolyl cis-trans isomerase |
|  |  |  | CPIJ011947 | peptidyl-prolyl cis-trans isomerase 10 |
|  |  |  | CPIJ016592 | peptidyl-prolyl cis-trans isomerase |
|  |  |  | CPIJ020068 | peptidyl-prolyl cis-trans isomerase cyp8 |
|  |  | FKBP-like | CPIJ011777 | FK506-binding protein 2 |
|  |  |  | CPIJ014691 | FK506-binding protein 59 |
|  |  |  | CPIJ014950 | FK506-binding protein |
|  |  |  | CPIJ014951 | conserved hypothetical protein |
|  |  | GroEL equatorial domain-like | CPIJ008889 | 60 kDa heat shock protein, mitochondrial |
|  |  |  | CPIJ018780 | ribosomal protein S6 |
|  |  | GroES-like | CPIJ000840 | conserved hypothetical protein |
|  |  |  | CPIJ007228 | heat shock protein |
|  |  |  | CPIJ017296 | conserved hypothetical protein |
|  |  | Hect, E3 ligase catalytic domain | CPIJ004914 | ubiquitin-protein ligase |
|  |  |  | CPIJ011644 | ubiquitin-protein ligase |
|  |  |  | CPIJ012813 | conserved hypothetical protein |
|  |  |  | CPIJ017821 | hect type E3 ubiquitin ligase |
|  |  | HSP20-like chaperones | CPIJ005642 | heat shock protein 27 |
|  |  |  | CPIJ005645 | heat shock protein 22 |
|  |  |  | CPIJ007348 | nuclear movement protein nudC |
|  |  |  | CPIJ013743 | integrin beta-1-binding protein 2 |
|  |  |  | CPIJ019282 | NudC domain containing 1 |
|  |  |  | CPIJ019713 | chaperone binding protein |
|  |  | Peptide methionine sulfoxide reductase | CPIJ005204 | peptide methionine sulfoxide reductase msrA |
|  |  |  | CPIJ018565 | peptide methionine sulfoxide reductase |
|  |  | Prefoldin | CPIJ004062 | conserved hypothetical protein |
|  |  |  | CPIJ008888 | conserved hypothetical protein |
|  |  | Tubulin chaperone cofactor A | CPIJ003559 | tubulin-specific chaperone A |
|  |  | UBC-like | CPIJ004186 | ubiquitin-conjugating enzyme morgue |
|  |  |  | CPIJ005149 | ubiquitin-conjugating enzyme E2 i |
|  |  |  | CPIJ005316 | RWD domain-containing protein 4A |
|  |  |  | CPIJ006567 | ubiquitin-conjugating enzyme E2-17 kDa |
|  |  |  | CPIJ007408 | ubiquitin-conjugating enzyme m |
|  |  |  | CPIJ007726 | ubiquitin-conjugating enzyme E2 Q2 |
|  |  |  | CPIJ009955 | ubiquitin conjugating enzyme E2 |
|  |  |  | CPIJ011320 | Ufm1-conjugating enzyme 1 |
|  |  |  | CPIJ013524 | ubiquitin-conjugating enzyme E2 g |
|  |  |  | CPIJ014766 | nedd8-conjugating enzyme nce2 |
|  | Transport | ABC transporter transmembrane region | CPIJ011959 | conserved hypothetical protein |
|  |  |  | CPIJ011961 | multidrug resistance protein 2 |
|  |  | Aquaporin-like | CPIJ016447 | aquaporin transporter |
|  |  | Cap-Gly domain | CPIJ003549 | 150 kDa dynein-associated polypeptide |
|  |  | CBS-domain | CPIJ006251 | AMPK-gamma subunit |
|  |  | ENTH/VHS domain | CPIJ000451 | conserved hypothetical protein |
|  |  |  | CPIJ001186 | conserved hypothetical protein |
|  |  |  | CPIJ001538 | hepatocyte growth factor-regulated tyrosine kinase substrate |
|  |  |  | CPIJ002094 | liquid facets |
|  |  |  | CPIJ010606 | conserved hypothetical protein |
|  |  |  | CPIJ012987 | conserved hypothetical protein |
|  |  |  | CPIJ014748 | conserved hypothetical protein |
|  |  |  | CPIJ019897 | phosphatidylinositol-binding clathrin assembly protein |
|  |  | Glycolipid transfer protein, GLTP | CPIJ010828 | conserved hypothetical protein |
|  |  | LDL receptor-like module | CPIJ004357 | conserved hypothetical protein |
|  |  |  | CPIJ014258 | conserved hypothetical protein |
|  |  |  | CPIJ016346 | conserved hypothetical protein |
|  |  | Lipocalins | CPIJ015727 | apolipoprotein D |
|  |  |  | CPIJ015728 | conserved hypothetical protein |
|  |  |  | CPIJ017615 | apolipoprotein D |
|  |  | Mitochondrial carrier | CPIJ002253 | mitochondrial carnitine/acylcarnitine carrier protein |
|  |  |  | CPIJ006475 | mitochondrial 2-oxoglutarate/malate carrier protein |
|  |  |  | CPIJ001256 | mitochondrial uncoupling protein |
|  |  |  | CPIJ001257 | mitochondrial uncoupling protein |
|  |  |  | CPIJ005941 | ADP,ATP carrier protein 2 |
|  |  |  | CPIJ007010 | peroxisomal membrane protein pmp34 |
|  |  |  | CPIJ007454 | small calcium-binding mitochondrial carrier |
|  |  |  | CPIJ008580 | mitochondrial carrier protein |
|  |  |  | CPIJ011555 | mitochondrial carrier protein |
|  |  |  | CPIJ012095 | mitochondrial carrier protein |
|  |  |  | CPIJ012803 | folate carrier protein |
|  |  |  | CPIJ013684 | Mitochondrial glutamate carrier |
|  |  |  | CPIJ013697 | tricarboxylate transport protein, mitochondrial |
|  |  |  | CPIJ016473 | mitochondrial solute carrier protein |
|  |  |  | CPIJ016474 | mitochondrial solute carrier protein |
|  |  |  | CPIJ019834 | mitochondrial carnitine/acylcarnitine carrier protein |
|  |  |  | CPIJ020183 | folate carrier protein |
|  |  | Multidrug efflux transporter AcrB transmembrane domain | CPIJ002969 | conserved hypothetical protein |
|  |  |  | CPIJ011712 | conserved hypothetical protein |
|  |  |  | CPIJ019195 | conserved hypothetical protein |
|  |  | NTF2-like | CPIJ001995 | conserved hypothetical protein |
|  |  |  | CPIJ005154 | nuclear transport factor 2 |
|  |  | Nucleoporin domain | CPIJ009141 | nuclear pore complex protein nup214 |
|  |  | Phoshotransferase/anion transport protein | CPIJ005835 | sodium bicarbonate cotransporter |
|  |  | Preprotein translocase SecY subunit | CPIJ002966 | transport protein Sec61 subunit alpha 2 |
|  |  | Sec1/munc18-like (SM) proteins | CPIJ007221 | vacuolar protein sorting-associated |
|  |  | Second domain of Mu2 adaptin subunit (ap50) of ap2 adaptor | CPIJ003697 | clathrin coat assembly protein AP50 |
|  |  |  | CPIJ009776 | AP-2 complex subunit mu |
|  |  | SNARE fusion complex | CPIJ007395 | conserved hypothetical protein |
|  |  |  | CPIJ009959 | conserved hypothetical protein |
|  |  |  | CPIJ010472 | conserved hypothetical protein |
|  |  |  | CPIJ011681 | synaptosomal-associated protein 29 |
| Metabolism | Amino acids m/tr | SRP19 | CPIJ012130 | conserved hypothetical protein |
|  |  | Alanine racemase C-terminal domain-like | CPIJ010687 | ornithine decarboxylase |
|  |  | Arginase/deacetylase | CPIJ011847 | histone deacetylase |
|  |  |  | CPIJ019172 | histone deacetylase |
|  |  | Glutaminase/Asparaginase | CPIJ008684 | l-asparaginase i |
|  |  | L-aspartase-like | CPIJ002702 | adenylosuccinate lyase |
|  |  | PLP-binding barrel | CPIJ008556 | ornithine decarboxylase |
|  |  |  | CPIJ010688 | ornithine decarboxylase |
|  | Carbohydrate m/tr | Tryptophan synthase beta subunit-like PLP-dependent enzymes | CPIJ011197 | threonine dehydratase/deaminase |
|  |  | (Trans)glycosidases | CPIJ002066 | alpha-galactosidase A |
|  |  |  | CPIJ003944 | brain chitinase and chia |
|  |  |  | CPIJ004564 | brain chitinase and chia |
|  |  |  | CPIJ008532 | glycoside hydrolase |
|  |  |  | CPIJ011854 | CD98hc amino acid transporter protein |
|  |  |  | CPIJ012134 | brain chitinase and chia |
|  |  |  | CPIJ013476 | chitooligosaccharidolytic beta-N-acetylglucosaminidase |
|  |  |  | CPIJ014063 | glycoside hydrolase |
|  |  |  | CPIJ015627 | alpha-N-acetyl glucosaminidase |
|  |  |  | CPIJ018222 | alpha-amylase B |
|  |  | Aldolase | CPIJ006003 | delta-aminolevulinic acid dehydratase |
|  |  | Carbohydrate phosphatase | CPIJ016359 | myo inositol monophosphatase |
|  |  | Galactose-binding domain-like | CPIJ003832 | thioredoxin family Trp26 |
|  |  |  | CPIJ008825 | conserved hypothetical protein |
|  |  |  | CPIJ011079 | discoidin domain receptor |
|  |  |  | CPIJ014812 | eph receptor tyrosine kinase |
|  |  | HIT-like | CPIJ005586 | histidine triad protein member |
|  |  | Invertebrate chitin-binding proteins | CPIJ016342 | conserved hypothetical protein |
|  |  |  | CPIJ000248 | conserved hypothetical protein |
|  |  |  | CPIJ000681 | obstractor B |
|  |  |  | CPIJ003955 | predicted protein |
|  |  |  | CPIJ004334 | conserved hypothetical protein |
|  |  |  | CPIJ004728 | conserved hypothetical protein |
|  |  |  | CPIJ006133 | conserved hypothetical protein |
|  |  |  | CPIJ007317 | chitin binding protein |
|  |  |  | CPIJ007603 | conserved hypothetical protein |
|  |  |  | CPIJ007661 | conserved hypothetical protein |
|  |  |  | CPIJ007662 | conserved hypothetical protein |
|  |  |  | CPIJ008466 | conserved hypothetical protein |
|  |  |  | CPIJ008502 | conserved hypothetical protein |
|  |  |  | CPIJ008558 | conserved hypothetical protein |
|  |  |  | CPIJ009078 | conserved hypothetical protein |
|  |  |  | CPIJ009407 | conserved hypothetical protein |
|  |  |  | CPIJ009969 | conserved hypothetical protein |
|  |  |  | CPIJ011482 | conserved hypothetical protein |
|  |  |  | CPIJ012138 | conserved hypothetical protein |
|  |  |  | CPIJ012316 | conserved hypothetical protein |
|  |  |  | CPIJ012665 | conserved hypothetical protein |
|  |  |  | CPIJ013980 | conserved hypothetical protein |
|  |  |  | CPIJ014180 | conserved hypothetical protein |
|  |  |  | CPIJ014194 | conserved hypothetical protein |
|  |  |  | CPIJ014195 | conserved hypothetical protein |
|  |  |  | CPIJ014197 | conserved hypothetical protein |
|  |  |  | CPIJ014267 | conserved hypothetical protein |
|  |  |  | CPIJ015173 | conserved hypothetical protein |
|  |  |  | CPIJ015174 | conserved hypothetical protein |
|  |  |  | CPIJ015734 | conserved hypothetical protein |
|  |  |  | CPIJ016344 | conserved hypothetical protein |
|  |  |  | CPIJ018321 | conserved hypothetical protein |
|  |  |  | CPIJ018323 | conserved hypothetical protein |
|  |  |  | CPIJ018465 | conserved hypothetical protein |
|  |  |  | CPIJ020138 | conserved hypothetical protein |
|  |  | Seven-hairpin glycosidases | CPIJ006601 | conserved hypothetical protein |
|  |  |  | CPIJ009935 | mannosyl-oligosaccharide alpha-1,2-mannosidase |
|  | Coenzyme m/tr | Six-hairpin glycosidases | CPIJ008855 | maltose phosphorylase |
|  |  | Activating enzymes of the ubiquitin-like proteins | CPIJ013962 | sumo-1-activating enzyme E1a |
|  |  |  | CPIJ016556 | ubiquitin-activating enzyme E1 |
|  |  | Acyl-CoA dehydrogenase NM domain-like | CPIJ008217 | acyl-coa dehydrogenase |
|  |  |  | CPIJ014783 | isovaleryl-CoA dehydrogenase, mitochondrial |
|  |  |  | CPIJ016451 | crotonobetainyl-CoA dehydrogenase |
|  |  |  | CPIJ016453 | acyl-coa dehydrogenase |
|  |  |  | CPIJ016454 | acyl-coa dehydrogenase |
|  |  | Class II aaRS and biotin synthetases | CPIJ019538 | aspartyl-tRNA synthetase |
|  |  |  | CPIJ001182 | asparaginyl-tRNA synthetase |
|  |  |  | CPIJ013145 | prolyl-tRNA synthetase |
|  |  |  | CPIJ016067 | phenylalanyl-tRNA synthetase beta chain |
|  |  | Glutathione synthetase ATP-binding domain-like | CPIJ003283 | conserved hypothetical protein |
|  |  |  | CPIJ009145 | phosphoribosylamine-glycine ligase |
|  |  |  | CPIJ013436 | conserved hypothetical protein |
|  |  | PCD-like | CPIJ006712 | conserved hypothetical protein |
|  |  | Peptide deformylase | CPIJ011016 | peptide deformylase, mitochondrial |
|  |  | S-adenosylmethionine decarboxylase | CPIJ005587 | s-adenosyl methionine decarboxylase |
|  |  | Substrate-binding domain of HMG-CoA reductase | CPIJ004077 | 3-hydroxy-3-methylglutaryl-coenzyme A reductase |
|  |  | UROD/MetE-like | CPIJ010693 | uroporphyrinogen decarboxylase |
|  |  | Cytochrome b5-like heme/steroid binding domain | CPIJ000318 | cytochrome b5 |
|  |  |  | CPIJ010629 | membrane associated progesterone receptor |
|  |  |  | CPIJ013832 | cytochrome b5 |
|  |  |  | CPIJ018120 | flavohemoprotein B5/b5r |
|  |  | Cytochrome c oxidase subunit h | CPIJ012223 | cytochrome c oxidase,-subunit VIb |
|  |  | FMN-dependent nitroreductase-like | CPIJ012177 | iodotyrosine dehalogenase 1 |
|  |  | Molybdenum cofactor-binding domain | CPIJ013919 | xanthine dehydrogenase/oxidase |
|  |  |  | CPIJ013920 | aldehyde oxidase |
|  |  |  | CPIJ013921 | aldehyde oxidase |
|  |  |  | CPIJ013934 | xanthine dehydrogenase/oxidase |
|  | Energy | 6-phosphogluconate dehydrogenase C-terminal domain-like | CPIJ008427 | conserved hypothetical protein |
|  |  |  | CPIJ012165 | conserved hypothetical protein |
|  |  |  | CPIJ013021 | 6-phosphogluconate dehydrogenase |
|  |  | Citrate synthase | CPIJ019860 | citrate synthase |
|  |  | Enolase C-terminal domain-like | CPIJ013600 | mandelate racemase |
|  |  | Mitochondrial cytochrome c oxidase subunit VIIa | CPIJ014384 | conserved hypothetical protein |
|  |  | PEP carboxykinase-like | CPIJ010515 | phosphoenolpyruvate carboxykinase |
|  |  | Vacuolar ATP synthase subunit C |  |  |
|  | Lipid m/tr | Acyl-CoA binding protein | CPIJ019707 | conserved hypothetical protein |
|  |  | Creatinase/prolidase N-terminal domain | CPIJ016993 | xaa-pro dipeptidase |
|  |  | Lipovitellin-phosvitin complex, superhelical domain | CPIJ002028 | conserved hypothetical protein |
|  |  | Thioesterase/thiol ester dehydrase-isomerase | CPIJ009653 | conserved hypothetical protein |
|  |  | YWTD domain | CPIJ000808 | low-density lipoprotein receptor |
|  |  |  | CPIJ017507 | low-density lipoprotein receptor |
|  | Nitrogen m/tr | RmlC-like cupins | CPIJ003551 | conserved hypothetical protein |
|  | Nucleotide m/tr | dUTPase-like | CPIJ005616 | deoxyuridine 5'-triphosphate nucleotidohydrolase |
|  |  | Nucleoside hydrolase | CPIJ008181 | inosine-uridine preferring nucleoside hydrolase |
|  |  |  | CPIJ014047 | inosine-uridine preferring nucleoside hydrolase |
|  |  | Nucleotidyltransferase | CPIJ010886 | conserved hypothetical protein |
|  |  | Nucleotidylyl transferase | CPIJ010393 | cysteinyl-tRNA synthetase |
|  |  |  | CPIJ010526 | cysteinyl-tRNA synthetase |
|  |  | PRTase-like | CPIJ004528 | conserved hypothetical protein |
|  |  |  | CPIJ004967 | uracil phosphoribosyltransferase |
|  |  |  | CPIJ012747 | uridine cytidine kinase i |
|  |  | Pseudouridine synthase | CPIJ002499 | ribosomal pseudouridine synthase |
|  |  |  | CPIJ014146 | conserved hypothetical protein |
|  |  | Ribonuclease H-like | CPIJ002928 | conserved hypothetical protein |
|  |  |  | CPIJ005339 | ATP-binding cassette transporter |
|  |  |  | CPIJ008015 | conserved hypothetical protein |
|  |  |  | CPIJ010267 | 3'-5' exonuclease |
|  |  | Ribulose-phoshate binding barrel | CPIJ008100 | conserved hypothetical protein |
|  |  | SAICAR synthase-like | CPIJ001991 | inositol triphosphate 3-kinase c |
|  |  |  | CPIJ001992 | inositol triphosphate 3-kinase c |
|  |  | Tetrahydrobiopterin biosynthesis enzymes-like | CPIJ000863 | 6-pyruvoyl tetrahydrobiopterin synthase |
|  |  |  | CPIJ014857 | GTP cyclohydrolase i |
|  |  |  | CPIJ018483 | GTP cyclohydrolase i |
|  |  | Acetyl-CoA synthetase-like | CPIJ000424 | AMP dependent coa ligase |
|  |  |  | CPIJ000425 | short-chain-fatty-acid-CoA ligase |
|  |  |  | CPIJ002867 | AMP dependent coa ligase |
|  |  |  | CPIJ007302 | long-chain fatty acid transport protein 4 |
|  |  |  | CPIJ009978 | AMP dependent coa ligase |
|  |  |  | CPIJ009981 | conserved hypothetical protein |
|  |  |  | CPIJ011600 | long-chain-fatty-acid coa ligase |
|  |  |  | CPIJ015670 | 4-coumarate-CoA ligase 3 |
|  |  |  | CPIJ015716 | 4-coumarate-CoA ligase 1 |
|  |  |  | CPIJ017396 | AMP dependent ligase |
|  |  |  | CPIJ018155 | luciferin 4-monooxygenase |
|  |  | Actin-like ATPase domain | CPIJ004484 | conserved hypothetical protein |
|  |  |  | CPIJ006534 | conserved hypothetical protein |
|  |  |  | CPIJ011081 | heat shock protein 70 B2 |
|  |  |  | CPIJ011082 | heat shock protein 70 B2 |
|  |  |  | CPIJ011083 | heat shock protein 70 B2 |
|  |  |  | CPIJ019868 | heat shock 70 kDa protein 4 |
|  |  | Alkaline phosphatase-like | CPIJ001263 | membrane-bound alkaline phosphatase |
|  |  |  | CPIJ002095 | alkaline phosphatase |
|  |  |  | CPIJ006774 | arylsulfatase B |
|  |  |  | CPIJ010201 | heparan n-sulfatase |
|  |  |  | CPIJ010661 | conserved hypothetical protein |
|  |  |  | CPIJ011047 | arylsulfatase b |
|  |  |  | CPIJ015241 | alkaline phosphatase |
|  |  |  | CPIJ017042 | conserved hypothetical protein |
|  |  | alpha/beta-Hydrolases | CPIJ000367 | lysosomal acid lipase |
|  |  |  | CPIJ001035 | conserved hypothetical protein |
|  |  |  | CPIJ001352 | N-myc downstream regulated |
|  |  |  | CPIJ002719 | lipase 1 |
|  |  |  | CPIJ002720 | lysosomal acid lipase |
|  |  |  | CPIJ002721 | lysosomal acid lipase |
|  |  |  | CPIJ002722 | lipase 1 |
|  |  |  | CPIJ002723 | lysosomal acid lipase |
|  |  |  | CPIJ002726 | lipase 3 |
|  |  |  | CPIJ004066 | juvenile hormone esterase |
|  |  |  | CPIJ004226 | pancreatic triacylglycerol lipase |
|  |  |  | CPIJ004636 | para-nitrobenzyl esterase |
|  |  |  | CPIJ004802 | endothelial lipase |
|  |  |  | CPIJ006220 | conserved hypothetical protein |
|  |  |  | CPIJ007141 | esterase FE4 |
|  |  |  | CPIJ007424 | juvenile hormone esterase |
|  |  |  | CPIJ007825 | para-nitrobenzyl esterase |
|  |  |  | CPIJ010991 | neural stem cell-derived dendrite regulator |
|  |  |  | CPIJ013280 | lysosomal Pro-X carboxypeptidase |
|  |  |  | CPIJ013720 | conserved hypothetical protein |
|  |  |  | CPIJ013838 | lipase 1 |
|  |  |  | CPIJ013918 | esterase B1 |
|  |  |  | CPIJ014154 | esterase FE4 |
|  |  |  | CPIJ015386 | hepatic triacylglycerol lipase |
|  |  |  | CPIJ015557 | Sn1-specific diacylglycerol lipase alpha |
|  |  |  | CPIJ018753 | juvenile hormone esterase |
|  |  |  | CPIJ019227 | pancreatic triacylglycerol lipase |
|  |  |  | CPIJ019228 | pancreatic triacylglycerol lipase |
|  |  |  | CPIJ019996 | conserved hypothetical protein |
|  |  | Amidase signature (AS) enzymes | CPIJ005591 | indoleacetamide hydrolase |
|  |  | Calcium-dependent phosphotriesterase | CPIJ003362 | odd Oz protein |
|  |  | Carbonic anhydrase | CPIJ001807 | carbonic anhydrase |
|  |  |  | CPIJ011424 | carbonic anhydrase |
|  |  |  | CPIJ011533 | carbonic anhydrase |
|  |  |  | CPIJ014280 | carbonic anhydrase |
|  |  | Casein kinase II beta subunit | CPIJ014996 | casein kinase II subunit beta |
|  |  | DHH phosphoesterases | CPIJ005338 | PRUNE protein |
|  |  | DHS-like NAD/FAD-binding domain | CPIJ002993 | deoxyhypusine synthase |
|  |  | F1 ATPase inhibitor, IF1, C-terminal domain | CPIJ000503 | mitochondrial ATPase inhibitor |
|  |  | Folate-binding domain | CPIJ014981 | aminomethyltransferase, mitochondrial |
|  |  | Galactose mutarotase-like | CPIJ015655 | lysosomal alpha-mannosidase |
|  |  |  | CPIJ015656 | lysosomal alpha-mannosidase |
|  |  | Glycoside hydrolase/deacetylase | CPIJ006311 | conserved hypothetical protein |
|  |  |  | CPIJ008266 | conserved hypothetical protein |
|  |  |  | CPIJ008267 | conserved hypothetical protein |
|  |  |  | CPIJ018088 | conserved hypothetical protein |
|  |  | HAD-like | CPIJ010121 | copper-transporting ATPase 1 |
|  |  |  | CPIJ008694 | conserved hypothetical protein |
|  |  |  | CPIJ010605 | conserved hypothetical protein |
|  |  |  | CPIJ010899 | conserved hypothetical protein |
|  |  |  | CPIJ013914 | dullard protein |
|  |  | HD-domain/PDEase-like | CPIJ000309 | sam/hd domain protein |
|  |  | Kinase associated domain 1, KA1 | CPIJ006188 | conserved hypothetical protein |
|  |  |  | CPIJ015835 | conserved hypothetical protein |
|  |  | LysM domain | CPIJ013415 | nucleolar protein c7b |
|  |  | Metallo-dependent hydrolases | CPIJ002583 | Ampd2 protein |
|  |  |  | CPIJ009741 | N-acetylglucosamine-6-phosphate deacetylase |
|  |  | N-acetylmuramoyl-L-alanine amidase-like | CPIJ006558 | peptidoglycan recognition protein la |
|  |  |  | CPIJ008514 | peptidoglycan recognition protein-1 |
|  |  | N-terminal nucleophile aminohydrolases (Ntn hydrolases) | CPIJ000897 | proteasome subunit alpha type 1 |
|  |  |  | CPIJ001361 | proteasome subunit beta type 3 |
|  |  |  | CPIJ003586 | proteasome subunit alpha type 3 |
|  |  |  | CPIJ006946 | proteasome subunit alpha type 2 |
|  |  |  | CPIJ008264 | proteasome subunit beta type 7 |
|  |  |  | CPIJ009861 | proteasome component PRE2 |
|  |  |  | CPIJ016242 | proteasome subunit beta type 5,8 |
|  |  |  | CPIJ016997 | proteasome subunit beta type 5,8 |
|  |  |  | CPIJ017386 | proteasome subunit beta type 8 |
|  |  |  | CPIJ017722 | gamma glutamyl transpeptidase |
|  |  |  | CPIJ019606 | asparagine synthetase |
|  |  | NAD kinase | CPIJ009966 | sphingosine kinase a, b |
|  |  | NHL repeat | CPIJ003685 | tripartite motif protein trim2,3 |
|  |  |  | CPIJ003686 | conserved hypothetical protein |
|  |  | Peptidyl-tRNA hydrolase domain-like | CPIJ007051 | immature colon carcinoma |
|  |  | PFL-like glycyl radical enzymes | CPIJ005992 | ribonucleoside-diphosphate reductase large subunit |
|  |  | Phosphoglycerate mutase-like | CPIJ016005 | acid phosphatase-1 |
|  |  |  | CPIJ002955 | multiple inositol polyphosphate phosphatase |
|  |  |  | CPIJ009604 | phosphoglycerate mutase family member 5 |
|  |  |  | CPIJ011248 | multiple inositol polyphosphate phosphatase 1 |
|  |  |  | CPIJ016006 | conserved hypothetical protein |
|  |  | Phospholipase D/nuclease | CPIJ006211 | tyrosyl-dna phosphodiesterase |
|  |  |  | CPIJ009798 | conserved hypothetical protein |
|  |  | PurM C-terminal domain-like | CPIJ009144 | phosphoribosylamine-glycine ligase |
|  |  | Quinoprotein alcohol dehydrogenase-like | CPIJ000465 | conserved hypothetical protein |
|  |  |  | CPIJ000963 | kinesin family member 21A |
|  |  |  | CPIJ007852 | receptor for activated protein kinase C |
|  |  |  | CPIJ011019 | wd-repeat protein |
|  |  |  | CPIJ014368 | conserved hypothetical protein |
|  |  |  | CPIJ019807 | proliferation-inducing gene 21 |
|  |  | Ribokinase-like | CPIJ011108 | conserved hypothetical protein |
|  |  |  | CPIJ011111 | conserved hypothetical protein |
|  |  |  | CPIJ016881 | conserved hypothetical protein |
|  |  |  | CPIJ020058 | pyridoxal kinase |
|  |  | SGNH hydrolase | CPIJ011741 | platelet-activating factor acetylhydrolase IB subunit beta |
|  |  |  | CPIJ012575 | phospholipase b |
|  |  |  | CPIJ012576 | phospholipase b |
|  |  |  | CPIJ012577 | phospholipase b, plb1 |
|  |  |  | CPIJ016880 | phospholipase b, plb1 |
|  |  | Thiolase-like | CPIJ002342 | 3-ketoacyl-CoA thiolase |
|  |  |  | CPIJ018065 | trifunctional enzyme beta subunit |
|  |  | Trimeric LpxA-like enzymes | CPIJ003121 | dynactin subunit 5 |
|  | Photosynthesis | PRC-barrel domain | CPIJ005871 | conserved hypothetical protein |
|  | Polysaccharide m/tr | DAK1/DegV-like | CPIJ014451 | conserved hypothetical protein |
|  |  |  | CPIJ016133 | dihydroxyacetone kinase |
|  |  | Ricin B-like lectins | CPIJ005695 | polypeptide N-acetylgalactosaminyltransferase 5 |
|  |  |  | CPIJ014647 | N-acetyl galactosaminyl transferase 7 |
|  |  |  | CPIJ017873 | 16.7 kDa salivary peptide |
|  |  | RuBisCo LSMT C-terminal, substrate-binding domain | CPIJ018263 | conserved hypothetical protein |
|  |  | Starch-binding domain-like | CPIJ011486 | NOMO3 protein |
|  |  | UDP-Glycosyltransferase/glycogen phosphorylase | CPIJ000038 | UDP-glucuronosyltransferase 1-3 |
|  |  |  | CPIJ004369 | glucosyl transferase |
|  |  |  | CPIJ010412 | fucosyltransferase 11 |
|  |  |  | CPIJ013202 | glycoprotein 3-alpha-L-fucosyltransferase A |
|  |  |  | CPIJ014333 | glucosyl/glucuronosyl transferase |
|  | Redox | 2Fe-2S ferredoxin-like | CPIJ020265 | aldehyde oxidase |
|  |  | Acid phosphatase/Vanadium-dependent haloperoxidase | CPIJ003606 | dolichyldiphosphatase 1 |
|  |  | ALDH-like | CPIJ013217 | glutamate semialdehyde dehydrogenase |
|  |  | Aromatic aminoacid monoxygenases, catalytic and oligomerization domains | CPIJ014156 | conserved hypothetical protein |
|  |  | Cu,Zn superoxide dismutase-like | CPIJ000146 | superoxide dismutase 2 |
|  |  | Cytochrome P450^‡^ | CPIJ018854 | *CYP4C50v2* |
|  |  |  | CPIJ001754 | *CYP4J6* |
|  |  |  | CPIJ001757 | *CYP4H39* |
|  |  |  | CPIJ001810 | *CPY4C38* |
|  |  |  | CPIJ003361 | *CPY6BY2* |
|  |  |  | CPIJ003375 | *CYP6BY3* |
|  |  |  | CPIJ005899 | *CYP6N26P* |
|  |  |  | CPIJ006321 | ^*^SCOP predicted cytochrome P450 |
|  |  |  | CPIJ006322 | *CYP307A1* |
|  |  |  | CPIJ008972 | *CYP6F5P* |
|  |  |  | CPIJ010810 | *CYP325BC2* |
|  |  |  | CPIJ016355 | *CYP6AK1-de1b* |
|  |  |  | CPIJ016846 | *CYP6M13* |
|  |  |  | CPIJ016847 | *CYP6CQ2* |
|  |  |  | CPIJ016849 | *CYP6M12* |
|  |  |  | CPIJ016850 | *CYP6Y4* |
|  |  |  | CPIJ016853 | *CYP6N21P* |
|  |  |  | CPIJ016854 | *CYP6N22* |
|  |  |  | CPIJ016856 | *CYP6N18* |
|  |  |  | CPIJ017245 | *CYP304B6* |
|  |  |  | CPIJ017351 | *CYP4C50v1* |
|  |  |  | CPIJ018716 | *CYP4C38* |
|  |  |  | CPIJ019704 | *CYP6N24* |
|  |  | FAD-dependent thiol oxidase | CPIJ012226 | augmenter of liver regeneration |
|  |  | FAD/NAD-linked reductases, dimerisation (C-terminal) domain | CPIJ002642 | apoptosis-inducing factor 1, mitochondrial |
|  |  | Ferredoxin reductase-like, C-terminal NADP-linked domain | CPIJ003578 | conserved hypothetical protein |
|  |  | Formate/glycerate dehydrogenase catalytic domain-like | CPIJ006365 | conserved hypothetical protein |
|  |  |  | CPIJ011531 | adenosyl homocysteinase |
|  |  | Heme-dependent peroxidases | CPIJ003117 | dual oxidase 1 |
|  |  |  | CPIJ016742 | thyroid peroxidase |
|  |  |  | CPIJ018105 | chorion peroxidase |
|  |  | Inosine monophosphate dehydrogenase (IMPDH) | CPIJ011687 | inosine-5'-monophosphate dehydrogenase |
|  |  | Metallo-hydrolase/oxidoreductase | CPIJ011621 | conserved hypothetical protein |
|  |  |  | CPIJ011625 | conserved hypothetical protein |
|  |  |  | CPIJ019501 | hydroxyacylglutathione hydrolase |
|  |  |  | CPIJ019503 | DNA cross-link repair 1A protein |
|  |  | NAD(P)-linked oxidoreductase | CPIJ003374 | aldo-keto reductase |
|  |  |  | CPIJ003393 | aldose reductase |
|  |  |  | CPIJ003722 | aldo-keto reductase |
|  |  |  | CPIJ017461 | aldo-keto reductase |
|  |  | PHM/PNGase F | CPIJ014202 | dopamine beta hydroxylase |
|  |  | Thioredoxin-like | CPIJ001856 | conserved hypothetical protein |
|  |  |  | CPIJ003089 | SCO1, mitochondrial |
|  |  |  | CPIJ003399 | peroxiredoxins, prx-1, prx-2, prx-3 |
|  |  |  | CPIJ003709 | thioredoxin, mitochondrial |
|  |  |  | CPIJ003981 | 15 kDa selenoprotein |
|  |  |  | CPIJ007327 | disulfide-isomerase A6 |
|  |  |  | CPIJ008802 | conserved hypothetical protein |
|  |  |  | CPIJ009940 | conserved hypothetical protein |
|  |  |  | CPIJ010610 | NADH-ubiquinone oxidoreductase B8 subunit |
|  |  |  | CPIJ011296 | peroxiredoxin 6 |
|  |  |  | CPIJ012568 | phospholipid hydroperoxide glutathione peroxidase 1 |
|  |  |  | CPIJ015346 | glutaredoxin, grx |
|  |  |  | CPIJ016175 | glutaredoxin, grx |
|  |  |  | CPIJ017364 | endoplasmic reticulum resident protein |
|  |  |  | CPIJ017625 | conserved hypothetical protein |
|  | Secondary metabolism | Clavaminate synthase-like | CPIJ014046 | conserved hypothetical protein |
|  |  |  | CPIJ014507 | conserved hypothetical protein |
|  |  |  | CPIJ017090 | gamma-butyrobetaine dioxygenase |
|  |  |  | CPIJ017091 | gamma-butyrobetaine dioxygenase |
|  |  |  | CPIJ018084 | uty-prov protein |
|  |  | Concanavalin A-like lectins/glucanases | CPIJ001299 | keratinocyte lectin |
|  |  |  | CPIJ004321 | gram-negative bacteria binding protein |
|  |  |  | CPIJ004683 | laminin alpha-1, 2 chain |
|  |  |  | CPIJ004919 | conserved hypothetical protein |
|  |  |  | CPIJ005988 | conserved hypothetical protein |
|  |  |  | CPIJ006598 | tripartite motif protein trim9 |
|  |  |  | CPIJ012172 | conserved hypothetical protein |
|  |  |  | CPIJ012874 | kinase c-binding protein nell1 |
|  |  |  | CPIJ013642 | conserved hypothetical protein |
|  |  |  | CPIJ016123 | conserved hypothetical protein |
|  |  | Homo-oligomeric flavin-containing Cys decarboxylases, HFCD | CPIJ019818 | phosphopantothenoylcysteine decarboxylase |
|  |  | Terpenoid synthases | CPIJ008089 | conserved hypothetical protein |
|  |  |  | CPIJ016309 | candidate tumor suppressor protein |
|  |  |  | CPIJ016310 | candidate tumor suppressor protein |
|  |  |  | CPIJ016311 | decaprenyl-diphosphate synthase subunit 2 |
|  | Transferases | 4'-phosphopantetheinyl transferase | CPIJ011416 | aminoadipate-semialdehyde dehydrogenase |
|  |  | Acyl-CoA N-acyltransferases (Nat) | CPIJ000413 | conserved hypothetical protein |
|  |  |  | CPIJ001343 | histone acetyltransferase type B catalytic subunit |
|  |  |  | CPIJ008392 | N-acetyltransferase 5 |
|  |  |  | CPIJ010396 | conserved hypothetical protein |
|  |  |  | CPIJ012930 | conserved hypothetical protein |
|  |  |  | CPIJ015282 | dopamine N acetyltransferase |
|  |  |  | CPIJ015982 | N-acetyl transferase separation anxiety |
|  |  | Class I glutamine amidotransferase-like | CPIJ006930 | gamma-glutamyl hydrolase |
|  |  | CoA-dependent acyltransferases | CPIJ001609 | choline O-acetyltransferase |
|  |  |  | CPIJ005612 | carnitine o-acyltransferase |
|  |  | Formyltransferase | CPIJ009143 | phosphoribosylglycinamide formyltransferase |
|  |  | Glycerol-3-phosphate (1)-acyltransferase | CPIJ004138 | 1-acyl-sn-glycerol-3-phosphate acyltransferase |
|  |  |  | CPIJ004141 | 1-acyl-sn-glycerol-3-phosphate acyltransferase beta |
|  |  |  | CPIJ013939 | glycerol-3-phosphate acyltransferase |
|  |  |  | CPIJ015965 | transmembrane protein 68 |
|  |  | Homocysteine S-methyltransferase | CPIJ008869 | homocysteine S-methyltransferase |
|  |  | MIR domain | CPIJ016258 | probable ER retained protein |
|  |  | NagB/RpiA/CoA transferase-like | CPIJ004258 | ribose-5-phosphate isomerase |
|  |  |  | CPIJ005163 | conserved hypothetical protein |
|  |  |  | CPIJ006915 | translation initiation factor 2b, delta subunit |
|  |  |  | CPIJ008074 | glucosamine-6-phosphate isomerase |
|  |  |  | CPIJ011933 | conserved hypothetical protein |
|  |  | Nucleotide-diphospho-sugar transferases | CPIJ003171 | UDP-n-acteylglucosamine pyrophosphorylase |
|  |  |  | CPIJ000257 | conserved hypothetical protein |
|  |  |  | CPIJ002650 | dolichol-phosphate mannosyltransferase |
|  |  |  | CPIJ004318 | galactosylgalactosylxylosylprotein 3-beta-glucuronosyltransferase I |
|  |  |  | CPIJ005229 | N-acetyl galactosaminyl transferase 6 |
|  |  |  | CPIJ012815 | mannose-1-phosphate guanyltransferase |
|  |  |  | CPIJ016255 | chitin synthase |
|  |  |  | CPIJ018702 | beta-1,3-galactosyltransferase brn |
|  |  | PLP-dependent transferases | CPIJ003522 | cysteine desulfurase, mitochondrial |
|  |  |  | CPIJ010034 | glutamate decarboxylase |
|  |  |  | CPIJ013307 | aromatic-L-amino-acid decarboxylase |
|  |  | Protein prenylyltransferase | CPIJ005820 | geranylgeranyl transferase type-2 alpha subunit |
|  |  |  | CPIJ017557 | smile protein |
|  |  | S-adenosyl-L-methionine-dependent methyltransferases | CPIJ001152 | HemK methyltransferase family member 2 |
|  |  |  | CPIJ001336 | ribosomal RNA large subunit methyltransferase J |
|  |  |  | CPIJ001402 | histone-arginine methyltransferase CARM1 |
|  |  |  | CPIJ001578 | conserved hypothetical protein |
|  |  |  | CPIJ005043 | conserved hypothetical protein |
|  |  |  | CPIJ006933 | conserved hypothetical protein |
|  |  |  | CPIJ007234 | AdoMet-dependent rRNA methyltransferase spb1 |
|  |  |  | CPIJ008978 | conserved hypothetical protein |
|  |  |  | CPIJ010001 | tRNA methyltransferase |
|  |  |  | CPIJ010915 | arginine n-methyltransferase |
|  |  |  | CPIJ013558 | 23S rRNA methyltransferase |
|  |  |  | CPIJ016651 | HemK methyltransferase family member 1 |
|  |  |  | CPIJ018143 | conserved hypothetical protein |
| Regulation |  |  | CPIJ009244 | conserved hypothetical protein |
|  | DNA-binding | AlbA-like | CPIJ010569 | conserved hypothetical protein |
|  |  | AN1-like Zinc finger | CPIJ002707 | zinc finger protein |
|  |  |  | CPIJ002783 | AN1-type zinc finger protein 2B |
|  |  | ARID-like | CPIJ008131 | receptor for activated protein kinase C |
|  |  | ATP-dependent DNA ligase DNA-binding domain | CPIJ017286 | conserved hypothetical protein |
|  |  | Bromodomain | CPIJ007940 | conserved hypothetical protein |
|  |  |  | CPIJ012613 | conserved hypothetical protein |
|  |  | C2H2 and C2HC zinc fingers | CPIJ018818 | conserved hypothetical protein |
|  |  |  | CPIJ000409 | conserved hypothetical protein |
|  |  |  | CPIJ000911 | zinc finger protein 383 |
|  |  |  | CPIJ001029 | serendipity locus protein delta |
|  |  |  | CPIJ001300 | zinc finger protein 780B |
|  |  |  | CPIJ001471 | transcription factor hamlet |
|  |  |  | CPIJ001473 | conserved hypothetical protein |
|  |  |  | CPIJ001552 | conserved hypothetical protein |
|  |  |  | CPIJ001985 | conserved hypothetical protein |
|  |  |  | CPIJ002705 | zinc finger protein 90 |
|  |  |  | CPIJ002824 | predicted protein |
|  |  |  | CPIJ002932 | conserved hypothetical protein |
|  |  |  | CPIJ003270 | broad-complex core-protein |
|  |  |  | CPIJ003609 | Sp5 transcription factor |
|  |  |  | CPIJ003667 | zinc finger protein 141 |
|  |  |  | CPIJ003749 | tRNA delta |
|  |  |  | CPIJ003796 | conserved hypothetical protein |
|  |  |  | CPIJ004257 | zinc finger-containing protein |
|  |  |  | CPIJ004351 | zinc finger protein 92 |
|  |  |  | CPIJ004384 | zinc finger protein |
|  |  |  | CPIJ004667 | zinc finger protein |
|  |  |  | CPIJ004785 | conserved hypothetical protein |
|  |  |  | CPIJ004963 | conserved hypothetical protein |
|  |  |  | CPIJ005175 | transcription factor sp8,sp9 |
|  |  |  | CPIJ005503 | zinc finger protein 36 |
|  |  |  | CPIJ005812 | zinc finger protein |
|  |  |  | CPIJ005813 | zinc finger protein |
|  |  |  | CPIJ006385 | conserved hypothetical protein |
|  |  |  | CPIJ006765 | conserved hypothetical protein |
|  |  |  | CPIJ006854 | conserved hypothetical protein |
|  |  |  | CPIJ006855 | zinc finger protein |
|  |  |  | CPIJ007837 | zinc finger protein |
|  |  |  | CPIJ007858 | conserved hypothetical protein |
|  |  |  | CPIJ008060 | conserved hypothetical protein |
|  |  |  | CPIJ008297 | conserved hypothetical protein |
|  |  |  | CPIJ008361 | double-stranded RNA-binding protein zn72d |
|  |  |  | CPIJ008549 | conserved hypothetical protein |
|  |  |  | CPIJ008696 | conserved hypothetical protein |
|  |  |  | CPIJ009409 | conserved hypothetical protein |
|  |  |  | CPIJ009502 | zinc finger protein |
|  |  |  | CPIJ009503 | zinc finger protein 583 |
|  |  |  | CPIJ009524 | forkhead box protein |
|  |  |  | CPIJ009647 | conserved hypothetical protein |
|  |  |  | CPIJ009780 | zinc finger protein 75A |
|  |  |  | CPIJ009786 | zinc finger protein |
|  |  |  | CPIJ009787 | zinc finger protein 582 |
|  |  |  | CPIJ009989 | zinc finger protein |
|  |  |  | CPIJ009990 | zinc finger protein |
|  |  |  | CPIJ010551 | predicted protein |
|  |  |  | CPIJ010652 | conserved hypothetical protein |
|  |  |  | CPIJ010850 | conserved hypothetical protein |
|  |  |  | CPIJ011015 | conserved hypothetical protein |
|  |  |  | CPIJ011166 | zinc finger protein |
|  |  |  | CPIJ011789 | conserved hypothetical protein |
|  |  |  | CPIJ012039 | conserved hypothetical protein |
|  |  |  | CPIJ012535 | zinc finger protein |
|  |  |  | CPIJ012594 | predicted protein |
|  |  |  | CPIJ012610 | zinc finger protein 38 |
|  |  |  | CPIJ013068 | zinc finger protein |
|  |  |  | CPIJ013118 | conserved hypothetical protein |
|  |  |  | CPIJ013246 | conserved hypothetical protein |
|  |  |  | CPIJ013653 | conserved hypothetical protein |
|  |  |  | CPIJ014029 | conserved hypothetical protein |
|  |  |  | CPIJ014036 | conserved hypothetical protein |
|  |  |  | CPIJ014135 | conserved hypothetical protein |
|  |  |  | CPIJ014711 | zinc finger protein |
|  |  |  | CPIJ014712 | krueppel protein |
|  |  |  | CPIJ014714 | zinc finger protein |
|  |  |  | CPIJ014715 | zinc finger protein |
|  |  |  | CPIJ015000 | zinc finger protein 250 |
|  |  |  | CPIJ015265 | zinc finger protein 345 |
|  |  |  | CPIJ015267 | zinc finger protein ZNF780A |
|  |  |  | CPIJ015425 | conserved hypothetical protein |
|  |  |  | CPIJ015578 | hypothetical protein |
|  |  |  | CPIJ015579 | conserved hypothetical protein |
|  |  |  | CPIJ015582 | conserved hypothetical protein |
|  |  |  | CPIJ016725 | conserved hypothetical protein |
|  |  |  | CPIJ016726 | conserved hypothetical protein |
|  |  |  | CPIJ016862 | conserved hypothetical protein |
|  |  |  | CPIJ016943 | conserved hypothetical protein |
|  |  |  | CPIJ016944 | conserved hypothetical protein |
|  |  |  | CPIJ017035 | zinc finger protein 141 |
|  |  |  | CPIJ017141 | conserved hypothetical protein |
|  |  |  | CPIJ017142 | conserved hypothetical protein |
|  |  |  | CPIJ017259 | predicted protein |
|  |  |  | CPIJ017278 | zinc finger protein 436 |
|  |  |  | CPIJ017355 | conserved hypothetical protein |
|  |  |  | CPIJ017654 | conserved hypothetical protein |
|  |  |  | CPIJ017756 | conserved hypothetical protein |
|  |  |  | CPIJ018074 | conserved hypothetical protein |
|  |  |  | CPIJ018336 | conserved hypothetical protein |
|  |  |  | CPIJ018423 | zinc finger protein |
|  |  |  | CPIJ018448 | transcription factor btd |
|  |  |  | CPIJ018505 | zinc finger transcription factor |
|  |  |  | CPIJ019181 | U1 small nuclear ribonucleoprotein C |
|  |  |  | CPIJ019417 | zinc finger protein 322A |
|  |  |  | CPIJ019621 | conserved hypothetical protein |
|  |  |  | CPIJ019710 | conserved hypothetical protein |
|  |  |  | CPIJ019775 | conserved hypothetical protein |
|  |  |  | CPIJ020207 | zinc finger protein 36 |
|  |  | CCCH zinc finger | CPIJ018734 | conserved hypothetical protein |
|  |  | CSL zinc finger | CPIJ001284 | conserved hypothetical protein |
|  |  | Cyclin-like | CPIJ006843 | transcription initiation factor TFIIB |
|  |  |  | CPIJ010939 | conserved hypothetical protein |
|  |  |  | CPIJ012914 | cyclin T |
|  |  |  | CPIJ013566 | cyclin a |
|  |  |  | CPIJ013567 | cyclin a |
|  |  | Cysteine-rich DNA binding domain, (DM domain) | CPIJ004057 | male-specific doublesex protein |
|  |  | DNA-binding domain | CPIJ004606 | phd finger domain |
|  |  | Glucocorticoid receptor-like (DNA-binding domain) | CPIJ002376 | elongation factor 1-alpha |
|  |  |  | CPIJ002547 | conserved hypothetical protein |
|  |  |  | CPIJ002808 | conserved hypothetical protein |
|  |  |  | CPIJ002811 | conserved hypothetical protein |
|  |  |  | CPIJ005912 | conserved hypothetical protein |
|  |  |  | CPIJ006674 | malate dehydrogenase |
|  |  |  | CPIJ006684 | predicted protein |
|  |  |  | CPIJ006830 | conserved hypothetical protein |
|  |  |  | CPIJ007349 | zinc finger protein 225 |
|  |  |  | CPIJ007701 | predicted protein |
|  |  |  | CPIJ008216 | nuclear hormone receptor ftz-f1 |
|  |  |  | CPIJ008348 | GATA transcription factor GATAd |
|  |  |  | CPIJ008682 | hypothetical protein |
|  |  |  | CPIJ009297 | conserved hypothetical protein |
|  |  |  | CPIJ009298 | conserved hypothetical protein |
|  |  |  | CPIJ009299 | four and a half lim domains |
|  |  |  | CPIJ010100 | conserved hypothetical protein |
|  |  |  | CPIJ010408 | conserved hypothetical protein |
|  |  |  | CPIJ010866 | conserved hypothetical protein |
|  |  |  | CPIJ011684 | conserved hypothetical protein |
|  |  |  | CPIJ011743 | conserved hypothetical protein |
|  |  |  | CPIJ012188 | epsilon-trimethyllysine 2-oxoglutarate dioxygenase |
|  |  |  | CPIJ012588 | predicted protein |
|  |  |  | CPIJ013614 | conserved hypothetical protein |
|  |  |  | CPIJ014027 | conserved hypothetical protein |
|  |  |  | CPIJ014086 | conserved hypothetical protein |
|  |  |  | CPIJ014594 | predicted protein |
|  |  |  | CPIJ016033 | GATA-binding factor-C |
|  |  |  | CPIJ016621 | conserved hypothetical protein |
|  |  |  | CPIJ017547 | conserved hypothetical protein |
|  |  |  | CPIJ018862 | retinoic acid receptor beta |
|  |  | Histone-fold | CPIJ001398 | transcription initiation factor TFIID subunit 9 |
|  |  |  | CPIJ008494 | histone h2a |
|  |  |  | CPIJ010882 | conserved hypothetical protein |
|  |  |  | CPIJ011778 | transcription initiation factor TFIID subunit 12 |
|  |  |  | CPIJ014768 | histone 1 |
|  |  |  | CPIJ017187 | histone H3.3 type 2 |
|  |  |  | CPIJ017276 | suppressor of ty3 |
|  |  |  | CPIJ018900 | conserved hypothetical protein |
|  |  | HIT/MYND zinc finger-like | CPIJ014434 | predicted protein |
|  |  |  | CPIJ017587 | conserved hypothetical protein |
|  |  | HLH, helix-loop-helix DNA-binding domain | CPIJ002332 | conserved hypothetical protein |
|  |  |  | CPIJ003409 | enhancer of split mgamma protein |
|  |  |  | CPIJ007015 | conserved hypothetical protein |
|  |  |  | CPIJ008120 | conserved hypothetical protein |
|  |  |  | CPIJ012827 | conserved hypothetical protein |
|  |  |  | CPIJ015080 | conserved hypothetical protein |
|  |  |  | CPIJ015473 | max binding protein |
|  |  | HMG-box | CPIJ001997 | conserved hypothetical protein |
|  |  |  | CPIJ005084 | coiled-coil domain-containing protein 124 |
|  |  |  | CPIJ006395 | conserved hypothetical protein |
|  |  |  | CPIJ012202 | capicua protein |
|  |  |  | CPIJ014423 | conserved hypothetical protein |
|  |  |  | CPIJ014424 | conserved hypothetical protein |
|  |  |  | CPIJ017659 | conserved hypothetical protein |
|  |  | Homeodomain-like | CPIJ001021 | homeobox protein abdominal-B |
|  |  |  | CPIJ002815 | conserved hypothetical protein |
|  |  |  | CPIJ005379 | metastasis-associated protein 3 |
|  |  |  | CPIJ005827 | predicted protein |
|  |  |  | CPIJ006382 | paired box protein pax-6 |
|  |  |  | CPIJ006390 | paired box protein Pax-6 |
|  |  |  | CPIJ008039 | homeobox protein extradenticle |
|  |  |  | CPIJ009982 | rest corepressor protein |
|  |  |  | CPIJ010220 | segmentation polarity homeobox protein engrailed |
|  |  |  | CPIJ012080 | conserved hypothetical protein |
|  |  |  | CPIJ012784 | zinc finger protein 1 |
|  |  |  | CPIJ014669 | predicted protein |
|  |  |  | CPIJ015889 | homeobox protein |
|  |  |  | CPIJ017153 | conserved hypothetical protein |
|  |  |  | CPIJ017214 | mesoderm induction early response protein 1 |
|  |  |  | CPIJ019460 | hypothetical protein |
|  |  | Insert subdomain of RNA polymerase alpha subunit | CPIJ003123 | DNA-directed RNA polymerase I 40 kDa polypeptide |
|  |  | Kix domain of CBP (creb binding protein) | CPIJ005540 | conserved hypothetical protein |
|  |  | lambda repressor-like DNA-binding domains | CPIJ003986 | multiprotein bridging factor |
|  |  |  | CPIJ014526 | conserved hypothetical protein |
|  |  | Leucine zipper domain | CPIJ003266 | CCAAT/enhancer-binding protein |
|  |  |  | CPIJ003767 | conserved hypothetical protein |
|  |  |  | CPIJ003805 | cyclic-AMP response element binding protein |
|  |  |  | CPIJ012178 | ovary C/EBPg transcription factor |
|  |  |  | CPIJ014920 | par domain protein |
|  |  |  | CPIJ016941 | conserved hypothetical protein |
|  |  | p53-like transcription factors | CPIJ000431 | conserved hypothetical protein |
|  |  |  | CPIJ000433 | T-box protein H15 |
|  |  |  | CPIJ000721 | conserved hypothetical protein |
|  |  |  | CPIJ002764 | conserved hypothetical protein |
|  |  |  | CPIJ007738 | T-box protein H15 |
|  |  |  | CPIJ016469 | signal transducer and activator of transcription |
|  |  | Periplasmic binding protein-like I | CPIJ010082 | atrial natriuretic peptide receptor |
|  |  |  | CPIJ019599 | glutamate receptor, ionotropic kainate 1, 2, 3 |
|  |  |  | CPIJ020040 | conserved hypothetical protein |
|  |  | Putative DNA-binding domain | CPIJ000580 | ladybird homeobox corepressor |
|  |  |  | CPIJ000978 | transforming protein Ski |
|  |  | RPB6/omega subunit-like | CPIJ018444 | DNA-directed RNA polymeraseI |
|  |  | SAM/Pointed domain | CPIJ000845 | conserved hypothetical protein |
|  |  |  | CPIJ000860 | conserved hypothetical protein |
|  |  |  | CPIJ009010 | conserved hypothetical protein |
|  |  |  | CPIJ017757 | conserved hypothetical protein |
|  |  | SAP domain | CPIJ007511 | conserved hypothetical protein |
|  |  | SMAD MH1 domain | CPIJ009526 | nuclear factor i |
|  |  | SMAD/FHA domain | CPIJ000274 | conserved hypothetical protein |
|  |  |  | CPIJ006464 | nuclear inhibitor of protein phosphatase 1 |
|  |  |  | CPIJ006834 | kinesin-like protein KIF1B |
|  |  |  | CPIJ009516 | conserved hypothetical protein |
|  |  | SRF-like | CPIJ008335 | conserved hypothetical protein |
|  |  |  | CPIJ016459 | conserved hypothetical protein |
|  |  | Tim10-like | CPIJ018054 | mitochondrial import inner membrane translocase subunit Tim8 A |
|  |  |  | CPIJ010382 | mitochondrial import inner membrane translocase subunit Tim10 |
|  |  |  | CPIJ010840 | mitochondrial inner membrane protein translocase, 9kD-subunit |
|  |  |  | CPIJ018053 | mitochondrial inner membrane protein translocase, 8kD-subunit |
|  |  |  | CPIJ018211 | mitochondrial inner membrane protein translocase, 13kD-subunit |
|  |  | Winged helix DNA-binding domain | CPIJ001680 | DNA-binding protein D-ELG |
|  |  |  | CPIJ001711 | conserved hypothetical protein |
|  |  |  | CPIJ003068 | conserved hypothetical protein |
|  |  |  | CPIJ003923 | conserved hypothetical protein |
|  |  |  | CPIJ004011 | rfx transcription factor |
|  |  |  | CPIJ009522 | conserved hypothetical protein |
|  |  |  | CPIJ010292 | vacuolar protein sorting-associated protein 25 |
|  |  |  | CPIJ013475 | transcription initiation factor IIE subunit beta |
|  |  |  | CPIJ014720 | ets DNA-binding protein pokkuri |
|  |  |  | CPIJ015971 | 26S proteasome non-ATPase regulatory subunit 11 |
|  | Kinases/phosphatases | (Phosphotyrosine protein) phosphatases II | CPIJ018450 | slingshot dual specificity phosphatase |
|  |  |  | CPIJ019559 | phosphatase Slingshot |
|  |  |  | CPIJ001969 | tyrosine phosphatase prl |
|  |  |  | CPIJ003757 | tyrosine phosphatase mitochondrial 1 |
|  |  |  | CPIJ008018 | dual specificity protein phosphatase |
|  |  |  | CPIJ008808 | conserved hypothetical protein |
|  |  |  | CPIJ009405 | testis/ seletal muscle dual specificty phosphatase |
|  |  |  | CPIJ011976 | tyrosine phosphatase, non-receptor type nt1 |
|  |  |  | CPIJ013410 | tyrosine-protein phosphatase Lar |
|  |  |  | CPIJ014898 | tyrosine phosphatase n11 |
|  |  |  | CPIJ018298 | tryrosine phosphatase |
|  |  | FAT domain of focal adhesion kinase | CPIJ017572 | focal adhesion kinase |
|  |  | GHMP Kinase, C-terminal domain | CPIJ015185 | conserved hypothetical protein |
|  |  | Myosin phosphatase inhibitor 17kDa protein, CPI-17 | CPIJ004356 | conserved hypothetical protein |
|  |  | Phosphotyrosine protein phosphatases I | CPIJ008291 | low molecular weight phosphotyrosine protein phosphatase 1 |
|  |  | PP2C-like | CPIJ012255 | pyruvate dehydrogenase |
|  |  | Protein kinase-like (PK-like) | CPIJ010275 | map/microtubule affinity-regulating kinase 2,4 |
|  |  |  | CPIJ000270 | tyrosine kinase |
|  |  |  | CPIJ000288 | serine/threonine-protein kinase 3 |
|  |  |  | CPIJ000816 | kinase protein |
|  |  |  | CPIJ000833 | tyrosine-protein kinase btk29a |
|  |  |  | CPIJ000891 | cell division protein kinase 8 |
|  |  |  | CPIJ001155 | cell division protein kinase 2 |
|  |  |  | CPIJ001273 | conserved hypothetical protein |
|  |  |  | CPIJ003568 | mitosis inhibitor protein kinase |
|  |  |  | CPIJ003599 | serine/threonine protein kinase |
|  |  |  | CPIJ003985 | tyrosine-protein kinase Abl |
|  |  |  | CPIJ003996 | calcium-dependent protein kinase |
|  |  |  | CPIJ004173 | Juvenile hormone-inducible protein |
|  |  |  | CPIJ004685 | Dual specificity tyrosine-phosphorylation-regulated kinase |
|  |  |  | CPIJ004687 | Dual specificity tyrosine-phosphorylation-regulated kinase |
|  |  |  | CPIJ004799 | activin receptor type I |
|  |  |  | CPIJ004910 | serine/threonine kinase NLK |
|  |  |  | CPIJ005276 | cGMP-protein kinase |
|  |  |  | CPIJ005290 | conserved hypothetical protein |
|  |  |  | CPIJ005449 | nuclear body associated kinase |
|  |  |  | CPIJ006283 | ser/thr protein kinase-trb3 |
|  |  |  | CPIJ006284 | ser/thr protein kinase-trb3 |
|  |  |  | CPIJ006704 | serine/threonine-protein kinase D3 |
|  |  |  | CPIJ007227 | tyrosine-protein kinase |
|  |  |  | CPIJ007458 | tyrosine-protein kinase src64b |
|  |  |  | CPIJ008931 | dual specificity mitogen-activated protein kinase kinase hemipterous |
|  |  |  | CPIJ008953 | mitogen-activated protein kinase kinase kinase |
|  |  |  | CPIJ009012 | mixed lineage protein kinase |
|  |  |  | CPIJ009223 | serine/threonine-protein kinase |
|  |  |  | CPIJ010322 | cell division control protein |
|  |  |  | CPIJ011073 | discoidin domain receptor |
|  |  |  | CPIJ011673 | cell division control protein |
|  |  |  | CPIJ011936 | S6 kinase II beta |
|  |  |  | CPIJ012176 | ribosomal protein S6 kinase, 90kD, polypeptide |
|  |  |  | CPIJ012534 | eukaryotic translation initiation factor 2-alpha kinase 1 |
|  |  |  | CPIJ012560 | leucine-rich repeat serine/threonine-protein kinase 1 |
|  |  |  | CPIJ013693 | serine/threonine-protein kinase rio2 |
|  |  |  | CPIJ013835 | conserved hypothetical protein |
|  |  |  | CPIJ013942 | conserved hypothetical protein |
|  |  |  | CPIJ014803 | Dual specificity tyrosine-phosphorylation-regulated kinase |
|  |  |  | CPIJ015689 | serine/threonine-protein kinase rio2 |
|  |  |  | CPIJ015690 | serine/threonine-protein kinase RIO2 |
|  |  |  | CPIJ015801 | mitogen activated protein kinase kinase 2 |
|  |  |  | CPIJ015833 | map/microtubule affinity-regulating kinase 2,4 |
|  |  |  | CPIJ015918 | conserved hypothetical protein |
|  |  |  | CPIJ015922 | Juvenile hormone-inducible protein |
|  |  |  | CPIJ016475 | conserved hypothetical protein |
|  |  |  | CPIJ016644 | integrin-linked protein kinase |
|  |  |  | CPIJ016729 | serine/threonine-protein kinase vrk |
|  |  |  | CPIJ016868 | conserved hypothetical protein |
|  |  |  | CPIJ018008 | fibroblast growth factor receptor |
|  |  |  | CPIJ018201 | serine/threonine protein kinase lats |
|  |  |  | CPIJ019408 | conserved hypothetical protein |
|  |  |  | CPIJ019493 | fibroblast growth factor receptor 1 |
|  |  |  | CPIJ019683 | conserved hypothetical protein |
|  | Other regulatory function | GCM domain | CPIJ006640 | conserved hypothetical protein |
|  |  | Mago nashi protein | CPIJ002949 | mago nashi |
|  |  | Mob1/phocein | CPIJ002914 | conserved hypothetical protein |
|  |  | N-terminal domain of adenylylcyclase associated protein, CAP | CPIJ003125 | adenylyl cyclase-associated protein |
|  |  | Ran BP2/NZF zinc finger-like | CPIJ003010 | conserved hypothetical protein |
|  |  |  | CPIJ007177 | nucleoporin, Nup153 |
|  |  | Sec7 domain | CPIJ002812 | guanyl-nucleotide exchange factor |
|  |  |  | CPIJ015585 | arf6 guanine nucleotide exchange factor |
|  | Receptor activity | Chemosensory protein Csp2 | CPIJ019986 | serine/threonine kinase |
|  |  |  | CPIJ002601 | conserved hypothetical protein |
|  |  |  | CPIJ002605 | serine/threonine kinase |
|  |  |  | CPIJ002607 | conserved hypothetical protein |
|  |  |  | CPIJ002608 | chemosensory protein |
|  |  |  | CPIJ002609 | serine/threonine kinase |
|  |  |  | CPIJ002616 | serine/threonine kinase |
|  |  |  | CPIJ002625 | chemosensory protein 1 |
|  |  |  | CPIJ002629 | sensory appendage protein |
|  |  |  | CPIJ017094 | serine/threonine kinase |
|  |  |  | CPIJ019985 | sensory appendage protein |
|  | RNA binding, m/tr | Alpha-L RNA-binding motif | CPIJ016140 | tyrosyl-tRNA synthetase |
|  |  | dsRNA-binding domain-like | CPIJ003264 | 40S ribosomal protein S2 |
|  |  |  | CPIJ004832 | tar RNA binding protein |
|  |  |  | CPIJ006845 | 40S ribosomal protein S2 |
|  |  |  | CPIJ008332 | 40S ribosomal protein S2 |
|  |  |  | CPIJ009774 | 40S ribosomal protein S2 |
|  |  |  | CPIJ011850 | double-stranded RNA-specific editase Adar |
|  |  |  | CPIJ013506 | ATP-dependent RNA helicase |
|  |  |  | CPIJ014041 | conserved hypothetical protein |
|  |  | Nop domain | CPIJ002179 | U4/U6 small nuclear ribonucleoprotein Prp31 |
|  |  | Nop10-like SnoRNP | CPIJ018833 | H/ACA ribonucleoprotein complex subunit 3 |
|  |  | PUA domain-like | CPIJ006069 | adenylsulfate kinase |
|  |  |  | CPIJ008799 | ATP-dependent Lon protease |
|  |  |  | CPIJ012137 | conserved hypothetical protein |
|  |  | RNA-binding domain, RBD | CPIJ014704 | splicing factor |
|  |  |  | CPIJ019416 | splicing factor |
|  |  |  | CPIJ000025 | negative elongation factor E |
|  |  |  | CPIJ000485 | conserved hypothetical protein |
|  |  |  | CPIJ000588 | polypyrimidine tract binding protein |
|  |  |  | CPIJ000880 | RNA-binding post-transcriptional regulator csx1 |
|  |  |  | CPIJ001612 | heterogeneous nuclear ribonucleoprotein |
|  |  |  | CPIJ003074 | conserved hypothetical protein |
|  |  |  | CPIJ003555 | nuclear cap-binding protein subunit 2 |
|  |  |  | CPIJ003738 | heterogeneous nuclear ribonucleoprotein r |
|  |  |  | CPIJ003854 | conserved hypothetical protein |
|  |  |  | CPIJ003881 | G-rich sequence factor-1 |
|  |  |  | CPIJ004538 | conserved hypothetical protein |
|  |  |  | CPIJ004892 | conserved hypothetical protein |
|  |  |  | CPIJ005653 | developmentally regulated RNA-binding protein |
|  |  |  | CPIJ006107 | NONA protein |
|  |  |  | CPIJ006979 | polypyrimidine tract binding protein |
|  |  |  | CPIJ007047 | serine/arginine rich splicing factor |
|  |  |  | CPIJ007295 | conserved hypothetical protein |
|  |  |  | CPIJ007629 | splicing factor |
|  |  |  | CPIJ007834 | RNA and export factor binding protein |
|  |  |  | CPIJ008248 | conserved hypothetical protein |
|  |  |  | CPIJ008476 | conserved hypothetical protein |
|  |  |  | CPIJ008628 | predicted protein |
|  |  |  | CPIJ008634 | 52K active chromatin boundary protein |
|  |  |  | CPIJ008698 | rbm25 protein |
|  |  |  | CPIJ008786 | arginine/serine-rich splicing factor |
|  |  |  | CPIJ009773 | conserved hypothetical protein |
|  |  |  | CPIJ012174 | conserved hypothetical protein |
|  |  |  | CPIJ012515 | conserved hypothetical protein |
|  |  |  | CPIJ012662 | cleavage stimulation factor 64 kDa subunit |
|  |  |  | CPIJ012814 | scaffold attachment factor b |
|  |  |  | CPIJ012850 | splicing factor u2af large subunit |
|  |  |  | CPIJ013237 | conserved hypothetical protein |
|  |  |  | CPIJ014506 | fuse-binding protein-interacting repressor siahbp1 |
|  |  |  | CPIJ015052 | RNA binding motif protein 18 |
|  |  |  | CPIJ015262 | heterogeneous nuclear ribonucleoprotein 27C |
|  |  |  | CPIJ015350 | eukaryotic translation initiation factor 3 subunit 4 |
|  |  |  | CPIJ015549 | ribosomal biogenesis protein Gar2 |
|  |  |  | CPIJ016143 | conserved hypothetical protein |
|  |  |  | CPIJ016329 | conserved hypothetical protein |
|  |  |  | CPIJ016517 | conserved hypothetical protein |
|  |  |  | CPIJ017755 | conserved hypothetical protein |
|  |  |  | CPIJ018451 | conserved hypothetical protein |
|  |  | Surp module (SWAP domain) | CPIJ017179 | scaffold attachment factor B |
|  | Signal transduction | C2 domain (Calcium/lipid-binding domain, CaLB) | CPIJ005008 | E3 ubiquitin ligase |
|  |  |  | CPIJ005701 | kinase C alpha-polypeptide |
|  |  |  | CPIJ011785 | E3 ubiquitin-protein ligase nedd-4 |
|  |  |  | CPIJ015045 | conserved hypothetical protein |
|  |  |  | CPIJ015112 | E3 ubiquitin-protein ligase nedd-4 |
|  |  |  | CPIJ016865 | conserved hypothetical protein |
|  |  | cAMP-binding domain-like | CPIJ004551 | conserved hypothetical protein |
|  |  |  | CPIJ005277 | conserved hypothetical protein |
|  |  |  | CPIJ005279 | cGMP-dependent protein kinase |
|  |  |  | CPIJ006213 | cyclic nucleotide-gated cation channel 4 |
|  |  |  | CPIJ015446 | cyclic-nucleotide-gated cation channel |
|  |  |  | CPIJ015653 | cGMP-dependent protein kinase |
|  |  |  | CPIJ015942 | c-AMP dependent protein kinase typeI-beta regulatory subunit |
|  |  |  | CPIJ018420 | conserved hypothetical protein |
|  |  |  | CPIJ019876 | cyclic nucleotide-gated cation channel beta 3 |
|  |  | DBL homology domain (DH-domain) | CPIJ001482 | pak-interacting exchange factor, beta-pix/cool-1 |
|  |  |  | CPIJ004982 | rho guanine exchange factor |
|  |  |  | CPIJ011504 | RHO guanyl-nucleotide exchange factor |
|  |  |  | CPIJ013987 | conserved hypothetical protein |
|  |  |  | CPIJ015421 | Rho GEF and pleckstrin domain protein |
|  |  |  | CPIJ017598 | guanine nucleotide exchange factor |
|  |  | Family A G protein-coupled receptor-like | CPIJ006268 | cardioacceleratory peptide receptor |
|  |  |  | CPIJ006269 | cardioacceleratory peptide receptor |
|  |  |  | CPIJ011619 | leucine-rich transmembrane protein |
|  |  |  | CPIJ014487 | beta adrenergic receptor |
|  |  |  | CPIJ014753 | conserved hypothetical protein |
|  |  |  | CPIJ015979 | conserved hypothetical protein |
|  |  |  | CPIJ016092 | adenosine A2 receptor |
|  |  | Frizzled cysteine-rich domain | CPIJ011799 | conserved hypothetical protein |
|  |  | Growth factor receptor domain | CPIJ009117 | conserved hypothetical protein |
|  |  |  | CPIJ015183 | proprotein convertase subtilisin/kexin type 4, furin |
|  |  |  | CPIJ017374 | conserved hypothetical protein |
|  |  | GTPase activation domain, GAP | CPIJ010879 | conserved hypothetical protein |
|  |  |  | CPIJ012517 | cdc42 GTPase-activating protein |
|  |  |  | CPIJ015825 | conserved hypothetical protein |
|  |  |  | CPIJ019727 | conserved hypothetical protein |
|  |  | Insect pheromone/odorant-binding proteins | CPIJ001867 | hypothetical protein |
|  |  |  | CPIJ001871 | hypothetical protein |
|  |  |  | CPIJ001874 | Odorant-binding protein 56a |
|  |  |  | CPIJ003865 | conserved hypothetical protein |
|  |  |  | CPIJ003866 | conserved hypothetical protein |
|  |  |  | CPIJ004634 | odorant-binding protein |
|  |  |  | CPIJ004635 | odorant-binding protein OBPjj7a |
|  |  |  | CPIJ007337 | conserved hypothetical protein |
|  |  |  | CPIJ010787 | conserved hypothetical protein |
|  |  |  | CPIJ010788 | conserved hypothetical protein |
|  |  |  | CPIJ018881 | tRNA delta |
|  |  | Insulin-like | CPIJ018049 | conserved hypothetical protein |
|  |  | Nicotinic receptor ligand binding domain-like | CPIJ007639 | acetylcholine receptor protein alpha 1, 2, 3, 4 invertebrate |
|  |  |  | CPIJ016909 | nicotinic acetylcholine receptor, beta-2 subunit |
|  |  | Nuclear receptor ligand-binding domain | CPIJ002963 | ecdysone receptor |
|  |  |  | CPIJ004609 | nuclear hormone receptor |
|  |  |  | CPIJ008215 | nuclear hormone receptor ftz-f1 |
|  |  |  | CPIJ009588 | conserved hypothetical protein |
|  |  |  | CPIJ014945 | nuclear hormone receptor ftz-f1 |
|  |  |  | CPIJ015542 | nuclear receptor 3 |
|  |  |  | CPIJ016024 | retinoid x receptor |
|  |  | Nucleotide cyclase | CPIJ004739 | adenylate cyclase |
|  |  |  | CPIJ015189 | adenylate cyclase |
|  |  |  | CPIJ017081 | guanylate cyclase soluble subunit beta-1 |
|  |  |  | CPIJ017287 | adenylate cyclase type |
|  |  |  | CPIJ019946 | adenylate cyclase type 5 |
|  |  | PDZ domain-like | CPIJ001710 | conserved hypothetical protein |
|  |  |  | CPIJ003808 | partitioning defective 3 |
|  |  |  | CPIJ004495 | conserved hypothetical protein |
|  |  |  | CPIJ005564 | 26S proteasome non-ATPase regulatory subunit 9 |
|  |  |  | CPIJ006020 | conserved hypothetical protein |
|  |  |  | CPIJ006377 | conserved hypothetical protein |
|  |  |  | CPIJ007358 | conserved hypothetical protein |
|  |  |  | CPIJ007684 | conserved hypothetical protein |
|  |  |  | CPIJ010875 | conserved hypothetical protein |
|  |  |  | CPIJ015012 | conserved hypothetical protein |
|  |  |  | CPIJ016071 | golgi reassembly-stacking protein 2 |
|  |  |  | CPIJ018273 | conserved hypothetical protein |
|  |  |  | CPIJ018340 | ezrin-radixin-moesin-binding phosphoprotein 50 |
|  |  |  | CPIJ019226 | Glutamate receptor binding protein |
|  |  |  | CPIJ019766 | conserved hypothetical protein |
|  |  | PH domain-like | CPIJ000361 | conserved hypothetical protein |
|  |  |  | CPIJ004690 | numb protein |
|  |  |  | CPIJ004706 | vasodilator-stimulated phosphoprotein |
|  |  |  | CPIJ005592 | decapping protein 1 |
|  |  |  | CPIJ005688 | conserved hypothetical protein |
|  |  |  | CPIJ006699 | wiskott-aldrich syndrome protein |
|  |  |  | CPIJ007927 | conserved hypothetical protein |
|  |  |  | CPIJ009368 | conserved hypothetical protein |
|  |  |  | CPIJ009993 | conserved hypothetical protein |
|  |  |  | CPIJ010369 | conserved hypothetical protein |
|  |  |  | CPIJ010878 | conserved hypothetical protein |
|  |  |  | CPIJ011071 | structure-specific recognition protein |
|  |  |  | CPIJ011748 | conserved hypothetical protein |
|  |  |  | CPIJ012737 | nucleoporin 50kDa |
|  |  |  | CPIJ013583 | conserved hypothetical protein |
|  |  |  | CPIJ013686 | signal transduction protein lnk-realted |
|  |  |  | CPIJ013709 | conserved hypothetical protein |
|  |  |  | CPIJ013938 | conserved hypothetical protein |
|  |  |  | CPIJ018995 | conserved hypothetical protein |
|  |  |  | CPIJ019612 | myosin xv |
|  |  |  | CPIJ019728 | conserved hypothetical protein |
|  |  |  | CPIJ019740 | conserved hypothetical protein |
|  |  |  | CPIJ020011 | FACT complex subunit Ssrp1 |
|  |  | PX domain | CPIJ001623 | sorting nexin |
|  |  |  | CPIJ005342 | sorting nexin-6 |
|  |  |  | CPIJ006689 | conserved hypothetical protein |
|  |  |  | CPIJ010119 | sorting nexin-9 |
|  |  |  | CPIJ013306 | conserved hypothetical protein |
|  |  | PYP-like sensor domain (PAS domain) | CPIJ003682 | hypoxia-inducible factor |
|  |  |  | CPIJ013448 | conserved hypothetical protein |
|  |  |  | CPIJ014773 | arylhydrocarbon receptor nuclear translocator |
|  |  | Rap/Ran-GAP | CPIJ018142 | rap GTPase-activating protein |
|  |  | Ras GEF | CPIJ005593 | ras GTP exchange factor, son of sevenless |
|  |  |  | CPIJ005901 | ral guanine nucleotide exchange factor 2 |
|  |  |  | CPIJ017680 | c-AMP-dependent rap1 guanine-nucleotide exchange factor |
|  |  | Regulator of G-protein signaling, RGS | CPIJ004658 | beta-adrenergic receptor kinase |
|  |  |  | CPIJ006996 | conserved hypothetical protein |
|  |  |  | CPIJ015931 | regulator of g protein signaling |
|  |  | SH2 domain | CPIJ000806 | growth factor receptor-bound protein |
|  |  |  | CPIJ003100 | cytoplasmic protein NCK1 |
|  |  |  | CPIJ003380 | suppressorsof cytokine signalling |
|  |  |  | CPIJ010659 | proto-oncogene tyrosine-protein kinase src |
|  |  |  | CPIJ012129 | conserved hypothetical protein |
|  |  |  | CPIJ014900 | corkscrew phosphatase |
|  |  | SH3-domain | CPIJ006389 | abl interactor 2 |
|  |  |  | CPIJ002311 | conserved hypothetical protein |
|  |  |  | CPIJ002634 | membrane traffic protein |
|  |  |  | CPIJ003002 | nebl protein |
|  |  |  | CPIJ006223 | Plenty of SH3s |
|  |  |  | CPIJ010657 | conserved hypothetical protein |
|  |  |  | CPIJ016081 | endophilin a |
|  |  |  | CPIJ017698 | conserved hypothetical protein |
|  |  |  | CPIJ018548 | abl interactor 2 |
|  |  | TRAF domain-like | CPIJ001208 | autoimmune regulator |
|  |  |  | CPIJ001213 | tripartite motif-containing protein 37 |
|  |  |  | CPIJ009247 | E3 ubiquitin-protein ligase sina |
|  |  |  | CPIJ010192 | conserved hypothetical protein |
|  |  | Transducin (alpha subunit), insertion domain | CPIJ016397 | guanine nucleotide-binding protein G |
|  |  | Transducin (heterotrimeric G protein), gamma chain | CPIJ005863 | guanine nucleotide-binding protein gamma-1 subunit |
|  |  | Ypt/Rab-GAP domain of gyp1p | CPIJ016901 | conserved hypothetical protein |
|  |  |  | CPIJ008317 | TBC1 domain family member 22B |
|  |  |  | CPIJ010266 | GTPase-activating protein gyp2 |
|  |  |  | CPIJ011634 | gh regulated tbc protein-1 |
| Other | Unknown function | alpha/beta knot | CPIJ006883 | conserved hypothetical protein |
|  |  |  | CPIJ010464 | conserved hypothetical protein |
|  |  | Anti-sigma factor antagonist SpoIIaa | CPIJ006006 | sulfate transporter |
|  |  |  | CPIJ000611 | sulfate transporter 1.2 |
|  |  |  | CPIJ005331 | sulfate transporter |
|  |  |  | CPIJ012147 | sulfate transporter |
|  |  |  | CPIJ017095 | sulfate transporter |
|  |  | beta-sandwich domain of Sec23/24 | CPIJ008805 | conserved hypothetical protein |
|  |  |  | CPIJ009281 | Sec24B protein |
|  |  |  | CPIJ014598 | conserved hypothetical protein |
|  |  |  | CPIJ017651 | conserved hypothetical protein |
|  |  | BtrG-like | CPIJ005346 | conserved hypothetical protein |
|  |  |  | CPIJ008797 | conserved hypothetical protein |
|  |  | Crustacean CHH/MIH/GIH neurohormone | CPIJ003972 | ion transport peptide |
|  |  | Cysteine alpha-hairpin motif | CPIJ000336 | conserved hypothetical protein |
|  |  |  | CPIJ014050 | predicted protein |
|  |  |  | CPIJ019817 | cytochrome c oxidase assembly protein COX19 |
|  |  | Cysteine-rich domain | CPIJ000792 | myotonin-protein kinase |
|  |  |  | CPIJ006705 | protein kinase C |
|  |  |  | CPIJ006920 | conserved hypothetical protein |
|  |  | Delta-sleep-inducing peptide immunoreactive peptide | CPIJ006273 | conserved hypothetical protein |
|  |  | E set domains | CPIJ002638 | KDEL motif-containing protein 2 |
|  |  |  | CPIJ002736 | conserved hypothetical protein |
|  |  |  | CPIJ002737 | MPA2 allergen |
|  |  |  | CPIJ004282 | hexamerin 2 beta |
|  |  |  | CPIJ004652 | conserved hypothetical protein |
|  |  |  | CPIJ005750 | conserved hypothetical protein |
|  |  |  | CPIJ008373 | conserved hypothetical protein |
|  |  |  | CPIJ016072 | SEC63 protein |
|  |  |  | CPIJ016988 | beta-arrestin 1 |
|  |  | Frataxin/Nqo15-like | CPIJ014024 | frataxin, mitochondrial |
|  |  | GckA/TtuD-like | CPIJ004803 | glycerate kinase |
|  |  | Hairpin loop containing domain-like | CPIJ005068 | conserved hypothetical protein |
|  |  |  | CPIJ005070 | conserved hypothetical protein |
|  |  |  | CPIJ018970 | conserved hypothetical protein |
|  |  | HCP-like | CPIJ001345 | conserved hypothetical protein |
|  |  | Hook domain | CPIJ018678 | hook protein |
|  |  | Ligand-binding domain in the NO signalling and Golgi transport | CPIJ010765 | conserved hypothetical protein |
|  |  |  | CPIJ019438 | conserved hypothetical protein |
|  |  | MAL13P1.257-like | CPIJ013731 | conserved hypothetical protein |
|  |  | PIN domain-like | CPIJ018772 | conserved hypothetical protein |
|  |  | PTPA-like | CPIJ017735 | serine/threonine-protein phosphatase 2A regulatory subunitB' |
|  |  | Pym (Within the bgcn gene intron protein, WIBG), N-terminal domain | CPIJ006916 | conserved hypothetical protein |
|  |  | Roadblock/LC7 domain | CPIJ002048 | dynein light chain |
|  |  |  | CPIJ011264 | mitogen-activated protein-binding protein-interacting protein |
|  |  |  | CPIJ014786 | conserved hypothetical protein |
|  |  | Subunits of heterodimeric actin filament capping protein Capz | CPIJ010373 | F-actin capping protein subunit beta |
|  |  |  | CPIJ011271 | f-actin capping protein alpha |
|  |  |  | CPIJ011272 | conserved hypothetical protein |
|  |  |  | CPIJ019319 | F-actin capping protein subunit beta |
|  |  | YggU-like | CPIJ001196 | conserved hypothetical protein |
|  |  | YjeF N-terminal domain-like | CPIJ011786 | conserved hypothetical protein |
|  |  | YjgF-like | CPIJ008399 | conserved hypothetical protein |
|  |  | Zinc beta-ribbon | CPIJ001855 | DNA-directed RNA polymerase II 15.1 kDa polypeptide |
|  | Viral proteins | Arp2/3 complex 16 kDa subunit ARPC5 | CPIJ012795 | arp2/3 complex 16 kd subunit |
|  |  | Retrovirus zinc finger-like domains | CPIJ001891 | conserved hypothetical protein |
|  |  | Tetrapyrrole methylase | CPIJ008675 | diphthine synthase |
|  |  | Eferin C-derminal domain-like | CPIJ003754 | conserved hypothetical protein |
|  |  | ERH-like | CPIJ013494 | enhancer of rudimentary protein |
|  |  | Expressed protein At2g23090/F21P24.15 | CPIJ002299 | conserved hypothetical protein |

^†^Differentially expressed genes represent those genes that differed in their expression level (FPKM) in HAmCq^G8^ by more than two fold when compared to the parental strain HAmCq^G0^.

^*^SCOP general and detailed functions using the predicted *Cx. quinquefasciatus* annotation information available at the Superfamily website (version 1.75) supfam.cs.bris.ac.uk/SUPERFAMILY/index.html

^**^*Culex quinquefasciatus* genome, Johannesburg strain CpipJ1.2, June 2008; http://cquinquefasciatus.vectorbase.org/

^‡^Vectorbase annotation taken from CpipJ1.2, June 2008; http://cquinquefasciatus.vectorbase.org/ with the exception of cytochrome P450 genes whose annotations were taken from the most current P450 annotation based on: Nelson, DR (2009) The Cytochrome P450 Homepage. Human Genomics 4, 59-65: http://drnelson.uthsc.edu/CytochromeP450.html

^§^NONA: Not annotated
