# Supplementary material for: The Transcriptome Profile of the Mosquito Culex quinquefasciatus following Permethrin Selection
Source: PLoS One. 2012 Oct 5;7(10):e47163. doi: 10.1371/journal.pone.0047163 (PMC3465273; doi:10.1371/journal.pone.0047163)
Supplement: Table S5 — List of differentially upregulated genes in HAmCqG8 which contained functionally-enriched Gene Ontology terms. (DOC) [file pone.0047163.s005.doc]

Table S5: List of differentially upregulated genes in HAmCq^G8^ which contained functionally-enriched Gene Ontology terms.

| Gene^†^ | Annotation^*^ | GO TERMS^**^ | | | | | | | | | | | | | | | | | | |
| --- | --- | --- | --- | --- | --- | --- | --- | --- | --- | --- | --- | --- | --- | --- | --- | --- | --- | --- | --- | --- |
|  |  | catalytic activity (GO:0003824) | oxidoreductase activity (GO:001649) | heme binding (GO:0020037) | tetrapyrrole binding (GO:0046906) | iron ion binding (GO: 0005506) | electron carrier activity (GO:009505) | monooxygenase activity (GO:004497) | hydrolase activity (GO:0016787) | peptidase activity (GO:0008233) | peptidase activity, acting on L-amino acid peptides (GO:0070011) | endopeptidase activity (GO:0004175) | serine-type endopeptidase activity (GO:0004252) | serine-type peptidase activity (GO: 0008236) | serine hydrolase activity (GO:0017171) | metallopeptidase activity (GO:0008237) | exopeptidase activity (GO:0008238) | hydrolase activity, acting on glycosyl bonds (GO:0008238) | hydrolase activity, hydrolyzing O-glycosyl compounds (GO:0004553) | oxygen transporter activity (GO:0005322) |
| CPIJ020229 | *CYP4D42v2* | **+^‡^** | **+** | **+** | **+** | **+** | **+** | **+** | - | - | - | - | - | - | - | - | - | - | - | - |
| CPIJ017244 | *CYP304B5* | **+** | **+** | **+** | **+** | **+** | **+** | **+** | - | - | - | - | - | - | - | - | - | - | - | - |
| CPIJ017243 | *CYP304B4* | **+** | **+** | **+** | **+** | **+** | **+** | **+** | - | - | - | - | - | - | - | - | - | - | - | - |
| CPIJ015958 | *CYP325BC1* | **+** | **+** | **+** | **+** | **+** | **+** | **+** | - | - | - | - | - | - | - | - | - | - | - | - |
| CPIJ015681 | *CYP4H37v2* | **+** | **+** | **+** | **+** | **+** | **+** | **+** | - | - | - | - | - | - | - | - | - | - | - | - |
| CPIJ014218 | *CYP9M10* | **+** | **+** | **+** | **+** | **+** | **+** | **+** | - | - | - | - | - | - | - | - | - | - | - | - |
| CPIJ012470 | *CYP9AL1* | **+** | **+** | **+** | **+** | **+** | **+** | **+** | - | - | - | - | - | - | - | - | - | - | - | - |
| CPIJ011127 | *CYP4H34* | **+** | **+** | **+** | **+** | **+** | **+** | **+** | - | - | - | - | - | - | - | - | - | - | - | - |
| CPIJ010546 | *CYP9J34* | **+** | **+** | **+** | **+** | **+** | **+** | **+** | - | - | - | - | - | - | - | - | - | - | - | - |
| CPIJ010544 | *CYP9J33* | **+** | **+** | **+** | **+** | **+** | **+** | **+** | - | - | - | - | - | - | - | - | - | - | - | - |
| CPIJ010543 | *CYP9J40* | **+** | **+** | **+** | **+** | **+** | **+** | **+** | - | - | - | - | - | - | - | - | - | - | - | - |
| CPIJ010542 | *CYP9J38* | **+** | **+** | **+** | **+** | **+** | **+** | **+** | - | - | - | - | - | - | - | - | - | - | - | - |
| CPIJ010538 | *CYP9J46* | **+** | **+** | **+** | **+** | **+** | **+** | **+** | - | - | - | - | - | - | - | - | - | - | - | - |
| CPIJ010537 | *CYP9J45* | **+** | **+** | **+** | **+** | **+** | **+** | **+** | - | - | - | - | - | - | - | - | - | - | - | - |
| CPIJ010227 | *CYP12F13* | **+** | **+** | **+** | **+** | **+** | **+** | **+** | - | - | - | - | - | - | - | - | - | - | - | - |
| CPIJ010225 | *CYP12F14* | **+** | **+** | **+** | **+** | **+** | **+** | **+** | - | - | - | - | - | - | - | - | - | - | - | - |
| CPIJ009478 | *CYP4D42v1* | **+** | **+** | **+** | **+** | **+** | **+** | **+** | - | - | - | - | - | - | - | - | - | - | - | - |
| CPIJ009085 | *CYP6AG13* | **+** | **+** | **+** | **+** | **+** | **+** | **+** | - | - | - | - | - | - | - | - | - | - | - | - |
| CPIJ008566 | *CYP6Z15* | **+** | **+** | **+** | **+** | **+** | **+** | **+** | - | - | - | - | - | - | - | - | - | - | - | - |
| CPIJ007188 | *CYP4H30* | **+** | **+** | **+** | **+** | **+** | **+** | **+** | - | - | - | - | - | - | - | - | - | - | - | - |
| CPIJ006721 | *CYP4H37v 1* | **+** | **+** | **+** | **+** | **+** | **+** | **+** | - | - | - | - | - | - | - | - | - | - | - | - |
| CPIJ005959 | *CYP6AA7* | **+** | **+** | **+** | **+** | **+** | **+** | **+** | - | - | - | - | - | - | - | - | - | - | - | - |
| CPIJ005957 | *CYP6AA9* | **+** | **+** | **+** | **+** | **+** | **+** | **+** | - | - | - | - | - | - | - | - | - | - | - | - |
| CPIJ005956 | *CYP6BZ2* | **+** | **+** | **+** | **+** | **+** | **+** | **+** | - | - | - | - | - | - | - | - | - | - | - | - |
| CPIJ005955 | *CYP6P14* | **+** | **+** | **+** | **+** | **+** | **+** | **+** | - | - | - | - | - | - | - | - | - | - | - | - |
| CPIJ005953 | *CYP6BB3* | **+** | **+** | **+** | **+** | **+** | **+** | **+** | - | - | - | - | - | - | - | - | - | - | - | - |
| CPIJ005952 | *CYP6BB4* | **+** | **+** | **+** | **+** | **+** | **+** | **+** | - | - | - | - | - | - | - | - | - | - | - | - |
| CPIJ002538 | *CYP6AG12* | **+** | **+** | **+** | **+** | **+** | **+** | **+** | - | - | - | - | - | - | - | - | - | - | - | - |
| CPIJ004088 | guanylyl cyclase receptor | **+** | - | **+** | **+** | **+** | - | - | - | - | - | - | - | - | - | - | - | - | - | - |
| CPIJ019428 | trypsin 2 | **+** | - | - | - | - | - | - | **+** | **+** | **+** | **+** | **+** | **+** | **+** | - | - | - | - | - |
| CPIJ019007 | polyserase-2 | **+** | - | - | - | - | - | - | **+** | **+** | **+** | **+** | **+** | **+** | **+** | - | - | - | - | - |
| CPIJ018037 | serine protease | **+** | - | - | - | - | - | - | **+** | **+** | **+** | **+** | **+** | **+** | **+** | - | - | - | - | - |
| CPIJ016102 | transmembrane protease | **+** | - | - | - | - | - | - | **+** | **+** | **+** | **+** | **+** | **+** | **+** | - | - | - | - | - |
| CPIJ016012 | tryptase-2 | **+** | - | - | - | - | - | - | **+** | **+** | **+** | **+** | **+** | **+** | **+** | - | - | - | - | - |
| CPIJ014523 | elastase-3A | **+** | - | - | - | - | - | - | **+** | **+** | **+** | **+** | **+** | **+** | **+** | - | - | - | - | - |
| CPIJ010641 | prostasin | **+** | - | - | - | - | - | - | **+** | **+** | **+** | **+** | **+** | **+** | **+** | - | - | - | - | - |
| CPIJ006543 | urokinase-type plasminogen activator | **+** | - | - | - | - | - | - | **+** | **+** | **+** | **+** | **+** | **+** | **+** | - | - | - | - | - |
| CPIJ006542 | chymotrypsin-2 | **+** | - | - | - | - | - | - | **+** | **+** | **+** | **+** | **+** | **+** | **+** | - | - | - | - | - |
| CPIJ006076 | hypodermin-B | **+** | - | - | - | - | - | - | **+** | **+** | **+** | **+** | **+** | **+** | **+** | - | - | - | - | - |
| CPIJ005272 | trypsin 3A1 | **+** | - | - | - | - | - | - | **+** | **+** | **+** | **+** | **+** | **+** | **+** | - | - | - | - | - |
| CPIJ004594 | conserved hypothetical protein | **+** | - | - | - | - | - | - | **+** | **+** | **+** | **+** | **+** | **+** | **+** | - | - | - | - | - |
| CPIJ003623 | coagulation factor XII | **+** | - | - | - | - | - | - | **+** | **+** | **+** | **+** | **+** | **+** | **+** | - | - | - | - | - |
| CPIJ002156 | chymotrypsin BI | **+** | - | - | - | - | - | - | **+** | **+** | **+** | **+** | **+** | **+** | **+** | - | - | - | - | - |
| CPIJ002142 | chymotrypsin BI | **+** | - | - | - | - | - | - | **+** | **+** | **+** | **+** | **+** | **+** | **+** | - | - | - | - | - |
| CPIJ002140 | chymotrypsin BI | **+** | - | - | - | - | - | - | **+** | **+** | **+** | **+** | **+** | **+** | **+** | - | - | - | - | - |
| CPIJ002139 | HzC4 chymotrypsinogen | **+** | - | - | - | - | - | - | **+** | **+** | **+** | **+** | **+** | **+** | **+** | - | - | - | - | - |
| CPIJ002138 | chymotrypsinogen | **+** | - | - | - | - | - | - | **+** | **+** | **+** | **+** | **+** | **+** | **+** | - | - | - | - | - |
| CPIJ002137 | serine protease1/2 | **+** | - | - | - | - | - | - | **+** | **+** | **+** | **+** | **+** | **+** | **+** | - | - | - | - | - |
| CPIJ002135 | trypsin alpha-4 | **+** | - | - | - | - | - | - | **+** | **+** | **+** | **+** | **+** | **+** | **+** | - | - | - | - | - |
| CPIJ002133 | trypsin epsilon | **+** | - | - | - | - | - | - | **+** | **+** | **+** | **+** | **+** | **+** | **+** | - | - | - | - | - |
| CPIJ002130 | kallikrein-7 | **+** | - | - | - | - | - | - | **+** | **+** | **+** | **+** | **+** | **+** | **+** | - | - | - | - | - |
| CPIJ002128 | mast cell protease 2 | **+** | - | - | - | - | - | - | **+** | **+** | **+** | **+** | **+** | **+** | **+** | - | - | - | - | - |
| CPIJ001979 | conserved hypothetical protein | **+** | - | - | - | - | - | - | **+** | **+** | **+** | **+** | **+** | **+** | **+** | - | - | - | - | - |
| CPIJ001111 | proacrosin | **+** | - | - | - | - | - | - | **+** | **+** | **+** | **+** | **+** | **+** | **+** | - | - | - | - | - |
| CPIJ000617 | clip-domain serine protease | **+** | - | - | - | - | - | - | **+** | **+** | **+** | **+** | **+** | **+** | **+** | - | - | - | - | - |
| CPIJ000616 | clip-domain serine protease | **+** | - | - | - | - | - | - | **+** | **+** | **+** | **+** | **+** | **+** | **+** | - | - | - | - | - |
| CPIJ019029 | metalloproteinase | **+** | - | - | - | - | - | - | **+** | **+** | **+** | **+** | **+** | - | - | **+** | - | - | - | - |
| CPIJ013319 | metalloproteinase | **+** | - | - | - | - | - | - | **+** | **+** | **+** | **+** | **+** | - | - | **+** | - | - | - | - |
| CPIJ010224 | metalloproteinase | **+** | - | - | - | - | - | - | **+** | **+** | **+** | **+** | **+** | - | - | **+** | - | - | - | - |
| CPIJ009594 | nephrosin | **+** | - | - | - | - | - | - | **+** | **+** | **+** | **+** | **+** | - | - | **+** | - | - | - | - |
| CPIJ007383 | endothelin-converting enzyme 1 | **+** | - | - | - | - | - | - | **+** | **+** | **+** | **+** | **+** | - | - | **+** | - | - | - | - |
| CPIJ002945 | zinc metalloproteinase dpy-31 | **+** | - | - | - | - | - | - | **+** | **+** | **+** | **+** | **+** | - | - | **+** | - | - | - | - |
| CPIJ002943 | conserved hypothetical protein | **+** | - | - | - | - | - | - | **+** | **+** | **+** | **+** | **+** | - | - | **+** | - | - | - | - |
| CPIJ002942 | zinc metalloproteinase nas-12 | **+** | - | - | - | - | - | - | **+** | **+** | **+** | **+** | **+** | - | - | **+** | - | - | - | - |
| CPIJ002941 | high choriolytic enzyme 1 | **+** | - | - | - | - | - | - | **+** | **+** | **+** | **+** | **+** | - | - | **+** | - | - | - | - |
| CPIJ012036 | aminopeptidase N | **+** | - | - | - | - | - | - | **+** | **+** | **+** | - | - | - | - | **+** | **+** | - | - | - |
| CPIJ010805 | carboxypeptidase A1 | **+** | - | - | - | - | - | - | **+** | **+** | **+** | - | - | - | - | **+** | **+** | - | - | - |
| CPIJ009106 | angiotensin-converting enzyme | **+** | - | - | - | - | - | - | **+** | **+** | **+** | - | - | - | - | **+** | **+** | - | - | - |
| CPIJ004086 | angiotensin-converting enzyme | **+** | - | - | - | - | - | - | **+** | **+** | **+** | - | - | - | - | **+** | **+** | - | - | - |
| CPIJ001745 | zinc carboxypeptidase | **+** | - | - | - | - | - | - | **+** | **+** | **+** | - | - | - | - | **+** | **+** | - | - | - |
| CPIJ001744 | zinc carboxypeptidase | **+** | - | - | - | - | - | - | **+** | **+** | **+** | - | - | - | - | **+** | **+** | - | - | - |
| CPIJ001743 | carboxypeptidase A2 | **+** | - | - | - | - | - | - | **+** | **+** | **+** | - | - | - | - | **+** | **+** | - | - | - |
| CPIJ001742 | zinc carboxypeptidase | **+** | - | - | - | - | - | - | **+** | **+** | **+** | - | - | - | - | **+** | **+** | - | - | - |
| CPIJ008876 | lysosomal pro-X carboxypeptidase | **+** | - | - | - | - | - | - | **+** | **+** | **+** | - | - | **+** | **+** | - | **+** | - | - | - |
| CPIJ008873 | prolylcarboxypeptidase | **+** | - | - | - | - | - | - | **+** | **+** | **+** | - | - | **+** | **+** | - | **+** | - | - | - |
| CPIJ001240 | cathepsin B-like thiol protease | **+** | - | - | - | - | - | - | **+** | **+** | **+** | **+** | - | - | - | - | - | - | - | - |
| CPIJ001239 | cathepsin B | **+** | - | - | - | - | - | - | **+** | **+** | **+** | **+** | - | - | - | - | - | - | - | - |
| CPIJ001050 | protease m1 zinc metalloprotease | **+** | - | - | - | - | - | - | **+** | **+** | **+** | - | - | - | - | **+** | - | - | - | - |
| CPIJ014110 | conserved hypothetical protein | **+** | - | - | - | - | - | - | **+** | **+** | - | - | - | - | - | - | - | - | - | - |
| CPIJ009738 | conserved hypothetical protein | **+** | - | - | - | - | - | - | **+** | **+** | - | - | - | - | - | - | - | - | - | - |
| CPIJ010716 | luciferin 4-monooxygenase | **+** | **+** | - | - | - | - | **+** | - | - | - | - | - | - | - | - | - | - | - | - |
| CPIJ005187 | phenoloxidase subunit 1 | **+** | **+** | - | - | - | **+** | - | - | - | - | - | - | - | - | - | - | - | - | - |
| CPIJ019598 | basic endochitinase CHB4 | **+** | - | - | - | - | - | - | **+** | - | - | - | - | - | - | - | - | **+** | **+** | - |
| CPIJ018802 | endochitinase A | **+** | - | - | - | - | - | - | **+** | - | - | - | - | - | - | - | - | **+** | **+** | - |
| CPIJ009306 | neutral alpha-glucosidase ab | **+** | - | - | - | - | - | - | **+** | - | - | - | - | - | - | - | - | **+** | **+** | - |
| CPIJ008904 | alpha-glucosidase | **+** | - | - | - | - | - | - | **+** | - | - | - | - | - | - | - | - | **+** | **+** | - |
| CPIJ008528 | glycoside hydrolase | **+** | - | - | - | - | - | - | **+** | - | - | - | - | - | - | - | - | **+** | **+** | - |
| CPIJ006585 | glycoprotein | **+** | - | - | - | - | - | - | **+** | - | - | - | - | - | - | - | - | **+** | **+** | - |
| CPIJ006166 | deltamethrin resistance-associated NYD-GBE | **+** | - | - | - | - | - | - | **+** | - | - | - | - | - | - | - | - | **+** | **+** | - |
| CPIJ005451 | lysozyme | **+** | - | - | - | - | - | - | **+** | - | - | - | - | - | - | - | - | **+** | **+** | - |
| CPIJ004323 | gram-negative bacteria binding protein | **+** | - | - | - | - | - | - | **+** | - | - | - | - | - | - | - | - | **+** | **+** | - |
| CPIJ004320 | gram-negative bacteria-binding protein 1 | **+** | - | - | - | - | - | - | **+** | - | - | - | - | - | - | - | - | **+** | **+** | - |
| CPIJ002104 | plasma alpha-L-fucosidase | **+** | - | - | - | - | - | - | **+** | - | - | - | - | - | - | - | - | **+** | **+** | - |
| CPIJ005725 | alpha-amylase A | **+** | - | - | - | - | - | - | **+** | - | - | - | - | - | - | - | - | **+** | - | - |
| CPIJ005060 | alpha-amylase B | **+** | - | - | - | - | - | - | **+** | - | - | - | - | - | - | - | - | **+** | - | - |
| CPIJ019948 | myosin vii | **+** | - | - | - | - | - | - | **+** | - | - | - | - | - | - | - | - | - | - | - |
| CPIJ019917 | triacylglycerol lipase | **+** | - | - | - | - | - | - | **+** | - | - | - | - | - | - | - | - | - | - | - |
| CPIJ018233 | carboxylesterase | **+** | - | - | - | - | - | - | **+** | - | - | - | - | - | - | - | - | - | - | - |
| CPIJ018231 | carboxylesterase | **+** | - | - | - | - | - | - | **+** | - | - | - | - | - | - | - | - | - | - | - |
| CPIJ017110 | fumarylacetoacetate hydrolase | **+** | - | - | - | - | - | - | **+** | - | - | - | - | - | - | - | - | - | - | - |
| CPIJ016336 | esterase B1 | **+** | - | - | - | - | - | - | **+** | - | - | - | - | - | - | - | - | - | - | - |
| CPIJ015649 | DNA-binding protein smubp-2 | **+** | - | - | - | - | - | - | **+** | - | - | - | - | - | - | - | - | - | - | - |
| CPIJ013085 | sarcalumenin | **+** | - | - | - | - | - | - | **+** | - | - | - | - | - | - | - | - | - | - | - |
| CPIJ007824 | esterase B1 | **+** | - | - | - | - | - | - | **+** | - | - | - | - | - | - | - | - | - | - | - |
| CPIJ007461 | epoxide hydrolase | **+** | - | - | - | - | - | - | **+** | - | - | - | - | - | - | - | - | - | - | - |
| CPIJ007035 | lipase | **+** | - | - | - | - | - | - | **+** | - | - | - | - | - | - | - | - | - | - | - |
| CPIJ006560 | peptidoglycan recognition protein-lc | **+** | - | - | - | - | - | - | **+** | - | - | - | - | - | - | - | - | - | - | - |
| CPIJ004695 | dynein-1-beta heavy chain | **+** | - | - | - | - | - | - | **+** | - | - | - | - | - | - | - | - | - | - | - |
| CPIJ004222 | pancreatic triacylglycerol lipase | **+** | - | - | - | - | - | - | **+** | - | - | - | - | - | - | - | - | - | - | - |
| CPIJ002103 | conserved hypothetical protein | **+** | - | - | - | - | - | - | **+** | - | - | - | - | - | - | - | - | - | - | - |
| CPIJ002067 | vacuolar ATP synthase subunit C | **+** | - | - | - | - | - | - | **+** | - | - | - | - | - | - | - | - | - | - | - |
| CPIJ001520 | multidrug resistance-associated protein 1 | **+** | - | - | - | - | - | - | **+** | - | - | - | - | - | - | - | - | - | - | - |
| CPIJ000853 | myosin heavy chain | **+** | - | - | - | - | - | - | **+** | - | - | - | - | - | - | - | - | - | - | - |
| CPIJ000852 | myosin-Id | **+** | - | - | - | - | - | - | **+** | - | - | - | - | - | - | - | - | - | - | - |
| CPIJ003495 | fatty acid synthase S-acetyltransferase | **+** | **+** | - | - | - | - | - | **+** | - | - | - | - | - | - | - | - | - | - | - |
| CPIJ014287 | ferritin heavy chain | **+** | **+** | - | - | **+** | - | - | - | - | - | - | - | - | - | - | - | - | - | - |
| CPIJ005308 | conserved hypothetical protein | - | - | **+** | **+** | **+** | - | - | - | - | - | - | - | - | - | - | - | - | - | - |
| CPIJ004595 | cytochrome b5 | - | - | **+** | **+** | **+** | - | - | - | - | - | - | - | - | - | - | - | - | - | - |
| CPIJ004125 | succinate dehydrogenase | - | - | **+** | **+** | **+** | - | - | - | - | - | - | - | - | - | - | - | - | - | - |
| CPIJ018869 | NADH dehydrogenase iron-sulfur protein 7, mitoch. | **+** | **+** | - | - | - | - | - | - | - | - | - | - | - | - | - | - | - | - | - |
| CPIJ016440 | dihydroceramide delta (4)-desaturase | **+** | **+** | - | - | - | - | - | - | - | - | - | - | - | - | - | - | - | - | - |
| CPIJ016322 | alkyldihydroxyacetonephosphate synthase | **+** | **+** | - | - | - | - | - | - | - | - | - | - | - | - | - | - | - | - | - |
| CPIJ016321 | alkyldihydroxyacetonephosphate synthase | **+** | **+** | - | - | - | - | - | - | - | - | - | - | - | - | - | - | - | - | - |
| CPIJ013647 | alkyldihydroxyacetonephosphate synthase | **+** | **+** | - | - | - | - | - | - | - | - | - | - | - | - | - | - | - | - | - |
| CPIJ009438 | aldehyde dehydrogenase | **+** | **+** | - | - | - | - | - | - | - | - | - | - | - | - | - | - | - | - | - |
| CPIJ007620 | choline dehydrogenase | **+** | **+** | - | - | - | - | - | - | - | - | - | - | - | - | - | - | - | - | - |
| CPIJ005656 | oxidoreductase | **+** | **+** | - | - | - | - | - | - | - | - | - | - | - | - | - | - | - | - | - |
| CPIJ004600 | oxidoreductase | **+** | **+** | - | - | - | - | - | - | - | - | - | - | - | - | - | - | - | - | - |
| CPIJ004379 | steroid dehydrogenase | **+** | **+** | - | - | - | - | - | - | - | - | - | - | - | - | - | - | - | - | - |
| CPIJ003802 | NADP-dependent leukotriene B4 12-hydroxydehydrog. | **+** | **+** | - | - | - | - | - | - | - | - | - | - | - | - | - | - | - | - | - |
| CPIJ003059 | acyl-CoA oxidase | **+** | **+** | - | - | - | - | - | - | - | - | - | - | - | - | - | - | - | - | - |
| CPIJ001318 | d-lactate dehydrognease 2 | **+** | **+** | - | - | - | - | - | - | - | - | - | - | - | - | - | - | - | - | - |
| CPIJ018631 | glutathione-s-transferase theta, gst | **+** | - | - | - | - | - | - | - | - | - | - | - | - | - | - | - | - | - | - |
| CPIJ015996 | ecdysteroid UDP-glucosyltransferase | **+** | - | - | - | - | - | - | - | - | - | - | - | - | - | - | - | - | - | - |
| CPIJ015088 | 4-coumarate-CoA ligase 1 | **+** | - | - | - | - | - | - | - | - | - | - | - | - | - | - | - | - | - | - |
| CPIJ014577 | phosphoglycerate mutase 2 | **+** | - | - | - | - | - | - | - | - | - | - | - | - | - | - | - | - | - | - |
| CPIJ012763 | 3-phosphoinositide-dependent protein kinase 1 | **+** | - | - | - | - | - | - | - | - | - | - | - | - | - | - | - | - | - | - |
| CPIJ011827 | conserved hypothetical protein | **+** | - | - | - | - | - | - | - | - | - | - | - | - | - | - | - | - | - | - |
| CPIJ009929 | conserved hypothetical protein | **+** | - | - | - | - | - | - | - | - | - | - | - | - | - | - | - | - | - | - |
| CPIJ009094 | ornithine decarboxylase 1 | **+** | - | - | - | - | - | - | - | - | - | - | - | - | - | - | - | - | - | - |
| CPIJ008853 | maltose phosphorylase | **+** | - | - | - | - | - | - | - | - | - | - | - | - | - | - | - | - | - | - |
| CPIJ008110 | conserved hypothetical protein | **+** | - | - | - | - | - | - | - | - | - | - | - | - | - | - | - | - | - | - |
| CPIJ007538 | arginine kinase | **+** | - | - | - | - | - | - | - | - | - | - | - | - | - | - | - | - | - | - |
| CPIJ006619 | cystathionine gamma-lyase | **+** | - | - | - | - | - | - | - | - | - | - | - | - | - | - | - | - | - | - |
| CPIJ006508 | UDP-glucuronosyltransferase 2B4 | **+** | - | - | - | - | - | - | - | - | - | - | - | - | - | - | - | - | - | - |
| CPIJ006459 | long-chain-fatty-acid-CoA ligase | **+** | - | - | - | - | - | - | - | - | - | - | - | - | - | - | - | - | - | - |
| CPIJ006339 | receptor of activated protein kinase C 1 | **+** | - | - | - | - | - | - | - | - | - | - | - | - | - | - | - | - | - | - |
| CPIJ006160 | glutathione s-transferase | **+** | - | - | - | - | - | - | - | - | - | - | - | - | - | - | - | - | - | - |
| CPIJ004867 | conserved hypothetical protein | **+** | - | - | - | - | - | - | - | - | - | - | - | - | - | - | - | - | - | - |
| CPIJ003692 | glucosyl/glucuronosyl transferase | **+** | - | - | - | - | - | - | - | - | - | - | - | - | - | - | - | - | - | - |
| CPIJ002663 | glutathione S-transferase 1-1 | **+** | - | - | - | - | - | - | - | - | - | - | - | - | - | - | - | - | - |  |
| CPIJ001427 | conserved hypothetical protein | **+** | - | - | - | - | - | - | - | - | - | - | - | - | - | - | - | - | - | - |
| CPIJ001091 | lactosylceramide 4-alpha-galactosyltransferase | **+** | - | - | - | - | - | - | - | - | - | - | - | - | - | - | - | - | - | - |
| CPIJ000791 | conserved hypothetical protein | **+** | - | - | - | - | - | - | - | - | - | - | - | - | - | - | - | - | - | - |
| CPIJ018825 | larval serum protein 1 beta chain | - | - | - | - | - | - | - | - | - | - | - | - | - | - | - | - | - | - | **+** |
| CPIJ018824 | larval serum protein 1 beta chain | - | - | - | - | - | - | - | - | - | - | - | - | - | - | - | - | - | - | **+** |
| CPIJ009032 | larval serum protein 2 | - | - | - | - | - | - | - | - | - | - | - | - | - | - | - | - | - | - | **+** |
| CPIJ007783 | arylphorin subunit alpha | - | - | - | - | - | - | - | - | - | - | - | - | - | - | - | - | - | - | **+** |
| CPIJ006538 | larval serum protein 1 beta chain | - | - | - | - | - | - | - | - | - | - | - | - | - | - | - | - | - | - | **+** |
| CPIJ006537 | larval serum protein 1 beta chain | - | - | - | - | - | - | - | - | - | - | - | - | - | - | - | - | - | - | **+** |
| CPIJ001820 | larval serum protein 2 | - | - | - | - | - | - | - | - | - | - | - | - | - | - | - | - | - | - | **+** |
| CPIJ000056 | larval serum protein 1 beta chain | - | - | - | - | - | - | - | - | - | - | - | - | - | - | - | - | - | - | **+** |

^†^Genes within the functionally enriched GO terms for the differentially upregulated gene set in HAmCq^G8^ when tested by gProfiler (http://biit.cs.ut.ee/gprofiler/gcocoa.cgi).

^*^*Culex quinquefasciatus* genome, Johannesburg strain CpipJ1.2, June 2008; http://cquinquefasciatus.vectorbase.org/Annotations for cytochrome P450 genes were taken from the most current annotation based on: Nelson, DR (2009) The Cytochrome P450 Homepage. Human Genomics 4, 59-65: http://drnelson.uthsc.edu/CytochromeP450.html

^**^Gene Ontology consortium (version 1.2084; release date: 12:07:2011)

**^‡^** A “+” sign indicates GO terms within column that are assigned to the gene in the list, while a “-” sign indicates that GO terms within the column are not assigned to the gene within the list.
